# Multiple site place-of-care manufactured anti-CD19 CAR-T cells induce high remission rates in B-cell malignancy patients

**DOI:** 10.1038/s41467-021-27312-6

**Published:** 2021-12-10

**Authors:** Michael Maschan, Paolo F. Caimi, Jane Reese-Koc, Gabriela Pacheco Sanchez, Ashish A. Sharma, Olga Molostova, Larisa Shelikhova, Dmitriy Pershin, Alexey Stepanov, Yakov Muzalevskii, Vinicius G. Suzart, Folashade Otegbeye, David Wald, Ying Xiong, Darong Wu, Adam Knight, Ibe Oparaocha, Beatrix Ferencz, Andre Roy, Andrew Worden, Winfried Kruger, Michael Kadan, Dina Schneider, Rimas Orentas, Rafick-Pierre Sekaly, Marcos de Lima, Boro Dropulić

**Affiliations:** 1 Dmitriy Rogachev National Medical Research Centre of Pediatric Hematology, Oncology and Immunology, Moscow, Russian Federation; 2 University Hospitals Seidman Cancer Center, Case Western Reserve University, Cleveland, OH USA; 3 Emory University, Atlanta, GA USA; 4 Shemyakin-Ovchinnikov Institute of Bioorganic Chemistry, Moscow, Russia; 5 Lentigen, A Miltenyi Biotec Company, Gaithersburg, MD USA; 6 Caring Cross, Gaithersburg, MD USA; 7 Seattle Children’s Hospital, and Ben Towne Center for Childhood Cancer Research, Seattle Children’s Research Institute, Seattle, WA USA; 8 Department of Pediatrics, University of Washington School of Medicine, Seattle, WA USA; 9 Present Address: Cleveland Clinic, Cleveland, OH USA; 10 Present Address: Ohio State University, Columbus, OH USA

**Keywords:** Translational immunology, Translational research, Cancer

## Abstract

Chimeric antigen receptor (CAR) T cells targeting the CD19 antigen are effective in treating adults and children with B-cell malignancies. Place-of-care manufacturing may improve performance and accessibility by obviating the need to cryopreserve and transport cells to centralized facilities. Here we develop an anti-CD19 CAR (CAR19) comprised of the 4-1BB co-stimulatory and TNFRSF19 transmembrane domains, showing anti-tumor efficacy in an in vivo xenograft lymphoma model. CAR19 T cells are manufactured under current good manufacturing practices (cGMP) at two disparate clinical sites, Moscow (Russia) and Cleveland (USA). The CAR19 T-cells is used to treat patients with relapsed/refractory pediatric B-cell Acute Lymphocytic Leukemia (ALL; *n* = 31) or adult B-cell Lymphoma (NHL; *n* = 23) in two independently conducted phase I clinical trials with safety as the primary outcome (NCT03467256 and NCT03434769, respectively). Probability of measurable residual disease-negative remission was also a primary outcome in the ALL study. Secondary outcomes include complete remission (CR) rates, overall survival and median duration of response. CR rates are 89% (ALL) and 73% (NHL). After a median follow-up of 17 months, one-year survival rate of ALL complete responders is 79.2% (95%CI 64.5‒97.2%) and median duration of response is 10.2 months. For NHL complete responders one-year survival is 92.9%, and median duration of response has not been reached. Place-of-care manufacturing produces consistent CAR-T cell products at multiple sites that are effective for the treatment of patients with B-cell malignancies.

## Abstract (web summary)

Strategies to address the challenges associated with product manufacturing can improve chimeric antigen receptor (CAR) cell–based therapeutics. Here the authors report the results of two clinical trials in patients with B-cell malignancies, showing that place-of-care manufacturing has a low production failure rate with CD19-directed CAR-T cell products inducing high remission rates.

## Introduction

Pediatric B-acute lymphoblastic leukemia (B-ALL) is highly curable, but 2–3% of patients have refractory disease and 10–15% will relapse after chemotherapy^1–4^. Non-Hodgkin’s lymphoma (NHL) is often successfully treated with combination chemoimmunotherapy^5–7^. However, patients who are resistant to initial chemotherapy have a poor prognosis^8^. CD19 is a 95 kDa glycoprotein present on the B-cell surface that is expressed by most B-cell malignancies including NHL and ALL^9,10^. CD19-targeting immunotherapies such asbispecific antibodies and CAR T cells have induced remission in children and adults with relapsed/refractory B-ALL and NHL^11–18^.

The remissions in pediatric B-ALL and adult diffuse large B cell lymphoma (DLCL) led to FDA authorization for Kymriah^TM^ (tisagenlecleucel, Novartis) and Yescarta^TM^ (axicabtageneciloleucel, Gilead)^12,19,20^. However, in a phase 2 study of tisagenlecleucel in pediatric and young adult patients with CD19 + ALL, 7.6% of patients did not receive treatment due to manufacturing failures^12^. Additionally, in a study using the same CAR construct in adults with lymphoma, 13.2% of patients did not receive treatment due to manufacturing failure, which has been reported in 3–13% of commercially manufactured products^19^. Another significant proportion of patients were not treated due to disease progression^12^. These studies suggest that alternative modalities to centralized manufacturing are needed.

Here, we report the development of a CD19-targeting CAR comprised of the TNF receptor superfamily member 19 (TNFRSF19) transmembrane domain, termed CAR19. CAR19 demonstrates robust expression in primary human T cells and shows potent target-specific cytotoxicity and cytokine response in vitro and is effective in rejecting CD19+ tumors in an in vivo xenograft NSG lymphoma model. Furthermore, robust clinical responses are observed in patients with relapsed/refractory B cell malignancies treated with place-of-care manufactured CAR19-T cells in two different locations. Place-of-care manufacturing is defined as near the point of patient treatment allowing cell products to be produced and infused without the need for cryopreservation. The results of this study support the safety and efficacy of this approach.

## Results

### Design of the chimeric antigen receptor targeting CD19

To identify new T cell functionality associated with alterations in structural and transmembrane sequence we carried homology searches, restricted to human proteins, to identify cell surface glycoproteins analogous to CD8 transmembrane domain amino acid sequence (BLAST^®^, NCBI, NIH)^21^. Homology search results were annotated to reflect known lymphocyte cell surface proteins, and although sequence diversity was greater than anticipated, motifs in common (highlighted in Supplementary Fig. 1) could be identified in TNFRSF9 (CD137), TNFRSF16 (CD271, NGFR, or commonly LNGFR), and TNFRSF19 (TROY). Transmembrane domains used were as predicted by TMHMM 2.0 (Prediction of transmembrane helices in proteins, DTU Health Tech, Denmark). Each of these transmembrane domains were linked at C-terminus of the spacer domains, which joined them to the FMC63 CD19-binder. CD8 linker joined to CD8TM, CD4TM, TNFRSF19, TNFRSF16, and TNFRSF9 were then tested for surface expression and killing of CD19+ leukemia cell lines. Also tested were sequence length equivalent TNFRSF16, TNFRSF9, and TNFRSF19 linker domains, linked to their native transmembrane (TM) domain. Of note, TNFRSH16 gave the highest cell surface expression as determined by flow cytometry analysis, but was not functional in cytotoxicity assays, and did not control Raji cell growth in an NSG mouse model. Only CD8 linker combined with TM-TNFRSF19 showed equivalent or better activity compared to constructs encoding CD8 linker and TM domains in either in vitro or NSG mouse models of anti-leukemic activity^22^.

**Fig. 1:**
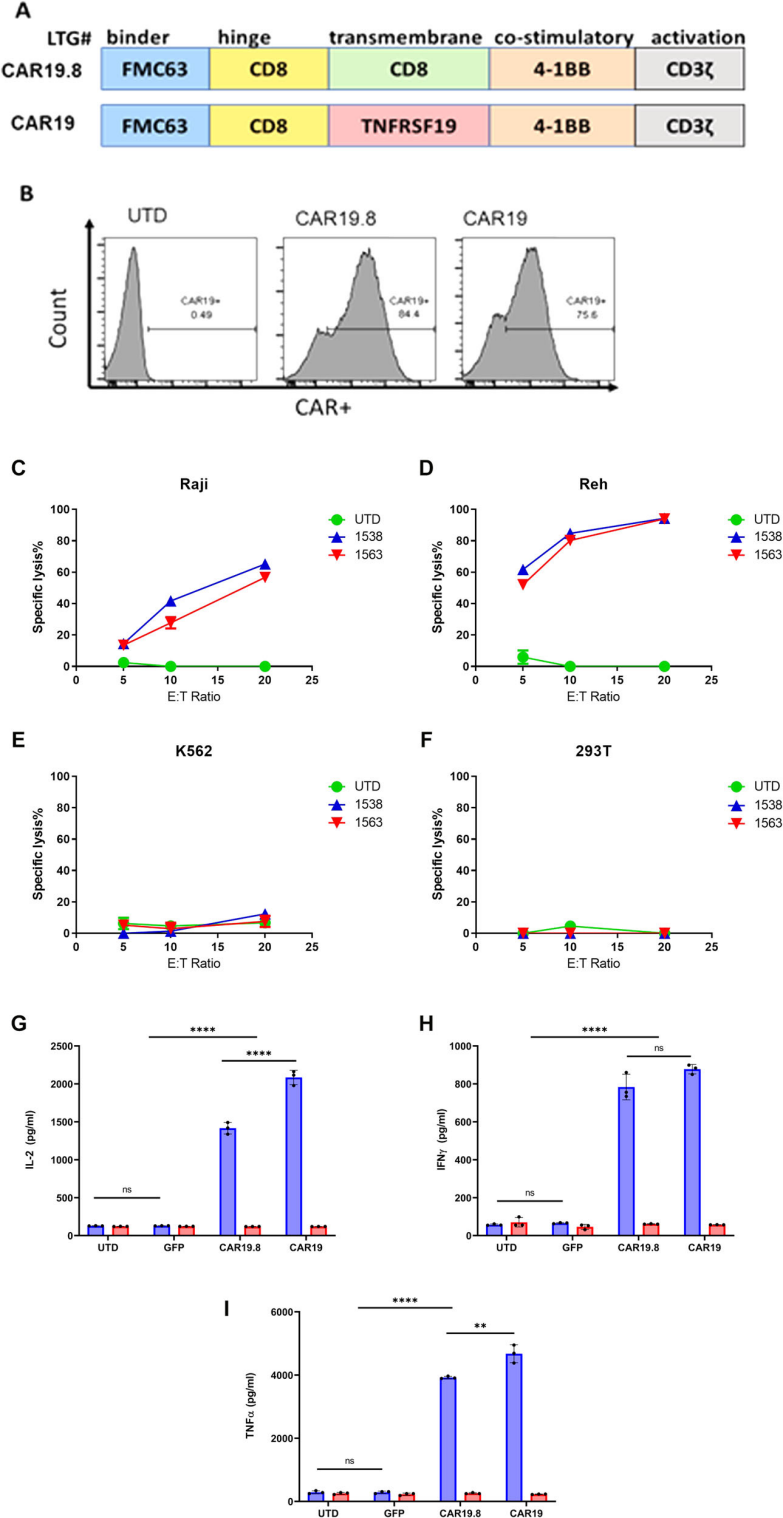
CAR19 design, expression in human primary T cells, cytotoxicity in vitro, and tumor-specific cytokine response in vitro. Schematic representation of CAR19.8 and CAR19 (**A**). CAR19.8 has a CD19-targeting ScFv sequence FMC63, CD8 hinge sequence, CD8 transmembrane region sequence, a co-stimulatory domain derived from 4-1BB/CD137, and a CD3ζ activation domain. CAR19 has a CD19-targeting ScFv sequence FMC63 in frame with CD8 hinge sequence, a transmembrane region sequence from TNFRSF19, a co-stimulatory domain derived from 4-1BB/CD137, and a CD3ζ activation domain. **B** Representative detection of anti-CD19 CARs. Target-specific cytotoxicity was evaluated by co-incubation of CAR19, or comparator CAR19.8 with target cell lines Raji (**C**), Reh (**D**), K562 (**E**), and 293T (**F**) for 18 h at effector to target ratio 5:1, 10:1, or 20:1, in triplicate, red lines represent CAR19, blue lines represent CAR19.8 and green line represents untransduced (UTD). Then, target cell lysis was analyzed by luminometry. Sample means were compared by one-way ANOVA followed by Tukey’s multiple comparisons test. IL-2 (**G**), IFNγ (**H**), and TNFα (**I**) cytokine secretion analysis was performed on supernatants from CAR T cells challenged overnight with Raji lymphoma cells at effector to target ratio 10:1, in triplicate, and measured by ELISA, blue bars represent Raji cells, and red bars represent CAR alone. Data are representative of three independent experiments, each performed on CAR T cells produced from a different donor, bars represent mean, error bars represent SD. Sample means were compared by One-way ANOVA followed by Tukey’s multiple comparisons test. ****p < 0.0001, **p < 0.01, ns-non-significant. Source data are provided as a Source Data File for panels **c**–**i**.

CAR19 was therefore constructed by combining CD19-targeting single chain fragment variable (ScFv) sequence FMC63 in frame with CD8 hinge sequence, a transmembrane region sequence from TNFRSF19, a co-stimulatory domain derived from 4-1BB/CD137, and a CD3ζ activation domain (Fig. 1A). All sequences except for the FMC63 scFv, which was derived from heavy and light chains of a murine antibody, are of human origin. We compared CAR19 to its predecessor, which comprised the same sequences except the TNFRSF19 transmembrane domain was substituted for the CD**8** transmembrane domain (CAR19.**8**; Fig. 1A).

### CAR-T cells expressing variant CAR19 have similar anti-leukemic effects

Having confirmed the expression of CAR19 in primary human T cells, we next evaluated its anti-leukemic effects on CD19+ cancer cell lines Raji (NHL), Reh (ALL), or with CD19‒ control lines K562 or 293T (Fig. 1C–F, respectively). Target cell lines were stably transduced to express firefly luciferase, thus enabling assessment of target cell killing by luminometry. CAR-T cell killing of target cells at three effector to target ratios (E:T) of 5:1, 10:1, and 20:1 were analyzed. In CD19+ target lines, Raji and Reh, CAR19 and CAR19.8 both demonstrated effective, E:T ratio-dependent target killing. By contrast, UTD control had no killing capacity against Raji or Reh, underscoring that the killing was indeed CAR-dependent (Fig. 1C–F). By contrast, when CAR-T cells were incubated with CD19-negative target lines K562 and 293T, no target killing occurred in either CAR19 or CAR19.8 groups. Therefore, the cytotoxic function of CAR19 and CAR19.8 in vitro was target-dependent (Fig. 1C–F).

Next, we examined the capacity of CAR-T cells to produce cytokines after exposure to target cells. Co-culture media supernatants were harvested following overnight incubation of CAR-T cells with Raji cell targets at E:T ratio of 10:1 and analyzed by ELISA for T cell inflammatory and homeostatic cytokines crucial for CAR-T cell function: IL-2, IFNγ, and TNFα (Fig. 1G–I, respectively). T cells transduced with lentiviral vector to express GFP, and UTD, were included as negative controls. In addition, CAR-T cell alone control, consisting of CAR-T cells incubated under same conditions as co-incubated groups, but in the absence of target cells, was utilized (Fig. 1I). CAR19 and CAR19.8 yielded robust, statistically significant increases in the levels of IL-2, IFNγ, and TNFα in culture media after co-incubation with the CD19+ Raji target cells, whereas cytokine levels in the negative control groups UTD and GFP remained at baseline. A small but statistically significant increase in the production of IL-2 and TNF-alpha by CAR19 was noted (Fig. 1G–I). Importantly, in CAR-T cell alone test groups, no increases in the levels of IL-2, IFNγ, or TNFα were observed, indicating that cytokine induction requires the presence of Raji cell targets, and there was no evidence of tonic CAR-T cell signaling (Fig. 1G–I).

### CAR T cells expressing variant CAR19 molecules control Raji lymphoma tumors in NSG xenograft model

**Fig. 2:**
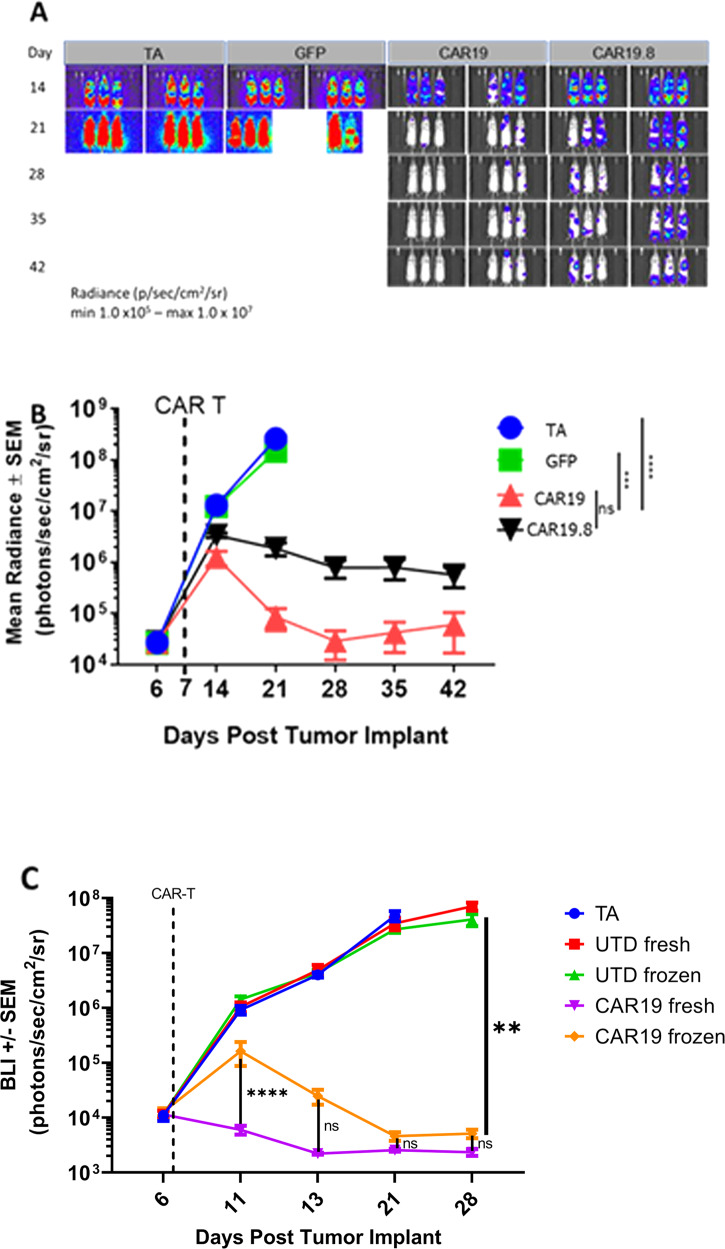
CAR19 controls Raji lymphoma tumors in NSG xenograft model. Xenograft model demonstrating clearance of Raji lymphoma tumors by CAR19 or CAR19.8 (**A**). Graph depicting change in mean radiance over time (**B**). NSG mice were engrafted with Raji-luc tumor cells at 5 × 10^5^ Raji/mouse. On day 7 mice were dosed with 5 × 10^6^ CAR T cells. Xenograft model demonstrating clearance of Raji lymphoma tumors by fresh vs. frozen CAR19 T cells, blue line represents tumor alone (TA), green line represents GFP expressing CAR-T cells, red line represents CAR-T cells transduced with CAR19 vector and black line represents CAR-T cells transduced with CAR19.8 vector (**C**). Fresh CAR19-T cells reduce tumor burden faster in vivo than cryopreserved CAR19-T cells. NSG mice were engrafted with Raji-luc tumor cells at 5 × 10^5^ Raji/mouse. On day 7 mice were dosed with 2 × 10^6^ CAR T+ cells. Tumor burden was assessed by bioluminescent imaging. Blue line represents tumor alone, red line represents fresh untransduced T cells, green line represents thawed frozen untransduced T cells, orange line represents thawed frozen CAR-T cells transduced with CAR19 vector and violet color represents fresh CAR-T cells transduced with CAR19 vector. *N* = 5 mice/group, mean ± SEM. Groups were compared by two-way ANOVA, followed by Tukey’s multiple comparison test for days 6–21, for which observations in all groups were available. ****p < 0.0001, **p < 0.001. Source data are provided as a Source Data File for panels **B** and **C**.

Having established the in vitro functionality of the variant CAR19-T cells, we proceeded to examine their potential efficacy in vivo. NSG mice were implanted with Raji-luc lymphoma on study day 0. Seven days after tumor implant, five million of previously cryopreserved CAR19 or CAR19.8-T cells were administered to each mouse. Tumor progression was monitored by bioluminescent IVIS imaging with tumor alone (TA) and cells transduced with GFP-expressing lentiviral vector (GFP) serving as negative controls (Fig. 2A). Both CAR19 and CAR19.8-T cell groups were able to control tumor burden throughout the study (Fig. 2B).

### Fresh CAR19-T cells reduce tumor burden faster in vivo in NSG mice than cryopreserved CAR19-T cells

Having determined the anti-tumor potency of the designedCAR19-T cells in vivo, we evaluated whether freshly prepared vs. cryopreserved CAR19-T cells impacts the rate or the magnitude of tumor regression in vivo (Fig. 2C). CAR19 T cells, with highly similar transduction efficiencies and CD4:CD8 ratios were either administered fresh, or frozen and thawed prior to administration to animals. Administration of fresh CAR19 T cells yielded significantly lower tumor burden as compared to dose-matched frozen CAR19 T cells on study days 11 and 13 (*p* < 0.005, *t* test). On study days 21 and 28, tumors were similarly and potently rejected by both fresh and frozen cell-treated groups. However, fresh cells reduced the tumor burden sooner by 8 days, and immediately started reducing the tumor burden, while frozen CAR19-T cells first permitted tumor growth before controlling growth (Fig. 2C).

### Place-of-care manufacturing of CAR19-T cells has a low cell production failure rate

1 × 10^8^ CD3^+^ lymphocytes were collected successfully by leukapheresis from all enrolled patients and loaded into the CliniMACS Prodigy^®^. Manufacturing success rate was 96% (*n* = 30) and 100% in the Moscow and Cleveland cohort, respectively (Supplementary Fig. 2A, B). Except for two patients in Cleveland, all CAR19 cells were infused fresh. CAR19 from both cohorts had similar cellular composition with over 90% CD3+ cells. The pediatric cohort had similar CD4:CD8 ratio while the NHL cohort had a higher but not statistically significant CD4:CD8 T cell ratio (Supplementary Fig. 2A). Seven patients in the NHL cohort were manufactured in 8 days.

Median transduction rate was 60% and 48%, respectively, in Moscow and Cleveland (Supplementary Fig. 2B). The median apheresis to infusion time was 13 days in both cohorts (Supplementary Fig. 2A, B). After infusion, CAR19-T levels peaked between days 14–21 (Supplementary Fig. 2C, D) and were not influenced by cell dose (which did not influence CAR19 persistence).

### CAR19-T cells demonstrate robust anti-tumor activity in ALL and NHL patients

**Fig. 3:**
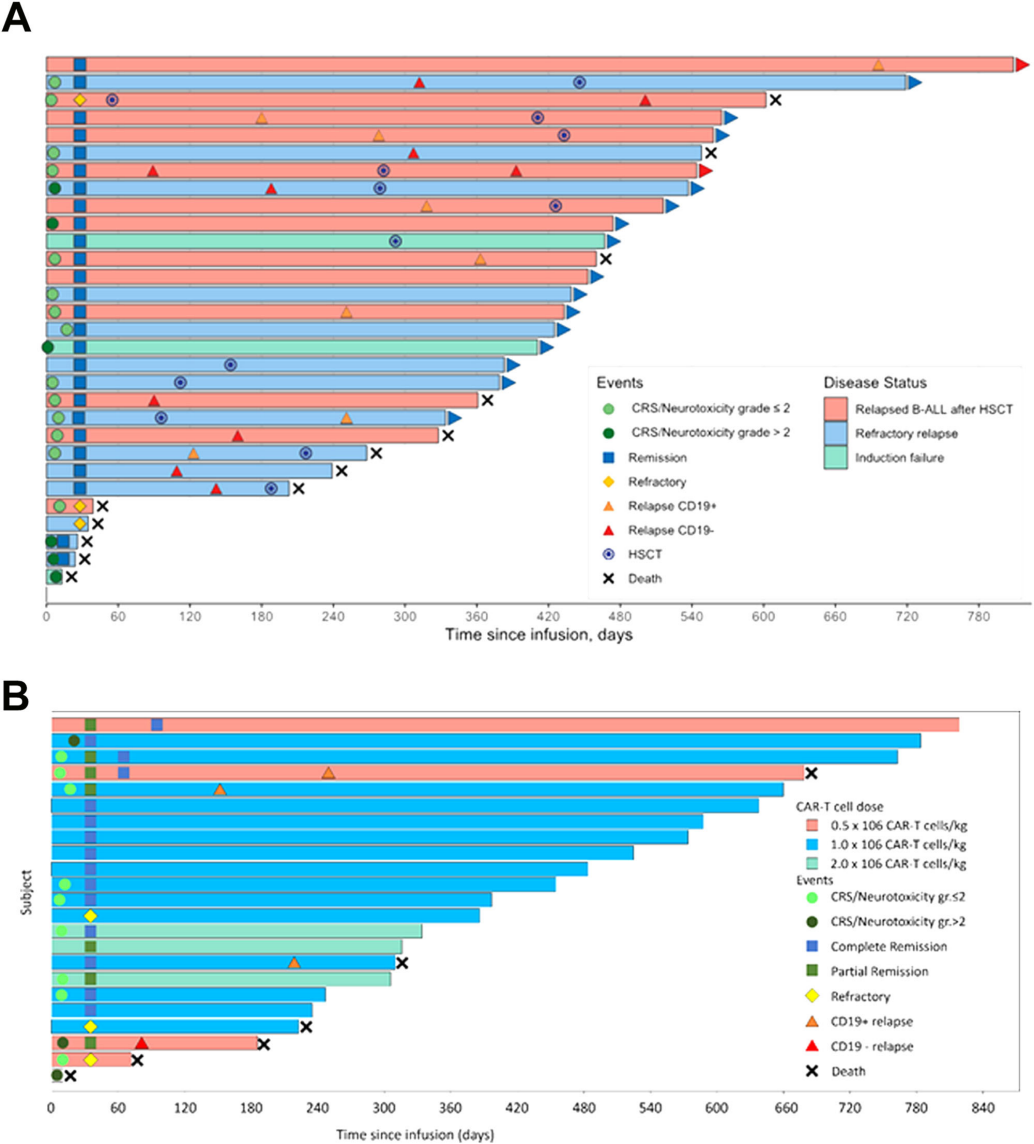
CAR19 is active against pediatric ALL and adult lymphoma. **A** ALL patients (*n* = 31). Horizontal bars correspond to each individual patient; bar color corresponds to type of indication (pink—ALL relapsed after HSCT, sky blue—refractory relapse, green—induction failure); blue squares represent complete remission on day 28; yellow diamonds—persistent disease on day 28; light green circle—either CRS or ICANS < / = grade 2; dark green circle—either CRS or ICANS > grade 2; orange triangle—CD19-positive relapse; red triangle—CD19-positive relapse; black cross—death**. B** NHL patients (*n* = 23). Horizontal bars correspond to each individual patient; bar color corresponds to dose level (pink—0.5 × 10^6^ CAR-T cells/kg, blue—1.0 × 10^6^ CAR-T cells/kg, green—1.0 × 10^6^ CAR-T cells/kg; blue squares represent complete metabolic response determined by PET/CT scan; green squares represent partial metabolic response determined by PET/CT scan; yellow diamonds indicate refractory or persistent disease determined by PET/CT scan, light green circle represents CRS/ICANS grade ≤ 2; dark green circle represents CRS/ICANS > 2; orange triangle represents CD19-positive relapse; red triangle represents CD19-negative relapse; black cross represents death.

The ALL trial enrolled heavily pre-treated and refractory patients (*n* = 31); 42% had failed an allogeneic transplant (Table 1). In parallel, 23 relapsed/refractory adult lymphoma patients were treated. All patients and/or legal guardians provided informed consent. Twenty-seven ALL patients were evaluable for day 28 response (Fig. 3A). Among them, MRD-negative CR rate was 89% (*n* = 24). CR rate in the intent-to-treat population was 77%. In addition, all patients that did not survive for day-28 response evaluation had no evidence of leukemia on day 14 after cell infusion. Characteristics of non-responders are presented in Supplementary Table 1. Leukemia load, previous transplant or blinatumomab exposure, or LDH levels did not predict response. Allogeneic hematopoietic transplant was not used routinely as consolidation therapy. At a median follow-up of 17 months, 16 patients relapsed (CD19 negative in 8). Median time to relapse was 264 days (range, 89–696). One-year overall survival (OS) estimate (intention to treat) for the ALL cohort is 67.7% (95% CI 53.1–86.4%). For ALL patients achieving CR at day 28 (*n* = 24), one-year OS and PFS estimates are 79.2% (95% CI 64.5–97.2%) and 37.5% (95% CI 22.4–62.9%). Median duration of response for patients in CR was 310 days (10.2 months; 95% CI 8.2 months—not reached). Three patients received allogeneic hematopoietic transplant after achieving CR. All acute leukemia patients achieving CR (*n* = 24) had B cell aplasia on day 30. The proportion of patients in continued remission with persistent B cell aplasia (a secondary study endpoint) decreased over time (Supplementary Fig. 2E). One year after infusion, 67% of evaluable patients had CAR-T cell persistence (Supplementary Fig. 2D).

**Table 1:** Patient characteristics.

	Acute lymphoblastic leukemia (*n* = 31)	Non-Hodgkin lymphoma (*n* = 23)
Age, median (range), years	10 (1–20)	60 (33–76)
*Gender*, *n* *(%)*		
Female	9 (29)	8 (35)
Male	22 (71)	15 (65)
*Lymphoma subtype, n (%)*		
Diffuse large B cell lymphoma	—	9 (39)
Primary mediastinal B cell lymphoma	—	1 (4)
Transformed indolent lymphoma	—	3 (13)
Follicular lymphoma	—	3 (13)
Mantle cell lymphoma	—	6 (26)
Burkitt lymphoma	—	1 (4)
*Primary indication*, *n* *(%)*		
Primary indication	3 (10)	—
Refractory relapse	28 (90)	21 (91)
Non-refractory relapse	—	2 (9)
Previous systemic therapies, median (range)	3 (1–4)	5 (2–7)
*Previous therapy,* *n* *(%)*		
Autologous HSCT	—	10 (44)
Allogeneic HSCT	13 (42)	—
Blinatumomab	11 (35)	—
Anthracycline-containing regimen	—	22 (96)
Rituximab		23 (100)
*Bone marrow leukemia burden*, *n* *(%)*		
<5%	13 (42)	—
5–20%	5 (16)	—
>20%	13 (42)	—
Bone marrow blast, median, % (range)	9 (0–99)	—
Bone marrow CD19 expression of gated leukemia population, median % (range)	98 (35–100)	—
Bulky lymphoma (i.e. >7.5 cm longest diameter)	—	6 (26)
Elevated LDH prior to conditioning chemotherapy	—	8 (35)

NHL patients received a median number of five prior therapies (range, 2–7; Table 1); nine subjects had the primary refractory disease and 18 had disease refractory to the last treatment regimen. One subject died on day 8 after CAR19 infusion as a result of CRS associated with a high tumor burden (Fig. 3B). Among the 22 evaluable subjects, 16 patients achieved CR and two, partial response (PR). For the intention to treat the population (*n* = 23), the CR rate was 70%, and the overall response rate, 83%. Three patients did not respond, two of whom died of progressive disease, 72 and 228 days after treatment (Supplementary Table 2). Univariate analysis did not reveal predictors of response, disease status, lymphoma subtype, previous transplant, or LDH levels. Likely due to the small number of non-responders, univariate analyses did not reveal statistically significant predictors of response, including disease status, lymphoma subtype, previous autologous transplant, or baseline LDH, ferritin, and CRP levels (Supplementary Table 3).

**Fig. 4:**
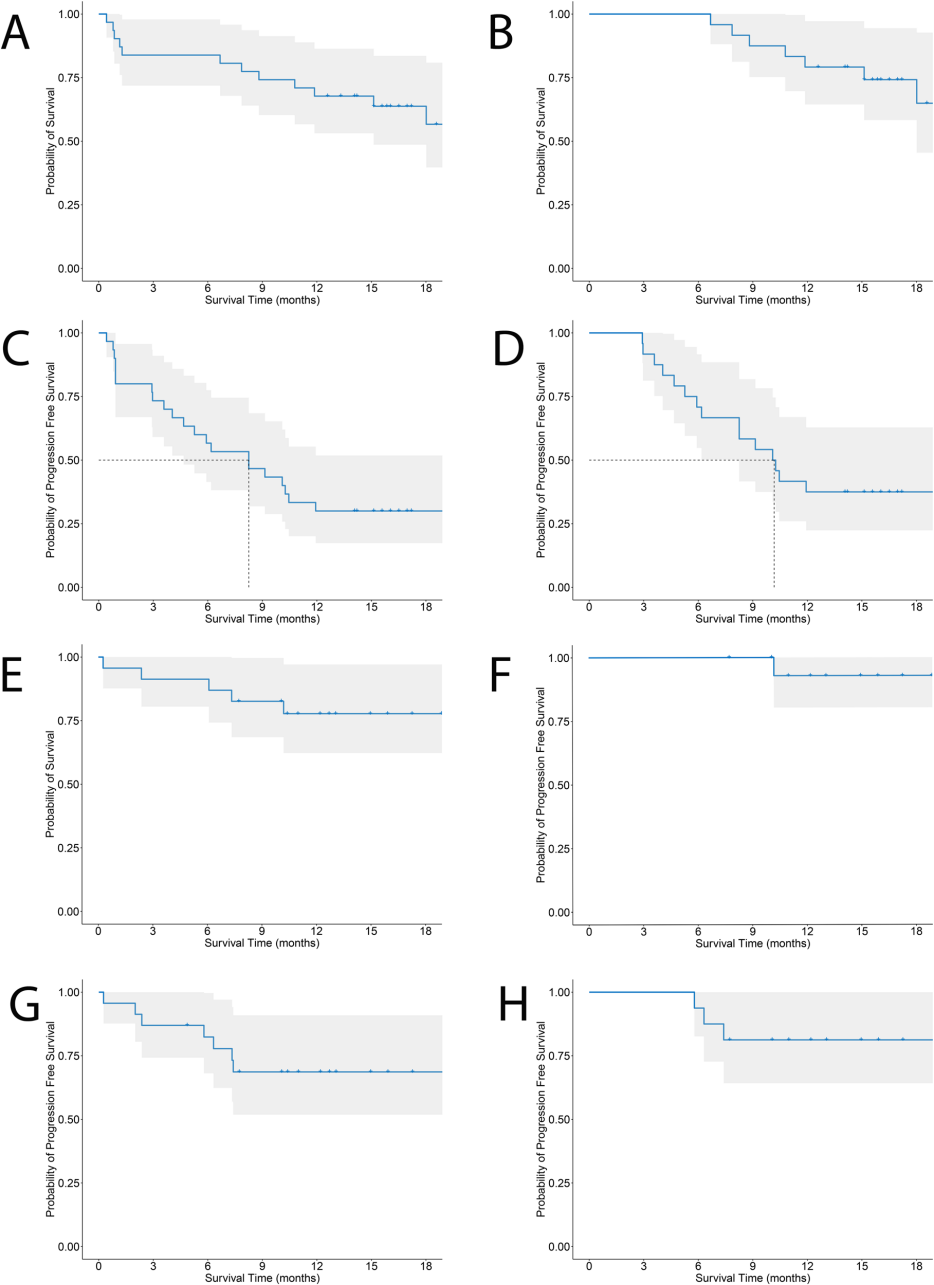
Overall and progression-free survival of leukemia and lymphoma patients. **A** Overall survival of the intention to treat ALL cohort (*n* = 31), including one patient with manufacturing failure. Gray area denotes 95% confidence interval of Kaplan–Meier survival estimate. **B** Overall survival of ALL patients achieving complete remission on day 28 assessment (*n* = 24). Gray area denotes 95% confidence interval of Kaplan–Meier survival estimate. **C** Progression-free survival of ALL patients infused CAR19 (*n* = 30). Gray area denotes 95% confidence interval of Kaplan–Meier survival estimate. **D** Progression-free survival of ALL patients achieving complete remission on day 28 assessment (*n* = 24). Gray area denotes 95% confidence interval of Kaplan–Meier survival estimate. **E** Overall survival of the lymphoma cohort (*n* = 23). Gray area denotes 95% confidence interval of Kaplan–Meier survival estimate. **F** Overall survival of lymphoma patients achieving complete remission by day 90 (*n* = 16) Gray area denotes 95% confidence interval of Kaplan–Meier survival estimate. **G** Progression-free survival of lymphoma patients infused CAR19 (*n* = 23). Gray area denotes 95% confidence interval of Kaplan–Meier survival estimate. **H** Progression-free survival of lymphoma patients achieving complete remission by day 90 (*n* = 16). Gray area denotes 95% confidence interval of Kaplan–Meier survival estimate.

After a median follow-up of 17 months (range 0–26), 6 patients have died, with a 1-year OS estimate (intention to treat) of 77.7% (95% CI 62.3–97.1%). Three CR patients have relapsed. All were asymptomatic, CD19+ and detected on surveillance imaging performed at 6 months follow-up. One subject who achieved day 30 partial imaging metabolic response progressed on day 60 imaging, with CD19-disease. One-year OS and PFS estimates for CR patients were 92.9% (95% CI 80.3–100%) and 81.2% (95% CI 64.2–100%), respectively (Fig. 4C–J). The median duration of response has not been reached (95% CI NR–NR).

### Toxicity

#### B-ALL

Prophylactic tocilizumab, 8 mg/kg intravenously 1 h prior to CAR-T cell infusion on day 0, was administered to all pediatric patients. Five patients were treated at dose level 1 (0.5 × 10^6^/kg), then 2 DLTs were seen, then five patients were treated at dose level 0 (0.1 × 10^6^/kg), five additional patients at dose level 1, 10 patients at dose level 2 (1 × 10^6^/kg), and three patients at dose level 3 (3 × 10^6^/kg), then 1 case of DLT was seen, detailed in Supplementary Fig. 3A.

Overall,19 (63%) patients had either CRS or ICANS, in 13 (68% of all CRS/ICANS) cases severity was grade 1–2. Severe (grade 3–5) adverse events occurred in six (20%) patients (Supplementary Tables 4 and 5). There were two (7%) cases of severe CRS (grade 3 *n* = 1, grade 4 *n* = 1), four (13%) cases of severe ICANS (grade 3 *n* = 2, grade 5 *n* = 2). Additionally, two cases of probable graft-versus-host disease (GVHD) reactivation and 4 cases of culture-positive sepsis were recorded. Overall, seven (23%) patients were admitted to the ICU due to CRS (*n* = 1), sepsis (*n* = 2) or ICANS (*n* = 4). The median time to first signs of CRS or ICANS was 7 (range, 1–17) days. Eighteen patients received dexamethasone, the median number of 2 doses, and 13 patients received additional tocilizumab at a median of 1 dose.

Two patients developed fatal brain edema. The first presented on day 8 after 0.5 × 10^6^/kg CAR19 in a patient with high leukemia burden and was associated with autopsy-documented multi-drug resistant *Klebsiella pneumonia* encephalitis. The second case was diagnosed on day 4 after 3 × 10^6^/kg CAR19 infusion in a patient with high leukemia burden.

Overall, 13 patients died during follow-up, 10 of them after ALL progression or relapse. Three patients died due to CRS + sepsis (*n* = 1), ICANS + sepsis (*n* = 1) or ICANS (*n* = 1) before day 28 response assessment. Overall, non-relapse mortality was 10% (3 of 31 patients) in the ALL cohort.

None of the patients with <20% bone marrow blasts developed grade 3–5 CRS/ICANS vs. seven (54%) among subgroup with >20% blasts (*p* = 0.002; Supplementary Table 4). There was also a significant correlation between severe CRS and ICANS with LDH levels (Supplementary Fig. 4).

#### NHL

Prophylactic tocilizumab was instituted in the NHL trial after May 2019, informed by the Russian trial, administered at a dose of 8 mg/kg, 1 h prior to CAR-T cell infusion.

Three patients were initially treated at dose level 1 (0.5 × 10^6^ CAR T cells/kg) without dose-limiting toxicities; one patient treated in dose level 2 (1 × 10^6^ CAR T cells/kg) had lethal CRS, resulting in expansion of that dose level to a total of six patients initially. This dose level was expanded further to an additional 10 patients after CAR T manufacturing was shortened from 12 to 8 days. No additional dose-limiting toxicities were observed at this dose level or at the target dose of 2 × 10^6^ CAR T cells/kg (Supplementary Fig. 3B). During the expansion phase of dose level 2, one subject with high tumor burden and elevated baseline inflammatory markers was administered a reduced dose of 0.5 × 10^6^ cells/kg.

Adverse events included CRS in 12 patients (Supplementary Table 4). The median time to CRS was 4 days (range, 1–7). CRS was graded 1–2 (*n* = 11), while one patient died of severe CRS in the context of bulky disease after 1 × 10^6^/kg CAR19 cells. Nine patients required tocilizumab for the treatment of grade ≥2 CRS (5 of whom had had prophylactic tocilizumab), while steroids were used for the treatment of persistent grade 2 CRS in two patients. Five NHL subjects developed grade 1–2 (*n* = 3) or grade 3–4 (*n* = 2) ICANS at a median of 7 days (range, 4–12). All events resolved with corticosteroids, and there were no instances of brain edema. All NHL patients had grade 3–4 neutropenia. Recurrent neutropenia after day 30 was observed in 6 NHL subjects. Anemia was observed in all patients, (grade 3–4, *n* = 15), and thrombocytopenia in 21 patients (grade 3–4, *n* = 13). The most common non–hematologic toxicity was grade 1–2 fatigue, (*n* = 14). There were no grade 3–4 non-hematologic toxicities other than CRS or ICANS.

Five patients died in the follow-up period. Four patients died after relapse or progression of NHL. One patient died 8 days after CAR T cell infusion after experiencing severe CRS (Supplementary Table 6).

In NHL patients, prophylactic tocilizumab resulted in higher plasma concentrations of IL-6 on days 2 and 6 after CAR-T cell infusion, although the difference compared to patients treated without prophylaxis did not reach statistical significance (Supplementary Fig. 5).

Analysis of infused CD19 CAR T cell products show memory CD8+ TCF7+ cells were associated with CR and a transitional memory-like phenotype was associated with the absence of severe CRS

**Fig. 5:**
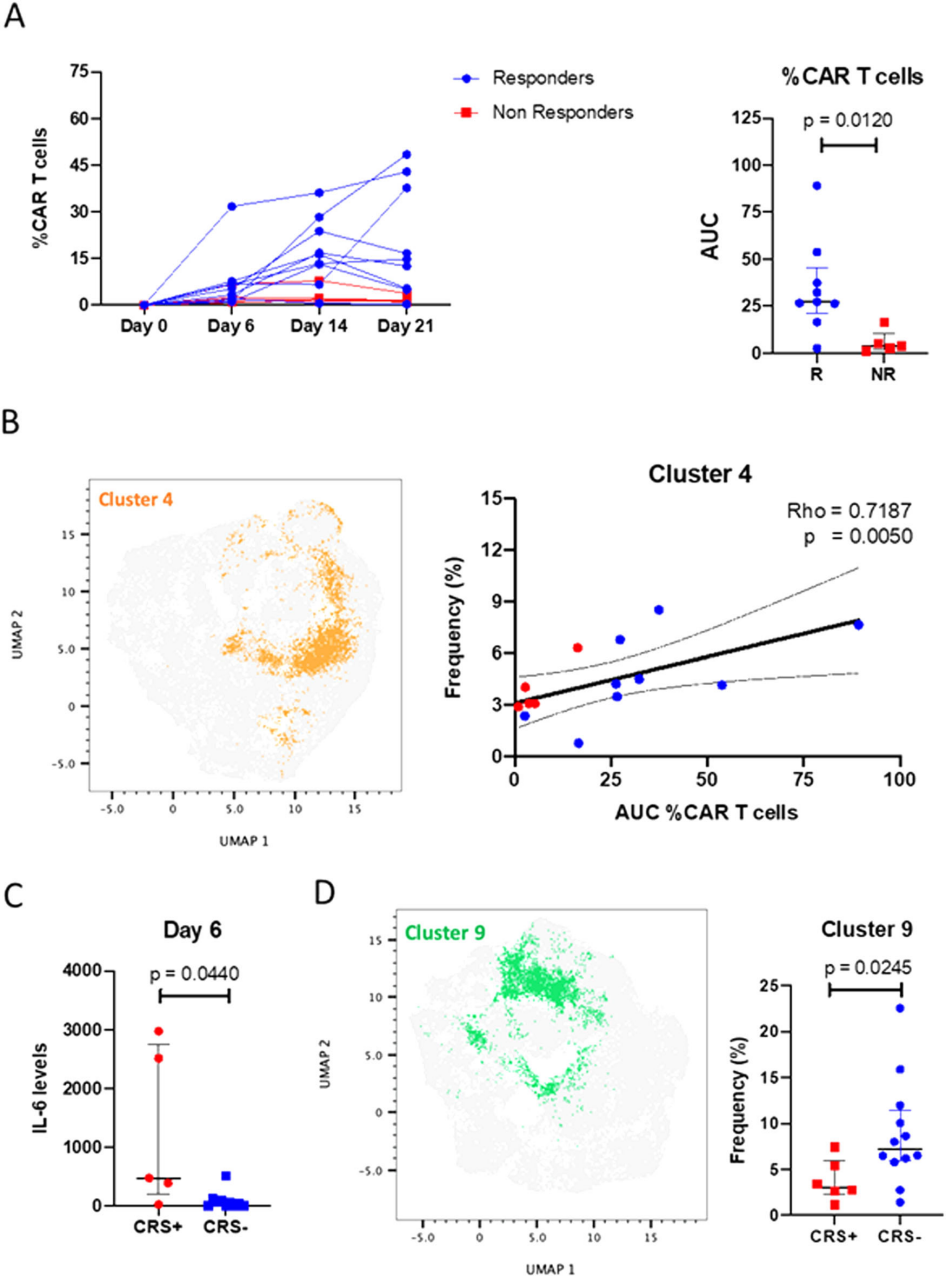
Responders have a higher frequency of CD19 CAR T cells and memory-like CD8+ CD19 CAR T cells. Patients with CRS have higher IL-6 levels and transitional memory-like CD4+ CD19 CAR T cells. **A** Left: Line graph showing the percentage of CAR T cells in participants until day 21. Each line represents a single patient. Right: Statistically significant difference of percentage of CAR T (area under the curve—AUC) between responders (*n* = 12) and non-responders (*n* = 5) (*p* < 0.05). Each dot represents the AUC of a single individual and lines represent the interquartile range. Blue lines and dots represent responders and red dots and lines represent non-responders. **B** Right: UMAP showing the distribution of cluster 4 in CD8+ CAR+ T cells. Left: Scatter plot showing significant positive correlation (Rho = 0.7187, *p* < 0.05) of frequency of cluster 4 (memory-like phenotype) in CD8+ CAR+ T cells and % CAR T cells in participants. Blue dots represent responders and red dots represent non-responders. **C** IL-6 levels were significantly higher (*p* < 0.05) in patients that present CRS vs. patients that did not present CRS. Each dot represents the Il_6 levels of a single individual and lines represent the interquartile range. Red dots represent patients with CRS, blue dots represent patients without CRS. **D** Left: UMAP showing the distribution of cluster 9 in CD4+ CAR+ T cells. Right: Frequency of transitional memory-like CD4+ CAR+ T cells (cluster 9) was higher (*p* < 0.05) in patients with grade 1 or no CRS compared to patients with CRS grade 2 or higher. Red dots represent patients with CRS, blue dots represent patients without CRS. Each dot represents the frequency of Cluster 9 CAR-T cells in a single individual and lines represent the interquartile range. Statistical significance was assessed by performing Mann–Whitney *U* test. Source data are provided as a Source Data File for panels **A**–**D**.

To identify correlates of clinical outcomes, the lymphoma cohort was divided into two groups. Responders (R; *n* = 12) included patients who had achieved CR and non-responders (NR; *n* = 5) included participants with PR, progressive or stable disease. Comparison of the Area Under the Curve of percentages of CAR+ T cells in patients at days 6, 14, and 21 showed that responders had a significantly higher percentage (*p* = 0.0120) of CAR19 T cells (Fig. 5A).

**Fig. 6:**
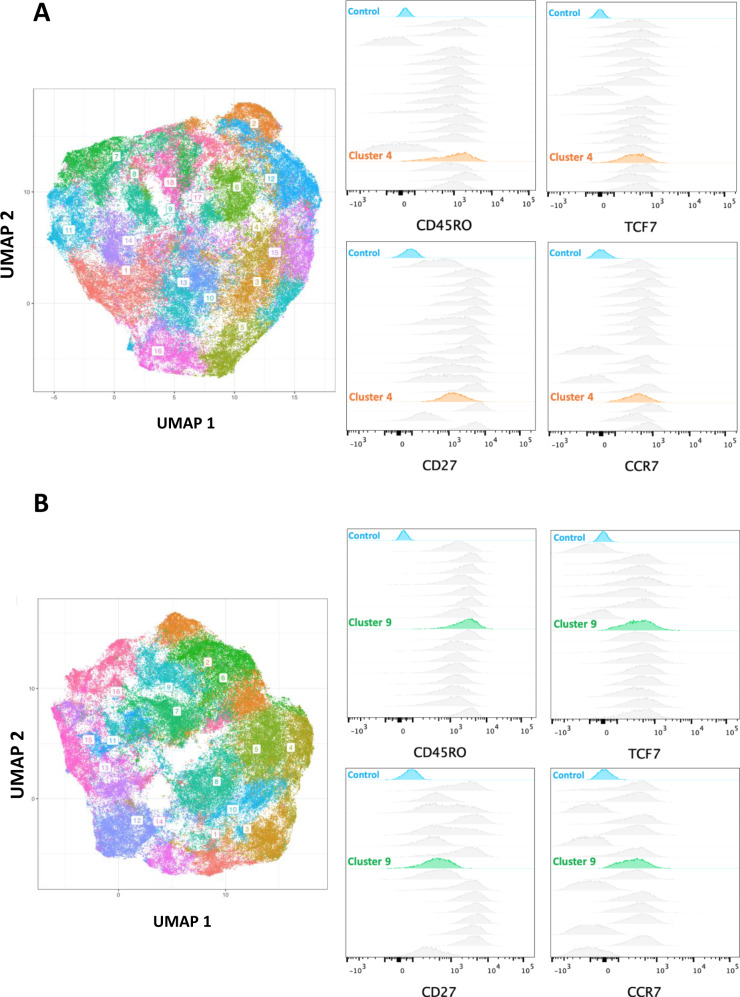
Phenotypic characterization of clusters associated with outcome and toxicity. **A** Right: UMAP showing the distribution of all clusters in CD8+ CAR+ T cells. Left: Phenotypic characterization of cluster 4 shows an expression of CD45RO, TCF7, CD27, and CCR7 in comparison with other clusters and with control (blue). **B** Right: UMAP showing the distribution of cluster among clusters in CD4+ CAR+ T cells. Left: Phenotypic characterization of cluster 9 shows an expression of CD45RO, TCF7, and CCR7, and lack of expression of CD27 in comparison with other clusters and with control (blue).

We performed unsupervised clustering analysis of cell surface phenotypes to identify a unique population of CD8+ T cells in the CAR19 cells compartment associated with response. Out of 18 clusters evaluated, only cluster 4 significantly positively correlated with percentages of CAR19 T cells (rho = 0.7187, *p* = 0.0050; Fig. 5B). Phenotypic characterization of cluster 4 (Fig. 6A) showed that this cluster exhibited upregulation of memory markers CD45RO, CD27 and CCR7, and TCF7, a transcription factor that has been shown to be expressed by long-lived T cells^23^, indicating that these cells share a phenotype of memory T cell stem^24,25^. Additionally, these cells showed low expression of CD127, 41BB, and did not express Ki67.

Next, surrogate markers of CRS were evaluated. Patients were divided into two groups: CRS+ (*n* = 6, with grade 2 or higher CRS), and CRS− (*n* = 11, with grade 1 or no CRS). As Interleukin 6 (IL-6) plays a central role in acute inflammation^26^, IL-6 plasma levels were evaluated on day 6. As expected, patients with CRS showed significantly elevated levels of IL-6 (Fig. 5C). There was no correlation of CD8 T cell clusters with day 6 IL-6. However, cluster 9 in the CD4+ CAR19 T cell compartment was significantly lower (*p* = 0.0245) in the CRS+ group (Fig. 5D). This cluster (Fig. 6B) showed upregulation of memory markers CD45RO and CCR7, TCF7, the marker of T cell stemness, CD127, the IL-7 receptor also highly expressed by memory T cell stem cells and other long-lived cells and the proliferation marker Ki67. Therefore, cluster 9 CD4+ cells are present at higher frequencies in patients with minimal or no CRS.

## Discussion

We describe successful place-of-care manufacturing of CAR19 T cells in two disparate clinical sites. Severe toxicity occurred in association with high disease volume, reflecting the advanced disease status of patients treated here^19,20,27^. Response rates were at least comparable to published experience. The role of prophylactic tocilizumab in this setting is under investigation.

Place-of-care offers several advantages over centralized manufacturing, including reduced vein-to-vein time due to lack of transport to a centralized facility and the ability to infuse fresh and not necessarily cryopreserved products. Simplified logistics increase flexibility to make decisions based upon patient disease status, for example, split-dosing in the instance of a high tumor burden. Interestingly, fresh cells provided faster tumor control in animals, as compared to cryopreserved cells.

We used the GMP-compliant CliniMACS Prodigy^®^ closed-cell manufacturing system with associated materials and reagents. The device, materials, and reagents used between the two clinical sites were identical. Despite the distinct nature of the apheresis product (pediatric vs. adult), transduction efficiency and cellular composition were consistent between the two sites. Moreover, the median apheresis to infusion time was 13 days, a shorter interval than that reported by others^20^. This time may be further reduced to 8 days^22^. As a result of the shorter apheresis to infusion time, most enrolled patients received CAR19.

Patients that achieved CR had a higher frequency of long-lived memory CD8+ TCF7+ cells. Interestingly, others have associated this cell subtype with positive outcomes after CAR T and other cancer treatments, such as melanoma^28,29^. We, therefore, identified product cell subsets correlated to tumor control and CRS, and enriching for these subpopulations may improve clinical outcomes.

CAR19 was highly effective in inducing disease response. Most of our ALL patients became MRD-negative. However, relapse was the main cause of treatment failure. The targeting of multiple antigens may be necessary to address the issue of antigen escape^30,31^. Post CAR T cell allogeneic transplant is also under investigation. Our approach increases the likelihood of receiving CAR therapy, but patients that would not be eligible due to disease progression while waiting for product manufacture may also be more likely to suffer toxicity or infections. However, brain edema has not been reported in patients treated with 4-1-BB CAR-T in pediatric ALL, and it is unclear if prophylactic tocilizumab was a contributing factor in severe ICANS in that subgroup. Others have used pre-emptive or prophylactic tocilizumab without unexpected side effects^32,33^. Toxicity mitigation strategies now under investigation for patients with large leukemia volume include CAR19 split dosing and use of 0.5 × 10^6^ CAR19/kg for NHL patients with bulky disease. The ELIANA study that led to the FDA approval of Tisagenlecleucel to treat pediatric ALL reported that 4.4% (*n* = 3) of patients had fatal infections, with a 12-month relapse-free survival of 59% (95% CI 41–73)^12^. In a recent registry retrospective analysis, 23% of ALL patients treated with Tisagenlecleucel developed clinically significant infections^34^.

Lymphoma responses were sustained. We observed a relapse-free survival (RFS) of 81.2% in the NHL cohort, which should be compared to larger series of patients treated with anti-CD19 CAR T cells that reported one-year RFS of 65% and 79%^35,36^.

Our studies show that place-of-care manufacture of CAR-T cells results in a consistent cell product and effective clinical outcomes, despite being manufactured in two disparate clinical centers. We, therefore, conclude that place-of-care manufacture of CAR-T cells is a valid paradigm for the manufacture and distribution of CAR-T cells among multiple clinical sites, particularly for patients with rapidly progressive, symptomatic lymphoma and ALL^12,19,20^.

## Methods

### Design of the chimeric antigen receptor targeting CD19

Lentiviral vectors encoding CAR19 or CAR19.8 were manufactured, and human primary T cells were transduced with each lentiviral vector. CAR expression was measured on the surface of transduced T cells by flow cytometry (Fig. 1B). Cells were stained with CD19-Fc reagent, followed by anti-Fc F(ab)′2-AF 647 conjugate. Non-transduced T cells maintained under the same conditions as CAR-transduced cells were used as a negative control. Robust expression of both CAR19 and CAR19.8 constructs was reproducibly achieved in three separate transduction experiments using different T cell donors (Fig. 1B, one representative donor is shown).

### Flow cytometric analysis of CAR surface expression

For cell staining, half a million CAR T-transduced cells were harvested from culture, washed two times in cold AutoMACS buffer supplemented with 0.5% bovine serum albumin (Miltenyi Biotec), and CAR surface expression detected by staining with CD19-Fc peptide (R&D, Minneapolis, MN) followed by anti-Fc-AF647 conjugate (Jackson Immuno Research, West Grove, PA). Non-transduced cells were used as negative controls. Dead cells in all studies were excluded by 7AAD staining (BD Biosciences, San Jose, CA, USA). Cells were washed twice and resuspended in 200 μL Staining Buffer before quantitative analysis by flow cytometry. Flow cytometric analysis was performed on a MACSQuant^®^10 Analyzer (Miltenyi Biotec), and data plots were generated using FlowJo software (Ashland, OR, USA).

### Functional assays

Effector cell function was tested using standard ^51^Cr release assays as previously described^37^ and used targets expressing CD19+ cancer cell lines Raji (NHL), Reh (ALL), or with CD19− control lines K562 or 293T (all cell lines obtained from ATCC). In the most recent experiments, autologous T-cell blasts were added as a negative control.

Effector function of CAR T cells was evaluated based on the production of IFN-γ, TNF-α, and IL-2 after co-culture with Raji cells by ELISA (10:1 E:T) assays as described below. Supernatants were tested for IFN-γ by ELISA using solid-phase bound anti-IFN-γ, anti-TNF-α, and anti-IL-2 capture antibody (1 μg/mL) and detection by the biotinylated anti-IFN-γ, anti-TNF-α, and anti-IL-2 detection antibody (0.5 μg/mL; both BD Biosciences). The reaction product was visualized by a peroxidase/streptavidin conjugate (1:10,000) and ABTS (Roche Diagnostics).

This study was carried out in compliance with the applicable laws, regulations, and guidelines of the National Institutes of Health (NIH) and with the approval of Covance Preclinical Oncology (formerly MI Bioresearch) Animal Care and Use Committee. The function of CD19-targeting CAR T cells was assessed in vivo. Six to eight-week-old female NOD.*Cg-Prkdc*^*scid*^*Il2rg*^*tm1Wjl*^*/*SzJ (NSG) mice, 6 per group, were injected i.v. with Raji-luc tumor cells at 5 × 10^5^ Raji/mouse cells on day 0. Tumor burden was determined by in vivo imaging system (IVIS) bioluminescent imaging on day 4, mice were then randomized to groups with equal mean tumor burden, and 5.0 × 10^6^ fresh or frozen CAR T+ cells/mouse (normalized for transduction efficiency) were administered on study day 7. Tumor regression was determined by bioluminescent imaging on days 11, 13, 21, and 28 using a Xenogen IVIS-200 instrument (Perkin Elmer, Shelton, Connecticut). Images were analyzed using Living Image, version 4.1, software (Perkin Elmer), and the bioluminescent signal flux for each mouse was expressed as average radiance (photons per second per cm^2^ per steradian). To test for possible outliers, mean radiance tumor burden values per animal across time points in each group were compared (*n* = 5 animals/group). In group CAR19 frozen high dose, mean radiance for one animal was determined to be a significant outlier using Grubbs’ test, with significance level *P* < 0.05 (two-sided), using the GraphPad platform (https://www.graphpad.com/quickcalcs/Grubbs1.cfm). Subsequently, the outlier animal was excluded from the graph and the statistical analysis.

### Manufacturing CAR T cells using the Automated CliniMACS Prodigy^®^ platform

Pre-clinical studies were performed at the Research and Development Laboratory at Lentigen. CAR T cells were manufactured in the CliniMACS Prodigy^®^ (Miltenyi Biotec) device as previously described^37,38^ at either the Dmitriy Rogachev Center, Moscow, or University Hospitals Seidman Cancer Center, Cleveland. The CliniMACS Prodigy^®^ (Miltenyi Biotec, Bergisch Gladbach, Germany) device was programmed using the TCT software program. TS520 tubing set was used for manufacture (Miltenyi Biotec, Bergisch Gladbach, Germany). Clinical-grade reagents used to manufacture the CAR-T cells included: CliniMACS Buffer, TexMACS Media, CliniMACS CD4 reagent, CliniMACS CD8 reagent, TransAct, and the cytokines IL-7 and IL-15 (Miltenyi Biotec, Bergisch Gladbach, Germany). The reagents were used according to the manufacturer’s instructions. One 25 μg vial each of IL-7 and IL-15 was added per 2 L bag of media. A 25% stock solution of Human Serum Albumin (HSA) was used to supplement the CliniMACS Buffer to a concentration of 0.5%. Human AB serum was used to supplement the TexMACS Media to a concentration of 3% and was from Innovative Research (Novi, MI, USA). TexMACS media was supplemented with Human AB serum for 6 days of the cell culture and then replaced with media without Human AB serum for the duration of the culture. The CliniMACS Prodigy was loaded with ~1 × 10^8^ CD3 cells and the cells expanded with anti-CD3/CD28 co-stimulation and IL-7/IL-15 supplementation. Following the manufacture protocol, CAR-T cells were harvested initially on day 12 of culture, and after a subsequent protocol modification, harvest on day 8 of culture was permitted.

### Analysis of CD19 CAR T cell infusion products

Immune-phenotypic characterization of CAR-T products was performed by Flow Cytometry, using the BD FACSymphony^TM^ Flow Cytometer. Antibodies used in the panels tested included: Viability stain dye (Invitrogen), CD3 BV570 (Biolegend), CD4 BUV563 (BD), CD8 BUV496 (BD), CD19CAR FITC (Acro Biosystem), CD45RO BUV395 (BD), CD27 BV421 (BD), CCR7 BUV805 (BD), 41BB BV650 (BD), TCF7 PE (BD) and Ki67 PE-Cy5 (ThermoFisher). Flow Cytometry data were analyzed by FlowJo v. 10.7. The gating strategy consisted of a set of steps starting from lymphocytes, based on SSC-A and FSC-A; single cells, based on FCS-H and FSC-A; and live cells, based on MFI for Viability dye. Consecutively specific gating was done in each population of interest e.g. CD4+ T cells, CD8+ T cells, etc. Lastly, populations of interest were concatenated and exported for unbiased clustering analysis, which was done using the Rphenograph package (https://github.com/jacoblevine/PhenoGraph). Projection of the density of cells expressing markers evaluated (above mentioned) was visualized using a non-linear dimensionality reduction technique, Uniform Manifold Approximation, and Projection (UMAP). All correlations were done using Spearman correlation. All statistical comparisons were done using the Mann–Whitney *U* test. Statistical analysis was performed using Graph Pad Prism version 8.

### Clinical trial design and toxicity monitoring

Two separate clinical trials are discussed here. The trials were registered at clinicaltrials.gov under NCT03467256 (ALL) and NCT03434769 (NHL). The pediatric ALL study was approved by the Institutional Review Board of the Dmitriy Rogachev National Medical Center of pediatric hematology and the adult Non-Hodgkin Lymphoma Study was approved by the Institutional Review Board of University Hospitals Cleveland Medical Center. All human subject research was conducted according to the criteria set by the Declaration of Helsinki, and all participants or their legal guardians provided written informed consent. Thirty-one pediatric patients with ALL were enrolled between February 2018 and May 2019. Twenty-three lymphoma patients were enrolled between July 2018 and March 2020.

ALL patients were treated at four dose levels (0.1 × 10^6^, 0.5 × 10^6^, 1 × 10^6^ and 3 × 10^6^ CAR19 cells/kg) (Supplementary Fig. 3A). NHL patients received 0.5 × 10^6^ (*n* = 4), 1 × 10^6^ (*n* = 16), or 2 × 10^6^ (*n* = 3) CAR19 cells/kg (Supplementary Fig. 3B).

Dose-limiting toxicity (DLT) in the ALL trial was defined as grade 4 and 5 SAE, excluding sepsis/septic shock in patients with bone marrow aplasia. Cytokine release syndrome (CRS) and immune effector cell-associated neurotoxicity syndrome (ICANS) were graded and managed as previously described^39^. In the NHL study, DLT has defined as grade ≥3 nonhematologic toxicities and grade ≥4 hematologic toxicities of more than 21-day duration, graded per NCI Common Terminology Criteria for Adverse Events (CTCAE) version 5.0 and grade. Exceptions to the DLT definition included laboratory abnormalities without symptoms and resolved within 7 days, grade 2 or lower CRS, grade 3 CRS that improved to grade 2 within 3 days, and grade 3 or lower ICANS that improved to grade 1 within 3 days.

Possible neurotoxic effects were evaluated daily during an inpatient stay and in all outpatient visits. At baseline and then at approximately 14 and 21 days post-infusion, the caregiver of the patient or the adult patient also completed a neuro-symptom checklist^40^.

### Eligibility

Eligibility criteria are summarized at clinicaltrials.gov and the full list is available in the study protocols (provided as Supplementary Notes 1, 2 within the Supplementary Information file). Patients were eligible if their disease had recurred after standard upfront therapy and at least one (ALL) or two salvage therapies (NHL).

### Response monitoring

ALL patients had a baseline bone marrow aspiration and lumbar puncture performed within 14 days before the lymphodepletion preparative regimen. Bone marrow biopsy and lumbar puncture were performed 28 ± 4 days from cell infusion, and after 2, 3, 6, 9, and 12 months. Complete remission (CR) was defined by morphologic assessment of the bone marrow (<5% blasts) with no extramedullary disease. Measurable residual disease (MRD) was assessed by multiparametric flow cytometry with a sensitivity of 10^−4^. NHL disease status and the response were assessed by PET scans performed prior to, and at 1, 2, 6, and 12 months after CAR19.

### Lymphodepleting chemotherapy

ALL patients received fludarabine 30 mg/m^2^ daily for 4 days and cyclophosphamide 750 mg/m^2^, while NHL patients received fludarabine 25 mg/m^2^ daily for 3 days and cyclophosphamide 60 mg/kg. The choice of a higher dose of cyclophosphamide was used based on original studies by Turtle and colleagues^15^. Given that patients were not allowed to receive bridging chemotherapy due to the rapid manufacture time, a larger dose was considered also necessary for disease control.

### Statistical methods

Univariate analyses of patient characteristics used Fisher’s exact test for categorical variables and Wilcoxon signed-rank test for continuous variables. Overall survival (OS), progression-free survival (PFS), and duration of response estimates were done using the Kaplan–Meier method.

For studies of the infused cell product, unbiased clustering analysis was done using the Rphenograph package (https://github.com/jacoblevine/PhenoGraph). Projection of the density of cells expressing specific markers was visualized using a non-linear dimensionality reduction technique: uniform manifold approximation and projection (UMAP). All correlations of cell phenotype were done using Spearman correlation and statistical comparisons were done using Mann–Whitney *U* test. Statistical analysis was performed using Graph Pad Prism version 8 and R and its packages (http://cran.r-project.org).

### Reporting summary

Further information on research design is available in the Nature Research Reporting Summary linked to this article.

## Supplementary information

Supplementary Information

Reporting Summary

## Data Availability

Respective study protocols of the two clinical trials are available as Supplementary Notes 1-2 in the Supplementary Information File. Any requests for additional data, materials, or clinical data will be reviewed by each of the Institutional Review Boards and will require a Material Transfer Agreement in place. Patient-related data not included in the paper were generated as part of a clinical trial and are subject to patient confidentiality. All data shared will be de-identified. Any requests for further data should be addressed to the corresponding authors (marcos.delima@osumc.edu or boro.dropulic@caringcross.org). The remaining data are available within the Article, Supplementary Information or Source Data File. Source data are provided with this paper.

## Source data

Source Data
