# Supplementary material for: Multiple site place-of-care manufactured anti-CD19 CAR-T cells induce high remission rates in B-cell malignancy patients
Source: Nat Commun. 2021 Dec 10;12:7200. doi: 10.1038/s41467-021-27312-6 (PMC8664838; doi:10.1038/s41467-021-27312-6)
Supplement: Supplementary file 1 — Supplementary Information [file 41467_2021_27312_MOESM1_ESM.pdf]

Supplementary Figure 1

```
CD8:        CDIYIWAPLAGTCGV-LLLSLVILYCKR
CD4:        PM-AL-IVLGGVAGLLLFIGLGIFFCVRC
TNFRSF16:   TTDNL-IPVYCSILAAVVVGLVAYIAFKR
TNFRSF19:   TA--L-AAVICSALATVLLALLILCVIYC
TNFRSF9:   IISFFL-ALTSTALL--FLLFFLTLRFSVV
CD28:        FWVLVVVGGVLACYS-LLVTVAFIIFW
```

Supplementary Figure 1. CAR19. Homology search results were annotated to reflect known lymphocyte cell surface proteins, and although sequence diversity was greater than anticipated, motifs in common (highlighted) could be identified in TNFRSF9 (CD137), TNFRSF16 (CD271, NGFR, or commonly LNGFR) and TNFRSF19 (TROY). CD4 and CD8 are included for comparison.

Supplementary Figure 2

A

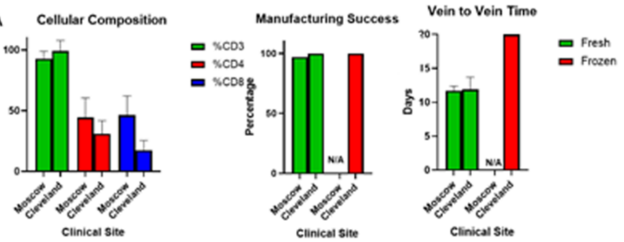

B

	ALL Patients	Lymphoma Patients
Patients Treated (%)	96	100
Median CAR T Transduction (%)	60	48
Median Apheresis to Transfusion Time (days)	13	13

C

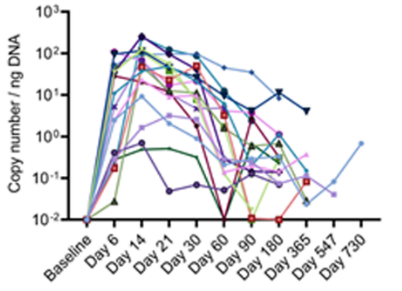

D

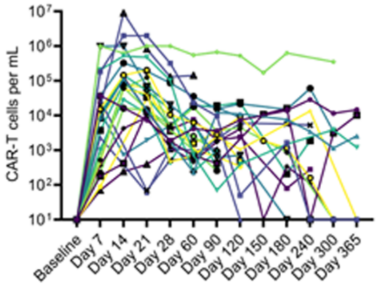

E

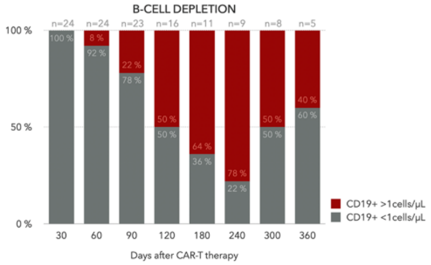

Supplementary Figure 2: Local manufacture of CD19 CAR T cells for ALL (n = 31) and Lymphoma patient cohorts (n = 23). (A) Cell composition (left), manufacturing success (middle), and vein to vein time (right) for patients in Moscow and Cleveland cohorts. Mean values presented. Error bars represent SD (B) Local manufacture of CD19 CAR T cells has a low apheresis to infusion time. (C) CAR T cell persistence in NHL patients (D) CAR T cell persistence in B-ALL patients. (E) B cell aplasia in ALL patients represented as percentage of total evaluable patients. Source data are provided as a Source Data File.

Supplementary Figure 3, panel A

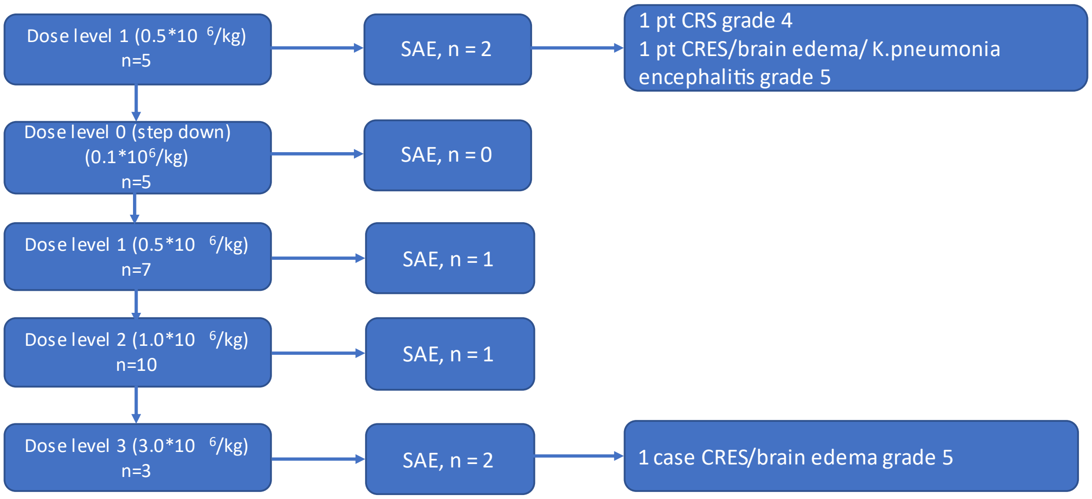

Supplementary Figure 3, panel B

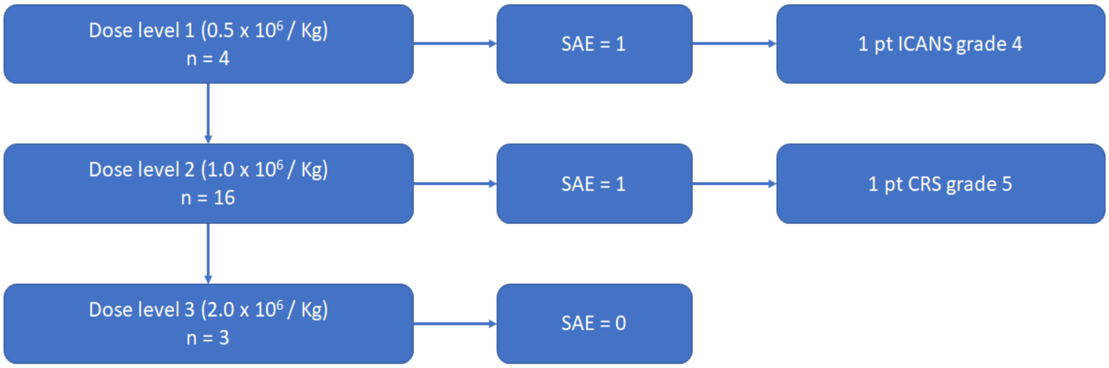

Supplementary Figure 3. Panel A. Schematic representation of CAR19 dosing and toxicity in acute lymphocytic leukemia. Dose level decisions as a function of toxicity are depicted here. The first instance of cerebral edema in dose level one led to a dose decrease (dose level zero), and the second case of cerebral edema (at dose level 3) was included in the ruling to recommend dose level two for phase II study. Pt, patient; SAE, severe adverse event; ICANS, Immune effector cell-associated neurotoxicity syndrome. Overall, 37 patients were screened, 31 enrolled and 30 patients received CAR-T infusion.

This was the first clinical trial of CAR-T cell therapy ever done in Russia. At study start the national regulator did not specify a particular design for early phase adoptive cell therapy trial and N=5 per dose level was accepted. A classic 3x3 design was not used due to the fact that dose-toxicity for adoptive cell therapy is not linear and is dependent on additional factors, such as tumor load. Based on this sample size was increased to account for potential variability of the population (such as leukemia burden and previous transplant). Initial patient enrollment plan was to enroll 5 patients per dose level with a 0 dose level (100k/kg) serving as a de-escalation step. As reported, 2 SAE were registered at dose level 1 (500k/kg): 1 grade 4 CRS, which resolved, but the patient succumbed later to culture-proven Gram-negative BSI/sepsis. The second SAE was Grade 5 ICANS (brain edema). These two SAE developed within 3 weeks and triggered DSMB meeting. Grade 4 CRS was not considered a DLT and death due to sepsis was not considered DLT per protocol (since multiple preexisting risk factors for sepsis, unrelated to the CAR-T therapy, were expected in the patient population). DSMB rule was to step-down to dose level 0. Upon completion of dose level 0 enrollment an autopsy from grade 5 ICANS was available, demonstrating K. pneumonia encephalitis, culture and NGS-proven. Since formal DLT was not reached, reescalation was allowed by DSMB. Upon re-escalation no DLT was registered at dose levels 1 (500k/kg) (7 pts) and 2 (1m/kg) (10 pts). At 3m/kg (dose level 3) 2 SAE, one DLT (grade 5 ICANS) were registered, and enrollment closed. **Panel B**. Dose escalation schema and immune effector cell associated-toxicities in the NHL cohort. Dose level 1 (0.5 x 10^6/Kg) included 3 subjects in dose escalation and one subject with bulky disease that had per-protocol CAR-T cell dose reduction. This subject experienced ICANS grade 4 that resolved promptly with steroid therapy. Dose level 2 (1 x 10^6 cells/Kg) was expanded after one death secondary to CRS was observed. Further expansion of this dose level was done to evaluate changes in CAR-T manufacturing process. Overall, 26 patients were enrolled and 23 were treated (two patients decided to withdraw from the study and one patient was in remission and therefore not eligible).

The adult NHL study followed a 3+3 dose escalation; patients with bulky disease (defined as any mass larger than 10cm) were treated at the dose level below dose escalation. Dose expansion of up to 10 subjects in any dose level was permitted beyond the 3+3 schema. The cohort of 1x10^6 CAR-T cells/Kg was first expanded to 6 subjects after one patient presented fatal CRS, and then additional enrollment in that cohort was done to assess safety after manufacture change from 12 to 8 days (n = 3). Additional subjects to that dose level was permitted under dose expansion rules.

Supplementary Figure 4

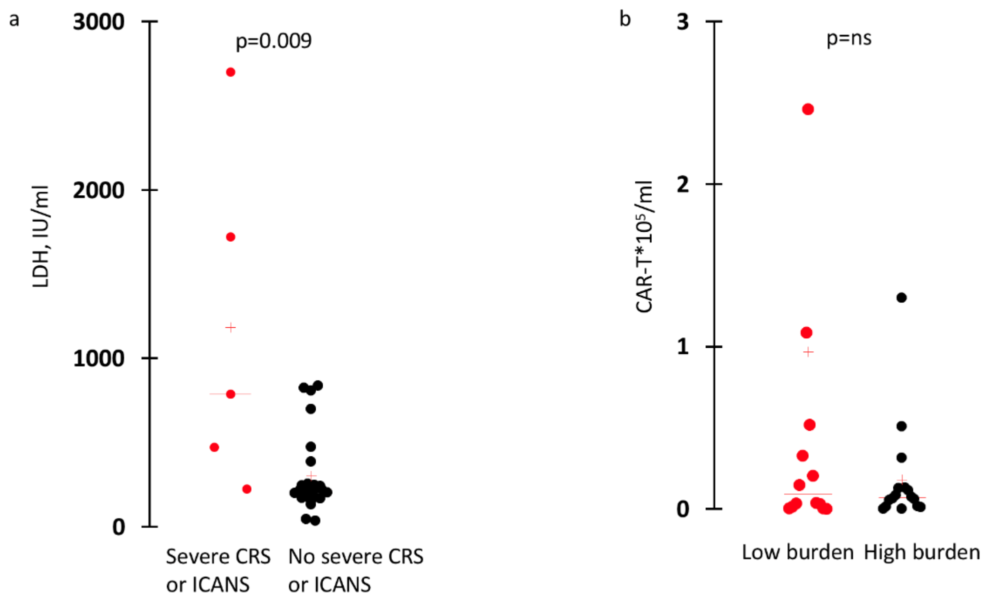

Supplementary Figure 4. LDH levels before lymphodepletion correlated positively with the composite severe CRS+ICANS outcome but not any CRS+ICANS in the ALL cohort (left panel). Comparison of LDH level before lymphodepletion between the groups of pediatric ALL with (n=5) or without (n=25) grade 3-5 CAR-T-related adverse events (CRS and ICANS). Comparison of peak CAR-T expansion between the groups of pediatric ALL with high (n=16) or low (n=14) leukemia burden. Horizontal lines represent median, cross represents mean. For both comparisons, Mann-Whitney U test. Source data are provided as a Source Data File.

Supplementary Figure 5

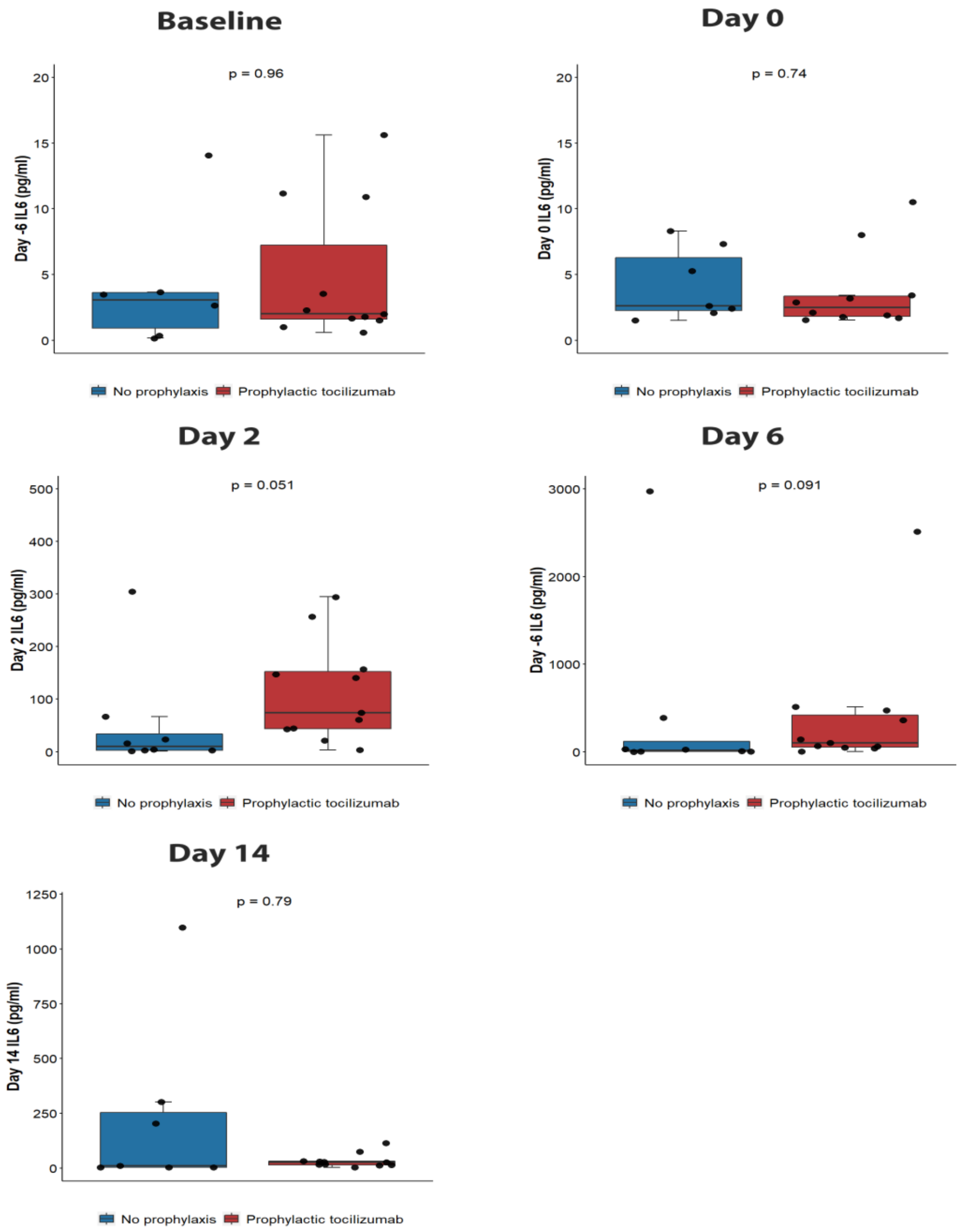

Supplementary Figure 5. IL-6 levels in lymphoma patients that received (n = 11) versus patients who did not receive prophylactic tocilizumab (n = 8). Minima: 1st quartile; maxima: 3rd quartile: centre: median; bounds of box: interquartile range; bounds of whiskers: minimum and maximum values, excluding outliers. Dots represent individual observations. All statistical comparisons were done using two sided Mann-Whitney U test without corrections for multiple comparisons. Source data are provided as a Source Data File.

Supplementary Tables

Supplementary Table 1: Characteristics of three non-responders in the ALL patient cohort.

Age	Indication	Previous HSCT	Previous blinatumomab	CD19 expression	Blast count	CAR-T dose
20	Refractory relapse	Yes	Yes	100%	100	1×10^6
10	Refractory relapse	Yes	Yes	100%	0	0.1×10^6
12	Refractory relapse	No	No	35%	95	0.1×10^6

Supplementary Table 2: Characteristics of three non-responders in the lymphoma patient cohort.

Age	Diagnosis	Primary refractory	Previous lines of therapy	Previous autologous HSCT	Refractory to last line of therapy	Bulky disease	CAR-T dose
63	Transformed indolent	No	6	No	Yes	Yes	0.5×10^6/kg
60	Transformed indolent	Yes	4	Yes	Yes	No	1.0×10^6/kg
44	DLBCL	Yes	3	No	Yes	Yes	1.0×10^6/kg

Supplementary Table 3: Univariate analysis comparing responders versus non-responders in the lymphoma cohort.

	**Response (n = 18)**	**No response (n = 5)**	**p value***
CAR-T cell dose			
0.5 x 10^6 cells/kg	3	1	0.78
1.0 x 10^6 cells/kg	13	3	
2.0 x 10^6 cells/kg	2	1	
Bulky disease	3	3	0.14
Symptomatic disease*	12	4	0.51
Primary refractory disease	5	4	0.10
Refractory to last line of therapy	16	5	1.00
Prior autologous transplant	8	2	0.80
Baseline* LDH, U/L, median	267	1676	0.07
Baseline* Ferritin, mg/dL, median	190	497	0.25
Baseline* CRP, mg/dL, median	0.9	4.0	0.17
* at the time of lymphocyte collection			

* Fisher's exact test for categorical variables and Wilcoxon signed rank test for continuous variables.

Supplementary Table 4: Acute lymphocytic leukemia cohort. Increased bone marrow blasts was associated with severe toxicity.

	**Patients enrolled, n=31**	**>5% BM blast, n=19**	**< 5% BM blast n=12**	**p value***
**Received CAR-T, n(%)**	30	18 (95)	12 (100)	0.42
**Evaluable at day 28**	27 (90)	15 (83)	12 (100)	0.14
**CRS, all, n(%)**	17 (57)	12 (67)	5 (32)	0.18
**CRS, grade 3-4, n(%)**	2 (7)	2 (11)	0 (0)	0.23
**ICANS, all, n(%)**	13 (43)	9 (50)	4 (33)	0.37
**ICANS, grade 3-5, n(%)**	4 (13)	4 (22)	0 (0)	0.07
**CR, day 28, n(%) (as treated)**	24 (89)	13 (72)	11 (92)	0.68
**CR, day 28, n(%) (ITT)**	24 (77)	13 (68)	11 (92)	0.72
**MRD- CR, day 28, n(%)**	24 (89)	13 (72)	11 (92)	1.0
**Relapse, n(%)**	16 (66)	8 (42)	8 (66)	0.19
**Relapse, CD19-, n(%)**	8 (13)	3 (15)	5 (25)	1.0
**Relapse, CD19+, n(%)**	8 (29)	3 (15)	5(25)	1.0

* Fisher’s exact test
CRS/ICANS rate was 54% (7/13) vs zero (0/17) for patients with >20% vs <20% marrow blasts (p=0.002; Fisher’s exact test).
Abbreviations: MRD, measurable residual disease; CRS, cytokine release syndrome; CR, complete remission; ICANS, Immune effector cell-associated neurotoxicity syndrome.

Supplementary Table 5. Adverse events in the pediatric ALL cohort

**Event**	**Any, n (%)**	**Severe (grade 3-5) n (%)**
**CRS**	17 (57)	2 (7)
**ICANS**	13 (43)	4 (13)
Leukopenia	30 (100)	28 (93)
Neutropenia	30 (100)	29 (97)
Anemia	25 (83)	21 (70)
Thrombocytopenia	20 (67)	16 (53)
Infection	6 (20)	3 (10)
Acute kidney injury	2 (7)	2 (7)
ALT/AST elevation	2 (7)	0 (0)
Capillary leak syndrome	3 (10)	0 (0)
Headache	12 (40)	1 (3)
Tremor or hyperreflexia	6 (20)	0 (0)
Vomiting	4 (13)	0 (0)
Ataxia	5 (17)	0 (0)
Aphasia	4 (13)	0 (0)
Brain edema	2 (7)	2 (7)
Seizure (clinical or abnormal EEG)	4 (13)	3 (10)
Cutaneous GVHD	2 (6)	0 (0)

Abbreviations: CRS, cytokine release syndrome; ICANS, Immune effector cell-associated neurotoxicity syndrome; GVHD, graft-versus-host disease; EEG, electroencephalogram.

Supplementary Table 6: Overall summary of toxicity and response of patients with lymphoma.

	**Patients enrolled, n=23**
**Received CAR-T, n (%)**	23
**Evaluable at day 28**	22 (96)
**CRS, all, n (%)**	12 (52)
**CRS, grade 3-4, n (%)**	1 (4)
**ICANS, all, n (%)**	5 (22)
**ICANS, grade 3-5, n (%)**	2 (9)
**CR on PET/CT as best response, n (%) (ITT)**	16 (70)
**Relapse, n (%)**	3 (13)
**Relapse, CD19-, n (%)**	1 (4)
**Relapse, CD19+, n (%)**	2 (9)

Abbreviations: CRS, cytokine release syndrome;
CR, complete remission; IIT, intention to treat;
ICANS, Immune effector cell-associated neurotoxicity syndrome.

Supplementary Table 7. Antibodies used

Fluorochrome	Antibody/Marker	Clone	Titer	Catalog Number	Company
DAPI	Viability dye	---	1:200	L23105	Invitrogen™
BV570	CD3	UCHT1	1:50	300435	Biolegend™
BUV563	CD4	SK3	1:100	612912	BD Horizon™
BUV496	CD8	RPA-T8	1:100	612943	BD Horizon™
BUV395	CD45RO	UCHL1	1:50	564292	BD Horizon™
BV421	CD27	M-T271	1:100	562513	BD Horizon™
BUV805	CCR7	3D12	1:50	741981	BD Optibuild™
BV650	41BB	4B4-1	1:50	564092	BD Horizon™
PE	TCF7	S33-966	1:20	564217	BD Pharmigen™
PE-Cy5	Ki67	SolA15	1:200	15-5698-82	eBioscience™
FITC	CD19CAR	20-291	1:25	CD9-HF251	Acro Biosystems

Supplementary Note 1: NHL study protocol

STUDY NUMBER: CASE 2417

ClinicalTrials.gov NCT #: NCT03434769

Version Date: 07/08/2020

STUDY TITLE: Phase I Clinical Trial of Anti-CD19 Chimeric Antigen Receptor T Cells for Treatment of Relapsed or Refractory Non Hodgkin Lymphoma

PRINCIPAL INVESTIGATOR: Paolo F. Caimi, MD
Case Comprehensive Cancer Center
University Hospitals Cleveland Medical Center
Seidman Cancer Center
11100 Euclid Avenue
Cleveland, OH 44106
(216) 844 - 0139
paolo.caimi@uhhospitals.org

SITE INVESTIGATOR: Armin Ghobadi, MD
Washington University School of Medicine in St. Louis
Division of Medical Oncology
Section of Stem Cell Transplant and Leukemia
660 S. Euclid Avenue
Campus Box 8007-29
St. Louis, MO 63110
(314) 747-2743
arminghobadi@wustl.edu

CO- INVESTIGATORS: **University Hospitals Seidman Cancer Center**
Kirsten Boughan, DO
Brenda Cooper, MD
Jignesh Dalal, MD
Mari Dallas, MD
Marcos de Lima, MD
Molly Gallogly, MD, PhD
Robert Maitta, MD (Pathology)
Ehsan Malek, MD

Leland Metheny, MD
Folashade Otegbeye, MD
Jane Reese-Koç
Rafick-Pierre Sekaly, PhD
Benjamin Tomlinson, MD
David Wald, MD, PhD

Washington University School of Medicine in St. Louis
Camille Abboud, MD
Nancy Bartlett, MD
Amanda Cashen, MD
Matthew Christopher, MD, PhD
John DiPersio, MD, PhD
Todd Fehniger, MD, PhD
Francesca Ferraro, MD
Meagan Jacoby, MD, PhD
Brad Kahl, MD
Miriam Kim, MD
Neha Mehta-Shah, MD
Karolyn Oetjen, MD
Iskra Pusic, MD
Mark Schroeder, MD
Nathan Singh, MD
Keith Stockerl-Goldstein, MD
Geoffrey Uy, MD
Ravi Vij, MD
Matthew Walter, MD
Lukas Wartman, MD
John Welch, MD, PhD
Peter Westervelt, MD, PhD
Zhifu Xiang, MD

STATISTICIAN: Pingfu Fu, PhD
Case Comprehensive Cancer Center
Department of Epidemiology & Biostatistics
Case Western Reserve University.
216-368-3911
pxf16@case.edu

STUDY COORDINATORS: Erin Galloway RN
Antoinette Hillian, PhD, Clinical Research Specialist
Case Comprehensive Cancer Center
University Hospitals Seidman Cancer Center

SPONSOR:	Marcos de Lima, MD
SUPPORT/FUNDING:	University Hospitals Seidman Cancer Center
SUPPLIED AGENT***(S):***	Anti-CD19 Chimeric Antigen Receptor T cells
IND #:	17932
OTHER AGENT***(S):***	Fludarabine, Cyclophosphamide

SUMMARY OF CHANGES

Protocol Date	Section	Change
03/14/18		Initial IRB approval
04/09/18	Cover page	Revised to update contact information, version date
	3.1.2	Revised infusion times to be consistent
	4.2.6	Revised exclusion criteria to clarify HIV status
	6.2.2	Revised to describe the manufacturing of fresh versus frozen products
	6.4.1	Revised to describe the administration of fresh versus frozen products
	6.4.3	Revised to clarify methods used for the thawing of frozen cells
	6.4.5	Revised to describe the administration of fresh versus frozen products
	Table 6.5.1	The Common Terminology Criteria for Adverse Event (CTCAE) version number has been updated to 5.0
	Table 6.6	The Common Terminology Criteria for Adverse Event (CTCAE) version number has been updated to 5.0
	6.8	The Common Terminology Criteria for Adverse Event (CTCAE) version number has been updated to 5.0
	8.2.3	The Common Terminology Criteria for Adverse Event (CTCAE) version number has been updated to 5.0
	9.1.1	Revised to describe the preparation, storage requirements, packaging and labeling of fresh versus frozen products
	10.1.1	Revised to clarify the blood volume needed for correlative studies
	10.1.2	Revised to clarify the blood volume needed for correlative studies
	10.1.3	Revised to clarify the blood volume needed for correlative studies
	11.1.2	Numerous corrections and administrative changes for the purpose of clarification have been made to the study parameters and the study calendar
	11.2	Numerous corrections and administrative changes for the purpose of clarification have been made to the study parameters and the study calendar
	13.1	Revised to indicate that the data would be reported in the Overture database
08/09/18	Cover page	Revised version date
	TOC	Page numbers updated due to changes made throughout the document
	4.2.5	Exclusion criteria revised to clarify what constitutes an active malignancy
	6.8	Revised to clarify the definition of dose limiting toxicity
	10.1.2	Addition of CAR-T persistence correlative assay at day +3
	11.1.1	Text added to clarify the timing of studies and evaluations during screening; addition of CAR-T persistence assay at day +3 and 7 day windows at the day

		+60 and day +90 time points
	11.2	Study Calendar was revised to be consistent with changes made to the study procedures i.e the addition of CAR-T persistence correlative assay at day +3 and the 7 day windows at the + 60 day and +90 day time points
10/31/18	Cover page	Addition of Washington University School of Medicine as a participating site
	3.4.1	Revised to clarify that subjects who discontinue study participation must still be followed for gene therapy – related adverse events per federal guidelines and patient safety
	6.4.1	The FDA reporting requirements for Serous Adverse Events have been added to this section.
	9.1	Study procedures description modified to include additional correlative samples in the event of immune-mediated adverse events. Samples will be drawn for correlative assays monitoring CAR-T cell persistence, cytokines, and T-cell subpopulation at the onset and follow-up of the immune adverse event.
5/21/19	Cover page	Revised version date
	3	Addition of expansion plan to allow enrollment of up to 12 additional subjects per dose level to further delineate toxicity protocol
	14	Statistical considerations revised to include dose expansion accrual in the possible number of subjects enrolled
06/27/19	Cover page	Revised version date
	4.3.1.2	Infusion criteria revised to allow variance in lab values deemed not clinically significant by the investigator
	4.9	Corticosteroid therapy for symptom or disease control allowed up to 7 days before infusion (changed from up to 7 days before chemotherapy)
	9.0 & 9.2	Revised schedule to allow for CAR T infusion on day 8 of culture harvest of CAR T cells
	9.0 & 9.2	Revised schedule for correlative sampling
	10.1.4	Section added to describe correlative evaluation of minimal residual disease
11/18/19	Cover page	Revised version date
	Study Schema	A reduced cyclophosphamide dosing cohort has been added to the description of the lymphodepletive regimen
	2.3	Correlative objectives were revised to add the effect that the change to the conditioning regimen might have on cytokine plasma concentrations and changes in anti-CD19 CAR-T cell subpopulations over time
	3.0	A reduced cyclophosphamide dosing cohort has been added to the description of the lymphodepletive regimen
	3.4.1	Reduced cyclophosphamide dosing has been added to the description of the lymphodepletive regimen

	6.3	Reduced cyclophosphamide dosing has been added to the description of the lymphodepletive chemotherapy
	8.1.1	The potential risks associated with Chimeric Antigen Receptor T (CAR-T) cells have been updated
	11.1.1	Text added to clarify the timing of cyclophosphamide dosing in the standard regimen versus the reduced cohort
	11.2	Text added to clarify that LVEF can be measured with either an Echocardiogram or MUGA; Text has been added to footnote 2 regarding the administration of cyclophosphamide in the standard dosing regimen versus the reduced dosing cohort
02.26.20	Cover page	Updated co-investigators for both study sites; revised version date
	3.4.3	Text has been added that reflects FDA recommendations for RCL testing
	6.11	Text has been added that reflects FDA recommendations for RCL testing
	10.1.1	Removed T+4 sample time point; contact information added for the laboratory processing blood samples for cytokine serum concentration
	10.1.2	Removed T+4 sample time point; text added to clarify that each site will be responsible for testing the cells processed and manufactured at the site.
	10.1.3	Removed T+3 sample time point; text added to clarify that each site will be responsible for testing the cells processed and manufactured at the site.
	10.1.4	Contact information added for the laboratory that will be shipping the blood samples for testing minimal residual disease.
	11.1	Day +1-day 7 revised to remove sample time points for T+3 and T+4; Text has been added that reflects FDA recommendations for RCL testing
	11.2	The Study Calendar has been revised to add RCL testing for time points 3, 6 and 12 months; Footnote 13 revised to remove sample T+4 sample time point; Footnote 16 added to reflect FDA recommendations for RCL testing;
	Appendix 4	Text has been added that reflects FDA recommendations for RCL testing
	Appendix 5	Text has been added that reflects FDA recommendations for RCL testing
07/08/2020	TOC	Page numbers updated to reflect revisions
	Throughout	Correction of typos and minor inconsistencies throughout
	Protocol summary	Interventions updated to include reduced cyclophosphamide dosing as described in protocol
	3.4.1	Duration of Therapy - updated to include Long-term safety follow-up section
	6.1	Collection of peripheral blood cells – revised to indicate collection per institutional standards to accommodate addition of participating sites
	6.4.3	Temperture for water bath for thawing product updated to 37°C ± 2°C
	6.10	The following was added to describe long-term monitoring after removal from study:<br><br>"NOTE: For subjects who discontinue their participation in the trial after CAR-T cell infusion, long-term follow up for monitoring of potential gene therapy-related delayed adverse events will continue as required per federal guidelines and as delineated in section 3.4.2. In patients who discontinue their participation after CAR-T cell infusion for reasons other than death or decision to withdraw from the study, correlative samples will continue to be collected as scheduled."
	8.2.3	Updated to clarify that adverse events will be recorded through 12 months

		post infusion.
	9.1	Packaging section updated to correct volumes of cellularproduct to be packaged in syringe or bag to align with IND
	10.1.1	Correlative section updated to clarify location of correlative sample processing
	10.1.2	Correlative section updated to clarify that flow cytometry-based phenotyping and persistence of anti-CD19 CAR-T assays will be performed by each manufacturing site.
	10.1.3	Correlative section updated to clarify that anti-CD19 CAR-T persistence assays will be performed by each manufacturing site.
	10.1.4	Processing information for samples collected for monitoring minimal residual disease updated to clarify that samples with be shipped to Case Western Reserve University and batched for shipment and processing to lab in Italy.
	11.0	Study Parameters and Calendar updated to add a separate calendar for correlative sample collection (Sec. 11.3) and to clarify study parameters section (Sec. 11.1) to align with study calendar (Sec. 11.2)
	13.1	Data Reporting section revised to reflect change in name of electronic database for data capture from Overture to ForteEDC.

STUDY SCHEMA

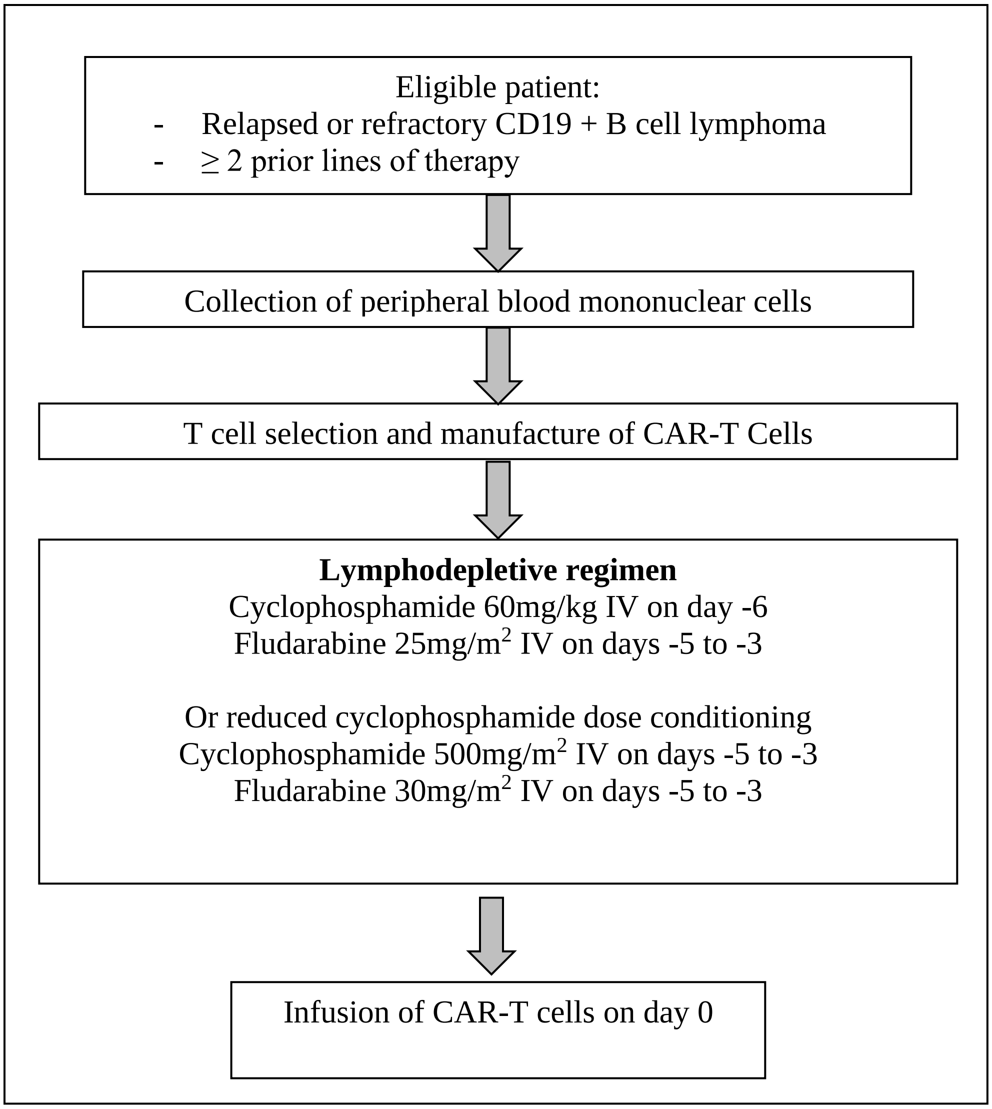

Figure 1: Study Schema. CAR-T Cells: Chimeric antigen receptor T cells

PROTOCOL SUMMARY

Protocol Number/Title	CASE 2417 Phase I Clinical Trial of Anti-CD19 Chimeric Antigen Receptor T Cells for Treatment of Relapsed or Refractory Non-Hodgkin Lymphoma
Study Phase	Phase I
Brief Background/Rationale	This study seeks to determine the safety of the infusion of autologous T cells that have been modified through the introduction of a chimeric antigen receptor targeting the B cell surface antigen CD19
Primary Objective	To determine the safety of the treatment of relapsed or refractory B cell lymphomas with chimeric antigen receptor T cells targeting CD19 and to find the recommended phase II dose for this cellular therapy
Secondary Objective(s)	• To describe the safety profile of the infusion of CAR-T cells targeting CD19.<br>• To describe the toxicities related to infusion of CAR-T cells targeting CD19.<br>• To describe the overall response rate and complete response rate of relapsed B cell malignancies treated with CAR-T cells targeting CD19.
Correlative Objective(s)	• To describe the persistence of anti-CD19 CAR-T cells, measured by flow cytometry and qPCR;<br>• To describe the T cell subpopulations of the anti-CD19 CAR-T cell product before infusion;<br>• To describe the changes in anti-CD19 CAR-T cells after infusion and their correlation with disease response and adverse events;<br>• To investigate the correlation between changes in cytokine plasma concentrations and changes in anti-CD19 CAR-T cell subpopulations over time.
Sample Size	18 subjects ≥18 years of age in dose escalation.<br>Up to 12 additional subjects per dose level may be added to further delineate safety profile
Disease sites/Conditions	C83.3 Non-Hodgkin Lymphoma
Interventions	Autologous chimeric antigen receptor T cells targeting CD19, single infusion
	Cyclophosphamide 60mg/Kg daily -6<br>(Or reduced cyclophosphamide dose conditioning: Cyclophosphamide 500mg/m^2 IV on days -5 to -3)
	Fludarabine 25mg/m^2 daily on days -5 to -3

TABLE OF CONTENTS

1 INTRODUCTION 13

1.1 NON HODGKIN LYMPHOMA 13
1.2 CHIMERIC ANTIGEN RECEPTOR – MODIFIED (CAR)-T CELLS. 13
1.3 FLUDARABINE AND CYCLOPHOSPHAMIDE LYMPHODEPLETION 17
1.4 RATIONALE 18
1.5 BACKGROUND AND RATIONALE FOR CORRELATIVE STUDIES 18

2 OBJECTIVES 18

2.1 PRIMARY OBJECTIVE 18
2.2 SECONDARY OBJECTIVES 18
2.3 CORRELATIVE OBJECTIVES 19

3 STUDY DESIGN 19

3.1 STUDY DESIGN INCLUDING DOSE ESCALATION / COHORTS 19
3.2 NUMBER OF SUBJECTS 21
3.3 REPLACEMENT OF SUBJECTS 21
3.4 DURATION OF THERAPY AND FOLLOW UP 21

4 SUBJECT SELECTION 24

4.1 INCLUSION CRITERIA 24
4.2 EXCLUSION CRITERIA 25
4.3 ELIGIBILITY FOR INFUSION OF INVESTIGATIONAL PRODUCT 26
4.4 INCLUSION OF WOMEN AND MINORITIES 27

5 REGISTRATION 27

6 TREATMENT PLAN 27

6.1 COLLECTION OF PERIPHERAL BLOOD MONONUCLEAR BLOOD CELLS 27
6.2 PROCESSING OF PERIPHERAL BLOOD MONONUCLEAR CELLS AND MANUFACTURING PROCESS TO GENERATE ANTI-CD19 CHIMERIC ANTIGEN RECEPTOR T (CAR-T) CELLS. 27
6.3 LYMPHODEPLETIVE CHEMOTHERAPY 28
6.4 STUDY DRUG 28
6.5 CYTOKINE RELEASE SYNDROME. 30
6.6 NEUROTOXICITY (CAR-T CELL – RELATED ENCEPHALOPATHY SYNDROME) 32

6.7 PHASE I DOSE ESCALATION 35
6.8 DEFINITION OF DOSE-LIMITING TOXICITY 35
6.9 GENERAL CONCOMITANT MEDICATIONS AND SUPPORTIVE CARE GUIDELINES 36
6.10 CRITERIA FOR REMOVAL FROM STUDY 36
6.11 DURATION OF FOLLOW UP 36
6.12 RETREATMENT 38
6.13 PROHIBITED MEDICATIONS 39

7 DOSE DELAYS/DOSE MODIFICATIONS 39

7.1 CYCLOPHOSPHAMIDE 39
7.2 FLUDARABINE 39
7.3 ANTI-CD19 CAR-T CELLS 39

8 ADVERSE EVENTS AND POTENTIAL RISKS 40

8.1 ADVERSE EVENTS 40
8.2 DEFINITIONS 48
8.3 SAE REPORT FORM 51
8.4 REPORTING PROCEDURES FOR SERIOUS ADVERSE EVENTS 51
8.5 SAES AND ONCORE 53
8.6 DATA SAFETY AND TOXICITY COMMITTEE 53
8.7 DATA AND SAFETY MONITORING PLAN (DSMP) 53

9 PHARMACEUTICAL INFORMATION 53

9.1 INVESTIGATIONAL AGENTS 53
9.2 COMMERCIAL AGENT 54

10 EXPLORATORY OR CORRELATIVE STUDIES 57

10.1 METHODS 58

11 STUDY PARAMETERS AND CALENDAR 60

11.1 STUDY PARAMETERS 60
11.2 CALENDAR 66

12 MEASUREMENT OF EFFECT 68

12.1 LYMPHOMA RESPONSE CRITERIA 68
12.2 DEFINITIONS OF TIME PERIODS 73
12.3 RESPONSE REVIEW 74

13 DATA REPORTING / REGULATORY CONSIDERATIONS 74

13.1 DATA REPORTING 74
13.2 REGULATORY CONSIDERATIONS 74

14 STATISTICAL CONSIDERATIONS 76

14.1 CORRELATIVE STUDIES 77

15 REFERENCES 78

16 APPENDICES 80

16.1 APPENDIX 1. PERFORMANCE STATUS CRITERIA 80
16.2 APPENDIX 2. CELL PRODUCT RELEASE FORM 81
16.3 APPENDIX 3. MANAGEMENT OF ADVERSE EVENTS FOLLOWING IMMUNE EFFECTOR CELL THERAPY (UHSCC SOP) 82
16.4 APPENDIX 4: LONG TERM FOLLOW UP CHECKLIST. YEARS 1 – 5 AFTER TREATMENT. 90
16.5 APPENDIX 5. LONG TERM FOLLOW UP CHECKLIST. YEARS 6 - 15 AFTER TREATMENT. 91
16.6 APPENDIX 6. CARTOX-10 NEUROLOGIC ASSESSMENT OF CAR-T – ASSOCIATED ENCEPHALOPATHY (19) 92

1.0 Introduction

1.1 Non Hodgkin Lymphoma

Non-Hodgkin lymphoma (NHL) represents the seventh most common malignancy in adults, with an estimated 72,240 new cases expected to be diagnosed in the United States in 2017[1]. While the treatment of mature B cell lymphomas has demonstrated significant improvements in the last 30 years, more than 30% of patients will die within 5 years of diagnosis, and a much larger proportion of patients will present with relapsed disease[1].

The treatment of relapsed and refractory NHL, regardless of the histologic subtype, remains challenging. Once the disease has become resistant to conventional chemoimmunotherapeutic and targeted approaches, there are limited options that can provide long – term disease control.

The addition of rituximab, the first monoclonal antibody targeting CD20, to chemotherapy remains the most important innovation in the therapeutic armamentarium of mature B cell malignancies in the last two decades. This agent has resulted in improvements in the treatment of diffuse large B cell lymphoma (DLBCL) [2], follicular lymphoma and other indolent lymphomas[3-5].

Several new targeted agents have demonstrated activity in subsets of mature B cell lymphomas (idelalisib, ibrutinib, venetoclax), but few have a wide applicability as immunotherapy.

1.2 Chimeric antigen receptor – modified (CAR)-T cells.

1.2.1 Preclinical Data

T cells are a component of the adaptive immune system, as effectors of cell mediated-immunity. T cells exert their cytotoxic and potentially anti-tumoral effect upon engagement of the T – cell receptor is by a cognate peptide antigen, which is presented in the context of a specific major histocompatibility complex (MHC) molecule. The concept of tumor-targeted T cells has come to a reality as a result of genetic modification strategies capable of generating a tumor – targeting T cell receptor. Chimeric antigen receptors (CAR) are recombinant T cell receptors composed of an extracellular fragment derived from immunoglobulin variable fragment, as single chain (scFv), which is in turn linked to intracellular signaling sequences that are derived from T cells. Insertion of a CAR in a T cell can induce activation of the T cell upon ligation of the scFv with its target antigen.

1.2.1.1 Institutional experience/ process validation /performance qualification

Between October 2017 and Nov 2017, two validation/PQ CAR-T manufacture runs were performed on the Prodigy TCT program, using normal donor leukopaks as starting material. Both procedures occurred without complication. Products passed the release criteria defined in Table 1.2.1.1 below. Culture and product characterization results are listed in Tables 1.2.1.1 and 1.2.1.2 respectively.

Table 1.2.1.1 Cell culture parameters			
	WBC dose loaded on Prodigy	Culture starting dose/ CD4 and CD8 purity	End of culture cell yield
Run 1	$9.2x10^8$	$1x10^8$<br>72.6 %	$4.3x10^9$
Run 2	$7.4x10^8$	$9.5x10^7$<br>78%	$3.9x10^9$

Table 1.2.1.1 demonstrates that both products expanded to clinically applicable doses. Run 1 resulted in a 43-fold T cell expansion. Run 2 resulted in a 41-fold T cell expansion.

**Table 1.2.1.2. T cell subpopulation characteristics.**								
	Viable CD45	Viable CD3	Viable CD4	Viable CD8	Viable CD4/8	Viable CD20	Viable CD4 CAR+	Viable CD8 CAR+
**Run 1 fresh**	99.9%	98.1%	79.98%	20.35%	0.51%	0	19.5	5.6
**Run 1 thawed**	99.8%	98.4%	70.05%	16.6%	7.84%	0	15.4	15.3
**Run 2 fresh**	99.6%	98.8%	67.3%	31.1%	0.46%	0	17.5	6.9
**Run 2 thawed**	99.8%	98.9%	60.15%	37.0%	1.70%	0	2.5	6.2

Table 1.2.1.2 demonstrates a highly pure T-cell population in the final product. Monocyte and NK cells populations (not shown) were <1%. Phenotypes were maintained upon thaw. Cells assessed for thaw phenotype were cryopreserved and stored in the vapor phase of liquid nitrogen for 1 month. Total product viability was 90% and 82% for validation runs 1 and 2, respectively, when assessed by 7-AAD. CD19+ cells were detected using a Recombinant Human CD19 Fc Chimera Protein primary antibody (R&D Systems) and a PE conjugated- AffiniPure F(ab')2 Fragment Goat AntiHuman IgG, Fcγ Fragment Specific antibody (Jackson Immunoresearch). All surface markers were detected using fluorescein-labeled antibodies (Miltenyi Biotech).

1.2.2 Clinical Data

B-ALL

Park and colleagues published the results of 51 subjects with B-cell acute lymphoblastic leukemia (B-ALL) treated with CAR-T cells after lymphodepletion regimens containing cyclophosphamide alone or combined with fludarabine [6]. CAR-T doses ranged from 1 x 10^6 to 3 x 10^6 cells/kg. Among 50 evaluable patients, 41 (82%) had a morphologic complete remission, of whom 33 had no minimal residual disease (MRD) by flow cytometry. Cytokine release syndrome (CRS) was more common among patients with morphologic disease (i.e. containing >5% blasts in bone marrow), with 13/31 subjects having CRS in

this group compared with 1 of 20 subjects with no morphologic disease. Among patients with higher tumor burden, three patients died after receiving $3x10^6$ CAR-T cells/kg, suggesting patients with high tumor burden should receive smaller numbers of cells. Neurotoxicity occurred in 15 patients.

Investigators from the National Cancer Institute have reported the outcomes of 38 B-ALL patients treated with 19-28z CAR-T cells [7]. The intensity of the conditioning regimen was adjusted according to the amount of tumor burden, with patients having 25% or more blasts in bone marrow treated with high intensity conditioning regimens whereas those with lower tumor burden were treated with cyclophosphamide and fludarabine. The maximum tolerated dose of 1×10^6 CAR-T cells/kg was determined in the initial cohort of 20 patients and used in the subsequent 18 subjects. Twenty patients (58%) achieved MRD-negative complete remission, with leukemia free survival at 18 months of 45.5%. Grade 4 CRS was observed in 16% of patients in phase 1 and 5.6% in the expansion cohort. No neurotoxicity was reported in this cohort.

Researchers from the University of Pennsylvania have reported the results in studies done in children and young adults [8]. Fifty-nine subjects younger than 24 years of age were treated with $1 – 10 \times 10^7$ total T cells/kg with CAR transduction efficacy ranging from 2.3 – 45% with multiple lymphodepletion regimens. CAR-T cells were composed of anti-CD19 scFv, CD3z, and 4-1BB domains, activated/expanded ex vivo with anti-CD3/CD28 beads. MRD negative disease by flow cytometry occurred in 55 (93%) of patients. Relapse occurred in 20 patients, 13 with CD19 negative disease. Relapse free survival and overall survival at 12 months were 55% and 79%. Cytokine release syndrome of any grade occurred in 88% of cases, and was severe in 27%. The results of 27 adults with B-ALL treated at University of Pennsylvania with fixed – doses of CAR-T (5×10^7 or 5×10^8 cells) in single or split infusions were reported by Frey and colleagues [9]. Among 9 patients treated with single 5×10^7 infusion, three achieved CR, while 3 out of 6 achieved CR when treated with single infusion of 5×10^8 CAR-T cells and 3 died of cytokine release syndrome. Fractionation of the 5×10^7 cell dose resulted in 75% complete remission rate; the incidence of grade 3-4 CRS was 75% with no fatal events.

Investigators from the Fred Hutchinson Cancer Research Center conducted a trial with CAR-T cells formulated with a defined ratio of CD4+ to CD8+ cells of 1:1, based on prior data indicating that CAR-T cells manufactured from central memory T cells or naive T cells were more effective at tumor elimination than anti CD19 CAR-Ts manufactured from effector T cells [10]. Lymphodepletion was done with one of four regimens (cyclophosphamide, cyclophosphamide + etoposide, fludarabine x 3 days + cyclophosphamide or fludarabine x 5 days + cyclophosphamide). CAR-T cell product was given at doses of $2x10^5$, $2x10^6$ and $2x10^7$ cells/kg. Of 30 enrolled patients, 27 of 29 evaluable patients (94%) achieved a flow cytometry-negative CR; two of these patients were found to have MRD by molecular testing. There were two severe toxicities at the highest dose level and treatment was not continued at this dose. Tumor burden was a risk factor for development of toxicity, and a risk-adapted approach was used, where patients with >20% marrow involvement received 2×10^5 cells/kg while those with ≤20% received

2×10^6 cells/kg. After adoption of this approach, only one of ten patients developed severe CRS requiring ICU care. With cyclophosphamide alone, early relapse was common and associated with loss of CAR-T cells due to anti-CAR-T cell immune response directed at epitopes of murine scFv. Addition of Fludarabine decreased host response and improved persistence of CAR-T cells and clinical outcomes.

Non-Hodgkin lymphoma

Investigators from the NCI reported the results of CAR-T cell treatment of 11 B-NHL patients and four CLL patients [11]. Nine of the 11 NHL patients had aggressive disease on histology (four with diffuse large B-cell lymphoma (DLBCL), four with primary mediastinal B-cell NHL, one with Richter's transformation to DLBCL after CLL) and two had indolent disease. Patients received lymphodepletion with high-dose Cy (60–120 mg/kg) followed by 5 days of Flu 25 mg/m2, with infusion one day later of $1–5 \times 10^6$ CAR-T cells/kg. Of the nine patients with aggressive histology, seven were evaluable, with four patients achieving a CR and two achieving a partial response (PR). Three of the four CLL patients achieved a CR. Adverse events included grade ≥3 hypotension in four of 15 (27%) patients and neurotoxicity in six of 15 (40%) patients. One patient died on day 16 from an unclear etiology.

Kochenderfer and colleagues reported outcomes of 22 patients given low-dose cyclophosphamide (300–500 mg/m^2) for 3 days, with concurrent Fludarabine 30 mg/m2 administration as lymphodepletion [12]. Eight of 19 DLBCL patients achieved a CR with an overall response rate (ORR) in DLBCL of 68% (13 of 19 patients). One MCL patient and two FL patients obtained CR.

Investigators from the University of Pennsylvania reported on the results of 24 NHL patients treated with 3.08×10^6 to 8.87×10^6 CAR-T cells/kg following a range of lymphodepletion regimens [13]. Eight of 11 patients with follicular lymphoma, seven of 15 patients with DLBCL, and one of two patients with mantle cell NHL responded, with an ORR of 68%. Sixteen of 24 patients developed CRS and three patients developed neurotoxicity.

Investigators from the Fred Hutchinson Cancer Research Center reported the results of 32 patients with non-Hodgkin B cell lymphoma, (11 DLBCL, 10 transformed indolent lymphomas, 5 follicular lymphoma and 4 mantle cell lymphoma) [14]. Of the 30 evaluable patients, ten (33%) had a CR and nine (30%) a PR, giving an ORR of 63%. Severe CRS requiring ICU care was seen in four of 32 (12.5%) patients, and grade ≥3 neurotoxicity was noted in nine of 32 (28%) patients. As observed in previous studies in B-ALL patients, addition of fludarabine to cyclophosphamide-based lymphodepletion improved CAR-T cell expansion, persistence, and clinical outcomes in NHL patients. Patients treated at the highest CAR-T cell dose (2×10^7 cells/kg) after cyclophosphamide and fludarabine lymphodepletion experienced more toxicity; therefore, 2×10^6 cells/kg was deemed the maximum tolerated dose. Infusion of this dose after cyclophosphamide and fludarabine lymphodepletion to 11 patients resulted in a CR rate of 64% and an ORR of 82%. Of note, the investigators found a relationship between fludarabine/cyclophosphamide conditioning

and persistence of CAR-T cells over time that was not observed in subjects conditioned with cyclophosphamide alone or with cyclophosphamide combined with etoposide. Of particular relevance, patients treated with combination fludarabine and cyclophosphamide had increased depth of response and in 6/20 patients the reduction of tumor burden 1 month after infusion was not the maximal response, suggesting continued therapeutic efficacy associated with increased persistence of CAR-T cells. In addition, three of four patients who received Cy/Flu before their first CAR-T cell infusion and had a second infusion to treat persistent disease had evidence of further tumor regression after the second infusion. Twenty of 32 patients developed CRS, with only 4 subjects presenting severe CRS requiring intensive care management. Neurotoxicity was more common in patients receiving fludarabine and cyclophosphamide lymphodepletion, and was present in 9 of 32 subjects. The neurologic events included reversible encephalopathy alone (n = 5) or with tremor (n = 1) or speech disturbance (n = 1). Choreoathetosis and fatal intracranial hemorrhage were observed in one patient each. T cell dose was related to the development of sCRS and neurotoxicity, with three of six patients (50%) treated at 2×10^7 CAR-T cells/kg after Cy/Flu lymphodepletion developing sCRS and four of six (67%) patients developing grade ≥3 neurotoxicity.

Neelapu and colleagues[15] recently reported the multicenter experience with anti CD19 Axicabtagene ciloleucel (axi-cel®, Kite Pharma) (n=111). Lymphodepleting chemotherapy consisted of fludarabine (30 mg/m2) and cyclophosphamide (500 mg/m2) on days −5, −4, and −3 before the administration of axi-cel at a target dose of 2×106 CAR T cells/kilogram on day 0. One hundred and one patients received the treatment, including 77 with diffuse large B-cell lymphoma and 24 with primary mediastinal B-cell lymphoma or transformed follicular lymphoma. Response rate was 82%, with a 54% CR rate. Adverse events are reviewed in section 4.5, above.

The University of Pennsylvania experience treating 38 patients with NHL was described by Schuster and coworkers[16]; 28 patients received treatment as planned. The median total CAR-T cell dose was 5.00×108 (range, 1.79×108 to 5.00×108), while the median cell dose per kilogram was 5.79×106 (range, 3.08×106 to 8.87×106). Response rate at 3 months was 64% (95% [CI], 44 to 81).

Different costimulatory molecules were used in these studies (CD28 vs 4-1BB), but it is unclear if there is a difference in clinical activity or CAR-T cell persistence.

1.3 Fludarabine and Cyclophosphamide Lymphodepletion

The majority of studies conducted with Anti-CD19 CAR-T cells for treatment of B-ALL, NHL and chronic lymphocytic leukemia have utilized some form of lymphodepletion to facilitate CAR-T cell persistence. The results published by investigators from the Fred Hutchinson Cancer Research Center [14] highlight the importance of including fludarabine to cyclophosphamide in the lymphodepletion regimen. Patients who received the combination of fludarabine and cyclophosphamide had higher serum concentrations of interleukin-7 (IL-7) (P = 0.014) and IL-15 (P < 0.001) on the day of CAR-T cell infusion. In addition, there were higher number of CAR-T cells at the 1 month and 3 month

evaluations. Sixteen of 18 patients who received Cy/Flu lymphodepletion and survived more than 28 days after CAR-T cell infusion had detectable CAR-T cells in the blood by QPCR (>10 copies/μg of DNA) at the last follow-up (range, 34 to 349 days). The doses used by Turtle and colleagues were: Cyclophosphamide 60mg/m^2 IV on the first day of conditioning and Fludarabine 25mg/m^2 IV daily on days 2 - 4 (3 days) or 2 - 6 (5 days). There were no reported differences in outcomes with different fludarabine doses.

1.4 Rationale

There is a persistent need for development of new, effective therapies for treatment of B cell malignancies. Therapy with chimeric antigen receptor – modified (CAR)-T cells has demonstrated activity against these disorders in early phase studies conducted in a limited number of centers. Our study will confirm the activity of anti-CD19 CAR-T cells against relapsed CD19 positive lymphoma using a single lymphodepletion regimen and using a CAR-T cell manufacturing process that can be replicated in multiple academic institutions with the appropriate cellular manufacturing facilities. If successful, this study will demonstrate CAR-T cell therapy may have widespread applicability with local manufacturing processes.

1.5 Background and Rationale for Correlative Studies

Several human studies have demonstrated the anti-lymphoma activity of anti-CD19 CAR-T cells [15,16]. There are no clear laboratory measures that can predict the CAR-T cell product's potential for subsequent expansion, antitumoral activity. A recent study by Neelapu and colleagues [15] observed that the area under the CAR-T cell expansion had significant association with response. Inflammatory and cell growth – related cytokines and other biomarkers (i.e. interleukins, granzyme B, ferritin) have shown variable correlation with the development of neurologic symptoms as well as cytokine release syndrome. A report by Kochenderfer and colleagues [17] indicates interleukin-15 levels correlate with CAR-T cell product expansion and lymphoma response to this cellular therapy.

In our clinical trial we plan on focusing our correlative assays to the following parameters

1. Anti-CD19 CAR-T product characteristics at the time of infusion;
2. Anti-CD19 CAR-T cell subpopulation changes over time;
3. Changes in serum concentration of cytokines and effects on response, adverse effects and changes in CAR-T cell subpopulations over time;
4. The effects of prior parameters on CAR-T cell persistence, measured by flow cytometry and qPCR.

2 Objectives

2.1 Primary Objective

- To determine the safety of the treatment of relapsed or refractory B cell lymphomas with chimeric antigen receptor T cells targeting CD19 and to find the recommended phase II dose for this cellular therapy

2.2 Secondary Objectives

- To describe the safety profile of the infusion of CAR-T cells targeting CD19.

- To describe the toxicities related to infusion of CAR-T cells targeting CD19.
- To describe the overall response rate and complete response rate of relapsed B cell malignancies treated with CAR-T cells targeting CD19.

2.3 Correlative Objectives

- To describe the persistence of anti-CD19 CAR-T cells, measured by flow cytometry and qPCR;
- To describe the T cell subpopulations of the anti-CD19 CAR-T cell product before infusion;
- To describe the changes in anti-CD19 CAR-T cells after infusion and their correlation with disease response and adverse events;
- To investigate the correlation between changes in cytokine plasma concentrations and changes in anti-CD19 CAR-T cell subpopulations over time and with changes in conditioning regimen.

3 Study Design

3.1 Study design including dose escalation / expansion

This is a phase I study with "3 + 3" design. Patients will be enrolled sequentially to each dose level defined in Table 3.1, starting with dose level 1. The CAR-T cell dose escalation schema is presented in table 3.1.

Table 3.1. Dosing Schema			
Dose Level	Anti CD19 CAR-T Cell Dose *	Fludarabine (mg/m^2 from day -5 to -3)	Cyclophosphamide (mg/Kg/IV on day -6)
Level -1	1 x 10^5 cells/kg	25	60
Level 1 [Starting Dose]	5 x 10^5 cells/kg	25	60
Level 2	1 x 10^6 cells/kg	25	60
Level 3	2 x 10^6 cells/kg	25	60
*CAR-T cell dose will be at or as close as possible to the pre-specified dose level, with a ±20% variation.			

If none of 3 patients experiences a dose limiting toxicity (DLT), dose escalation may proceed and subjects will be entered at one dose level higher. If 1/3 experiences a DLT, 3 more patients will be entered at the same dose level. If no further DLT is observed (1/6), dose escalation may proceed and new subjects will be entered at one dose level higher. However, if 2 or more subjects experience DLT at dose level 1, enrollment will revert to dose level -1. If 1/3 experiences a DLT, 3 more patients will be entered at dose level -1. If no further DLT is observed (1/6), dose escalation may proceed and new subjects will be entered again at dose level 1. However, if 2 or more subjects experience DLT on dose level -1, the maximum tolerated dose (MTD) has been exceeded. No further patients will be enrolled to the study. Should enrollment return to dose level 1 after completing dose level -1, a total of 6 new patients will be entered at this dose level and dose escalation beyond dose level 1 will be done after subject safety events are evaluated by Principal Investigator

and IND Sponsor.

The MTD is therefore defined as the dose level immediately below that in which ≥ 2/6 subjects experience a DLT. A total of 6 patients will be enrolled at the MTD.

Expansion plan
In dose levels that have completed enrollment per dose escalation and which have not exceeded the maximum tolerated dose, enrollment may be expanded to further delineate toxicity.

Each dose level that has not exceeded the maximum tolerated dose can be expanded by up to 12 patients. Expansion will be decided by the Principal Investigator and trial slot assignment to an expansion cohort will be done only when dose escalation slots have been completed.

Reduced cyclophosphamide cohort
Three to 6 patients will be treated with reduced cyclophosphamide dosing during lymphodepletive regimen. Enrollment of subjects with reduced cyclophosphamide dosing (500mg/m^2 x 3 days with Fludarabine 30mg/m^2 x 3 days) will be done only once a specific dose level is considered safe at full lymphodepletive dosing (i.e. 3 patients enrolled without DLT or 1/6 subjects with DLT).

3.1.1 Staggered enrollment
Subjects will be enrolled in this trial in a staggered fashion. The first three subjects in each dose cohort will receive the planned anti-CD19 CAR-T cell product infusion no sooner than 14 days from each other.

If re-escalation occurs (as delineated in section 3.1), then subjects enrolled in each cohort will receive the planned anti-CD19 CAR-T cell product infusion no sooner than 30 days from each other.

3.2 Number of Subjects

Dose Escalation

The minimum number of 4 subjects would be enrolled if (2/3) dose–limiting toxicities were to be observed in the initial dose level (level 1) and (2/3) dose–limiting toxicities were to be observed in dose level -1. The study would be closed because of excessive toxicity.

If no dose-limiting toxicities are observed in the 3 planned cohorts; 9 patients would be enrolled in the 3 dose–escalation cohorts (three in each cohort). If 1/6 dose-limiting toxicities were to be observed in each planned treatment cohort; a maximum number of 18 patients would be enrolled in the dose escalation part of the study (six in each cohort). The same number of subjects (18) would occur if 6 patients were enrolled in dose level 1, -1 and then back in 1 after re-escalation.

Dose Level Expansion

If dose levels are expanded to further evaluate toxicity, up to 12 additional subjects may be added per dose level for a maximum of 36 additional patients if all 3 dose levels were expanded to the maximum.

3.3 Replacement of Subjects

There is no plan for replacement of subjects.

3.4 Duration of therapy and follow up

3.4.1 Duration of Therapy

This study involves infusion of anti-CD19 CAR-T cells administered after an immunosuppressive conditioning regimen of fludarabine and cyclophosphamide. As such, the therapy is given only once but consists of several parts:

1. **Autologous lymphocyte/mononuclear cell collection**. Lymphocytes will be collected through standard apheresis procedures as per Standard Operating Procedures of University Hospitals Seidman Cancer Center.
2. **CAR-T cell manufacturing**. This procedure will occur over approximately 8-12 days.
3. **Lymphodepletion** (**6 days = 4 days of therapy and 2 days of rest; or 5 days for reduced cyclophosphamide dose group = 3 days of therapy and 2 days or rest)**. 4 days of immunosuppressive chemotherapy. Cyclophosphamide given at a dose of 60mg/kg/IV on day -6 and Fludarabine 25mg/m^2/IV on days -5 to -3, inclusive. For the reduced cyclophosphamide dose group the treatment will consist of 3 days of immunosuppressive therapy. Cyclophosphamide given at a dose of 500mg/m^2/IV on day -5 to -3 and Fludarabine 30mg/m^2/IV on days -5 to -3, inclusive.
4. **CAR-T Cell Infusion.** The infusion of CAR-T cells targeting CD19 will occur over 5-30 minutes.
5. **Early post-infusion.** From day 1 to day 30 after infusion. During this time,

subjects will be observed closely for the development of acute symptoms and complications related to the infusion of CAR-T cells. At the end of this period (T+30 ±7 days) a response assessment will be performed.

6. **The long-term post-infusion period.** From day 30 after infusion onwards. During this period of time, patients will have clinical follow up as needed and will have imaging studies to determine their disease status. Additional follow up will be done per their clinical manifestations but patients will continue to be enrolled in the trial for up to 12 months if in continued remission.
7. **Long-term safety follow up.** From year 1 up to year 15. Long term monitoring of patient safety and presence of viral vector.

Participation in this study will continue for the period of time specified unless one of the following occurs. Because the collection of T cells, the lymphodepletive regimen and infusion of CAR-T cells occurs only once, it is not possible to discontinue these interventions once they have occurred. However, the subjects' participation on the trial will be stopped (either before or after infusion) if one of the following occurs:

- Disease progression, treatment failure or recurrence at any time;
- Intercurrent illness that prevents infusion or follow up post-infusion;
- Pregnancy during the course of the study for child-bearing participants;
- The investigator considers it, for safety reasons, to be in the best interest of the patient;
- General or specific changes in the patient's condition render the patient unacceptable for treatment (before infusion) or follow-up (after infusion) in the judgment of the investigator,
- Patient decision to withdraw prior to infusion or from post-infusion follow-up,
- Death

The Sponsor reserves the right to temporarily suspend or prematurely discontinue this study. The date and reason for discontinuation must be documented. Every effort should be made to complete the appropriate assessments.

NOTE: For subjects who discontinue their participation in the trial after CAR-T cell infusion, long term follow up for monitoring of potential gene therapy – related delayed adverse events will continue as required per federal guidelines and as delineated in section

3.4.2. In patients who discontinue their participation after CAR-T cell infusion for reasons other than death or decision to withdraw from the study, correlative samples will continue to be collected as scheduled.

3.4.3 Duration of Follow Up

Patients will be followed for dose-limiting toxicity for 30 days after the day of infusion of CAR-T cells or until death, whichever occurs first. Patients will be followed for survival until 12 months after CAR-T cell therapy.

Patients will be monitored for replication competent lentivirus (RCL), based on FDA recommendations, by analysis of peripheral blood mononuclear cells by quantitative PCR for the envelope gene sequence VSV-G. Blood samples will be obtained at pretreatment and post CART infusion at 3, 6, 12 months and yearly up to 15 years. If post infusion samples are negative for the first 12 months, yearly review of medical history will suffice and follow up samples can be discontinued.

The clinical course of each adverse event will be followed until resolution, stabilization, or until it has been determined that the study treatment or participation is not the cause.

Serious adverse events that are still ongoing at the end of the study period will necessitate follow-up to determine the final outcome. Any serious adverse event that occurs after the study period and is considered to be possibly related to the study treatment or study participation will be recorded and reported immediately.

Follow up will be 15 years for monitoring for potential gene therapy – related delayed adverse events.

During the first 5 years after treatment with Anti-CD19 CAR-T cells, subject follow up will be done annually in clinical visits, in person, following the form delineated in Appendix 4, which will record the following information:

- Exposure to mutagenic agents;
- Exposure to medicinal products;
- History;
- Physical examination;
- Laboratory test results including assay for persistent vector sequence;
- Emergence of new conditions:
    - New malignancy(ies)
    - New incidence or exacerbation of a pre-existing neurologic disorder
    - New incidence or exacerbation of a pre-existing rheumatologic or autoimmune disorder
    - New incidence of a hematologic disorder.

During the subsequent 10 years (years 6 – 15 after treatment with Anti-CD19 CAR-T cells), subjects will be contacted annually. If office visits are not scheduled, then patients may be contacted by telephone or written questionnaire. Contact throughout years 6 – 15 will be done following the form in Appendix 5, which will record the following information:

- Emergence of new conditions, including but not limited to:
    - New malignancy(ies)
    - New incidence or exacerbation of a pre-existing neurologic disorder
    - New incidence or exacerbation of a pre-existing rheumatologic or autoimmune disorder
    - New incidence of a hematologic disorder.

During years 6 – 15 after treatment, subjects with previous laboratory results indicating

vector persistence will have this test repeated annually until resolution.

All subjects will be provided with the study coordinator contact information and encouraged to report delayed adverse events, including unexpected illness or hospitalization.

4 Subject Selection

Each of the criteria in the sections that follow must be met in order for a subject to be considered eligible for this study. Use the eligibility criteria to confirm a subject's eligibility.

Subject's Name____

Medical Record # ____

Research Nurse / Study Coordinator Signature: _____________________________

Date __________

Treating Physician [Print] ____

Treating Physician Signature: ___________________________ Date _____________

4.1 Inclusion Criteria

Subjects must meet all the following inclusion criteria to be eligible for enrollment:

4.1.1. Subjects must have relapsed or refractory non-Hodgkin lymphoma treated with at least two lines of therapy. Disease must have either progressed after the last regimen or presented failure to achieve partial or complete remission with the last regimen.

4.1.2. The patient's lymphoma must be CD19 positive, either by immunohistochemistry or flow cytometry analysis on the last biopsy available.

4.1.3. Age ≥18 years.

4.1.4. ECOG Performance status ≤ 2 [See Appendix I]

4.1.5. Total bilirubin ≤ 1.5 times the institutional upper limit of normal

4.1.6. AST (SGOT) ≤ 3 X institutional upper limit of normal

4.1.7. ALT (SGPT) ≤ 3 X institutional upper limit of normal

4.1.8. Serum Creatinine ≤ 2 X the institutional upper limit of normal

4.1.9. Subjects must have the following hematologic function parameters:

4.1.9.1. ANC>1,000/uL

4.1.9.2. Absolute Lymphocyte Count >100/uL

4.1.9.3. Platelets >50,000/uL

4.1.10. Subjects must have the ability to understand and the willingness to sign a written informed consent document.

4.2 Exclusion Criteria

The presence of any of the following will exclude a subject from study enrollment.

4.2.1 Autologous transplant within 6 weeks of planned CAR-T cell infusion.

4.2.2 History of allogeneic stem cell transplant.

4.2.3 Recipient of CAR-T cell therapy outside of this protocol.

4.2.4 Active central nervous system or meningeal involvement by lymphoma. Subjects with untreated brain metastases/CNS disease will be excluded from this clinical trial because of their poor prognosis and because they often develop progressive neurologic dysfunction that would confound the evaluation of neurologic and other adverse events. Patients with a history of CNS or meningeal involvement must be in a documented remission by CSF evaluation and contrast-enhanced MRI imaging for at least 90 days prior to registration.

4.2.5 Active malignancy, other than non-melanoma skin cancer, carcinoma in situ (e.g. cervix, bladder, breast)

4.2.6 HIV seropositivity.

4.2.7 Subjects with uncontrolled intercurrent illness including, but not limited to ongoing or active infection, symptomatic congestive heart failure, unstable angina pectoris, cardiac arrhythmia, pulmonary abnormalities or psychiatric illness/social situations that would limit compliance with study requirements.

4.2.8 Pregnant or breastfeeding women are excluded from this study because CAR-T cell therapy may be associated with the potential for teratogenic or abortifacient effects. Women of child bearing potential must have a negative serum pregnancy

test. Because there is an unknown, but potential risk for adverse events in nursing infants secondary to treatment of the mother with CAR-T cells, breastfeeding should be discontinued. These potential risks may also apply to other agents used in this study.

4.2.9 Evidence of myelodysplasia or cytogenetic abnormality indicative of myelodysplasia on any bone marrow biopsy prior to initiation of therapy

4.2.10 Serologic status reflecting active hepatitis B or C infection. Patients that are positive for hepatitis B core antibody, hepatitis B surface antigen (HBsAg), or hepatitis C antibody must have a negative polymerase chain reaction (PCR) prior to enrollment. (PCR positive patients will be excluded.)

4.2.11 Patients with history of clinically relevant CNS pathology such as epilepsy, seizure disorders, paresis, aphasia, uncontrolled cerebrovascular disease, severe brain injuries, dementia and Parkinson’s disease.

4.2.12 History of autoimmune disease (i.e. rheumatoid arthritis, systemic lupus erythematosus) with requirement of immunosuppressive medication within 6 months.

4.3 Eligibility for Infusion of Investigational Product

Subjects will undergo a **second** evaluation of eligibility on day -2 or -1 prior to infusion of anti-CD19 CAR-T cell product. This eligibility criterion will include the inclusion and exclusion criteria required for enrollment with the following exceptions and additions

4.3.1 Inclusion criteria exceptions:

4.3.1.1 Hematologic function parameters will not be included as a pre-infusion eligibility criterion (because lymphodepletive chemotherapy is expected to cause pancytopenia).

4.3.1.2 Laboratory result abnormalities that are considered not clinically significant by the principal investigator, AND are not the result of a demonstrated active infection or an active central nervous system condition.

4.3.2 Exclusion criteria additions:

4.3.2.1 Use of corticosteroids within 7 days prior to infusion (with exception of agents used for prevention of emesis during lymphodepletive chemotherapy)

4.3.2.2 Neurologic symptoms suggestive of an active central nervous system condition.

4.3.2.3 Signs or laboratory markers of active infection or systemic inflammatory response.

4.4 Inclusion of Women and Minorities

Men, women and members of all races and ethnic groups are eligible for this trial.

5.0 Registration

All subjects who have been consented are to be registered in the OnCore™ Database. For those subjects who are consented, but not enrolled, the reason for exclusion must be recorded.

All subjects will be registered through University Hospitals Seidman Cancer Center and will be provided a study number by contacting the study coordinator listed on the cover page.

6.0 Treatment Plan

Because this is a cellular therapy clinical trial and the investigational intervention is a single event of CAR-T cell infusion, there is no plan for dose modification of CAR-T cells, cyclophosphamide or fludarabine after initiation. Patients who initially present with a calculated creatinine clearance less than 50 ml/min will receive fludarabine at a dose adjusted according to Section 7.2. Once fludarabine has been initiated at the adjusted dose, there will be no further modifications.

Reported adverse events and potential risks of CAR-T cells, cyclophosphamide and fludarabine are described in Section 8.0.

No investigational or commercial agents or therapies other than those described below may be administered with the intent to treat the patient's malignancy.

6.1 Collection of peripheral blood mononuclear blood cells

Once the patient has met eligibility criteria and provided consent, he/she will undergo an apheresis procedure for collection of peripheral blood mononuclear cells for CAR-T manufacturing, according to institutional standards.

6.2 Processing of peripheral blood mononuclear cells and manufacturing process to generate Anti-CD19 chimeric antigen receptor T (CAR-T) cells.

6.2.1 Processing and manufacturing facility:

The site of processing of peripheral blood mononuclear collection products and subsequent Anti-CD19 CAR-T cell manufacture will be University Hospitals Seidman Cancer Center Cellular Therapy Laboratory (UHSCC-CTL) located in the Wolstein Research Building, which is compliant with federal regulations and FACT standards for more than minimally manipulated cell products

6.2.2 Anti-CD19 CAR-T cell manufacturing

Anti-CD19 CAR-T cells will be manufactured according to the processes described in IND # 17932. After cell cultures are completed and release criteria specified in the IND are met, the CAR T cells may be released for infusion to the patient or cryopreserved in infusible cryomedia.

6.3 Lymphodepletive chemotherapy

Lymphodepletive chemotherapy will be given to induce lymphopenia to facilitate engraftment and expansion of Anti-CD19 CAR-T cells. The lymphodepletive treatment will consist of cyclophosphamide and fludarabine:

- Cyclophosphamide 60 mg/kg will be infused intravenously over 60 minutes on day -6.
- Fludarabine 25 mg/m^2 will be infused intravenously daily over 30 minutes on days -5 to -3.
- NOTE: In cases when the CAR-T manufacture process is required to be extended from 8 to 12 days, as detected on day 8 onwards, it is possible that the days of chemotherapy will be modified accordingly.

Antiemetic premedications prior to conditioning chemotherapy include 5-HT_3 receptor antagonists and corticosteroids prior to cyclophosphamide (day -6) and 5-HT_3 receptor antagonists prior to fludarabine (day -5 to -3)

For the reduced cyclophosphamide dose group the treatment will consist of 3 days of immunosuppressive therapy. Cyclophosphamide given at a dose of 500mg/m^2/IV on day -5 to -3 and Fludarabine 30mg/m^2/IV on days -5 to -3, inclusive.

6.3.1 Therapy for symptom control or debulking prior to lymphodepletion

If treatment is required for control of disease – related symptoms or to decrease tumor – related bulk, patients may receive corticosteroids (up to 7 days prior to infusion of CAR-T cells) at a maximum daily dose of 200mg of prednisone or equivalent steroid dose. Use of other cytotoxic agents as bridging therapy for symptom control or debulking is not permitted in this protocol.

6.4 Study drug

6.4.1 Description

Anti-CD19 CAR-T cells are autologous T cells, engineered to express an extracellular single chain antibody (scFv) with specificity for CD19 linked to the intracellular signaling domains of the 4-1BB and TCRζ. Anti-CD19 CAR-T cells can be administered fresh (once release criteria for fresh infusion are met) or can be cryopreserved in infusible cryomedia for a later infusion. Anti-CD19 CAR-T cells can be administered in either a syringe (fresh) or 1 to 2 bags (fresh or frozen). Syringe/bag aliquoting, cell content and cryomedia composition will be done according to specifications of IND.

6.4.2 Preparation of anti-CD19 CAR-T cells for infusion

The anti-CD19 CAR-T cells are manufactured in the UHSCC-CTL as described in the associated IND and are not released from this facility until release criteria for the infused cells (e.g., cell purity, sterility, CAR-T cell dose, etc.) are met. Upon release, the cells are transported to the Stem Cell Transplant Unit of University Hospitals Seidman Cancer Center. An example of the release and infusion forms is attached in the Appendix 2.

6.4.3 Cell thawing (for frozen Anti-CD19 CAR-T cells)

If cells are frozen they will be thawed at the bedside, one bag at a time, using a water bath maintained at 37° C ± 2° C. The bag will be gently massaged until the cells are thawed. If the anti-CD19 CAR-T cell product appears to have a damaged or leaking bag or otherwise appears compromised, it should not be infused and should be returned to UHSCC-CTL.

6.4.4 Premedication

It is recommended that subjects be pre-medicated with acetaminophen 650 mg by mouth and diphenhydramine hydrochloride 25-50 mg by mouth or IV, prior to the infusion of anti-CD19 CAR-T cells. These medications may be repeated every six hours as needed. Pre-medication with tocilizumab is permitted per institutional practices. A course of non-steroidal anti-inflammatory medication may be prescribed if the patient continues to have fever not relieved by acetaminophen. **It is recommended that patients not receive systemic corticosteroids such as hydrocortisone, prednisone, prednisolone (Solu-Medrol) or dexamethasone (Decadron) at any time, except in the case of a life-threatening emergency, since this may have an adverse effect on T cells.** If corticosteroids are required for an acute life-threatening infusional reaction, an initial dose of hydrocortisone 100 mg is recommended.

6.4.5 Administration

The anti-CD19 CAR-T cells will be administered by syringe (fresh) or rapid intravenous infusion at a flow rate of approximately 5 mL (± 5 mL) per minute (fresh or frozen). Fresh products will be injected according to institutional standards. Frozen products will be thawed at the bedside and infused per institutional standards. One or two bags of anti-CD19 CAR-T cells will be infused. Each product will have a label, affixed or attached, containing the following: “FOR AUTOLOGOUS USE ONLY.” In addition, the label will have at least two unique identifiers such as the subject’s initials, birth date, study number and product DIN. Prior to the infusion, two individuals will independently verify this information in the presence of the subject and so confirm that the information is correctly matched to the participant.

Emergency medical equipment (i.e., emergency trolley) will be available during the infusion in case the subject has an allergic response, or severe hypotensive crisis, or any other reaction to the infusion. Vital signs (temperature, respiration rate, pulse, and blood pressure) will be taken before and after infusion, then every 15 minutes for at least one hour and until these signs are satisfactory and stable.

6.5 Cytokine release syndrome.

Cytokine release syndrome (CRS) is a potential risk of the infusion of anti-CD19 CAR-T cells. The majority of cases of CRS will occur within the first 48-72 hours of infusion, but can occur later [18].

Signs and symptoms associated with CRS include

- Constitutional: Fever ± rigors, malaise, fatigue, anorexia, myalgias, arthralgias, nausea, vomiting, headache;
- Skin: Rash;
- Gastrointestinal: Nausea, vomiting, diarrhea;
- Respiratory: Tachypnea, hypoxemia;
- Cardiovascular: Tachycardia, widened pulse pressure, hypotension, increased, cardiac output (early), potentially diminished cardiac output (late);
- Coagulation: Elevated D-dimer, hypofibrinogenemia, bleeding;
- Renal: Azotemia;
- Hepatic: Transaminitis, hyperbilirubinemia;
- Neurologic: Headache, mental status changes, confusion, delirium, word finding difficulty or frank aphasia, hallucinations, tremor, dysmetria, altered gait, seizures.

In this study, the signs and symptoms of CRS outlined above will be graded according to the revised grading criteria published by Lee and colleagues [18,19] (Table 6.5.1 and 6.5.2)

**Table 6.5.1 Revised CRS grading criteria**	
Grade 1	Symptoms are not life threatening and require symptomatic treatment only, (e.g., fever, nausea, fatigue, headache, myalgias, malaise)
Grade 2	Symptoms require and respond to moderate intervention<br>· Oxygen requirement <40% or<br>· Hypotension responsive to fluids or low dose of one vasopressor or<br>· Grade 2 organ toxicity
Grade 3	Symptoms require and respond to aggressive intervention<br>· Oxygen requirement ≥40% or<br>· Hypotension requiring high dose* or multiple vasopressors or<br>· Grade 3 organ toxicity or grade 4 transaminitis
Grade 4	· Life-threatening symptoms<br>· Requirement for ventilator support or<br>· Grade 4 organ toxicity (excluding transaminitis)
Grade 5	Death
Grade 2 -4 refer to CTCAE 5.0 grading<br>*Vasopressor guidelines listed in table 6.5.2	

**Table 6.5.2 High dose vasopressors (all doses required for ≥ 3 hours)**
Norepinephrine monotherapy: ≥20 µg/min
Dopamine monotherapy: ≥10 µg/kg/min
Phenylephrine monotherapy: ≥200 µg/min
Epinephrine monotherapy ≥10 µg/min
If on vasopressin: Vasopressin + norepinephrine equivalent of ≥10 µg/min*
If on combination vasopressors (not vasopressin) Norepinephrine equivalent of ≥20 µg/min*
* *VASST Trial vasopressor equivalent equation: norepinephrine equivalent dose = [norepinephrine (µg/min)] + [dopamine (µg/kg/min) ÷ 2] + [epinephrine (µg/min)] [phenylephrine (µg/min) ÷10].

CRS management algorithm

Management of CRS will be according to the Stem Cell Transplant Clinical Program SOP for management of adverse events following immune effector cell therapy (Appendix 3). This management plan is summarized in the algorithm delineated in figure 6.5

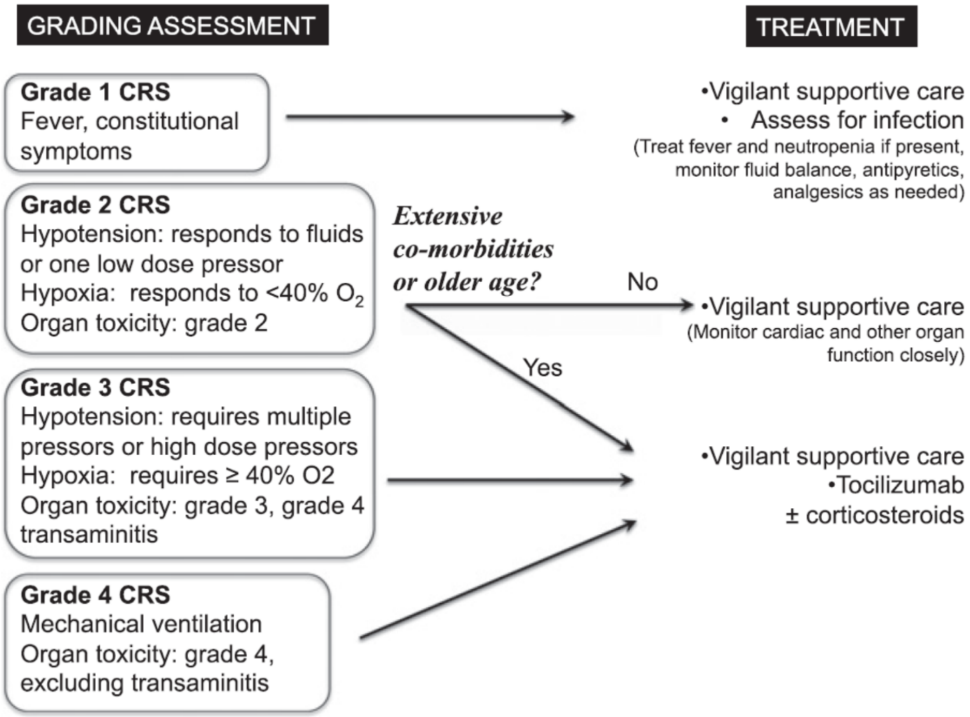

Figure 6.5. CRS treatment algorithm [15]

Use of additional agents, including siltuximab, or anakinra is permitted in cases without CRS improvement after tocilizumab.
No investigational or commercial agents or therapies other than those described below may be administered with the intent to treat the subject's malignancy.

6.6 Neurotoxicity (CAR-T cell – related encephalopathy syndrome)
Patients treated in previous clinical trials of anti-CD19 CAR-T cells have presented varying degrees of neurotoxicity [15,16]. Neelapu and colleagues reported a median onset to neurologic symptoms of 5 days, with 28% presenting grade 3 and higher toxicity [15]. Early events included dysphasia, inattention, calculation defects and handwriting difficulty. Most common events included encephalopathy (21%) aphasia (9%) and confusion (9%).

Management of Neurologic Toxicities:

- Subjects should be monitored daily for the first 7 days. Assessment with CARTOX-10 should be done daily (Appendix 6).
- Early events include changes in hand-writing, calculation defects, dysphasia

and inattention.

- If neurologic symptoms are present, consider CRES, consider other possible causes.
- If Grade ≥2 or higher neurologic toxicities, subjects should be monitored with continuous cardiac telemetry and pulse oximetry.
- Subjects with severe (Grade ≥3) neurologic symptoms should be considered for transfer to intensive care unit.
- Non – sedating, anti-seizure medications (i.e. levetiracetam) should be considered for any Grade ≥2 neurologic toxicity.

Management guidelines are present in table 6.6, additional management guidelines are available in the University Hospitals Seidman Cancer Center Standard Operating Procedure B7.41 Management of Patients Receiving Immune Effector Cell Therapy Appendix 3.

Table 6.6. General management guidelines for CAR-T associated encephalopathy (CRES)		
Neurologic event (CTCAE 5.0 Grade)	Concurrent CRS	No concurrent CRS
Grade 1	Manage per CRS guideline	Monitor neurologic symptoms daily
• Grade 2<br>• Examples:<br>Moderate somnolence, limiting instrumental ADLs<br>Moderate confusion<br>Encephalopathy limiting instrumental ADLs<br>Dysphasia<br>Seizures	• Tocilizumab per CRS guideline<br>• If no improvement within 24 hours of tocilizumab, administer dexamethasone 10mg intravenously every 6 hours (if no other steroids are started).<br>• Continue dexamethasone until event grade ≤1 and then taper over 3 days.<br>• Consider non-sedating antiseizure medications.	• Dexamethasone, 10mg intravenously every 6 hours.<br>• Continue dexamethasone until event grade ≤1 and then taper over 3 days.<br>• Consider non-sedating antiseizure medications.
• Grade 3<br>Examples: Somnolence, confusion, severe disorientation, encephalopathy, severe dysphasia.	• Tocilizumab per CRS guidelines<br>• Methylprednisolone 1000mg intravenously daily (at same time as tocilizumab); continue for 2 additional days, if improves, change to dexamethasone and manage as grade 2.<br>• Consider non-sedating antiseizure medications.	• Methylprednisolone 1000mg intravenously daily for 3 days, if improves, then change to dexamethasone and manage as grade 2.<br>• Consider non-sedating antiseizure medications.
Grade 4<br>Life threatening consequences. Urgent intervention needed. Requirement for mechanical ventilation. Rule out cerebral edema.	• Tocilizumab per CRS guidelines<br>• Methylprednisolone 1000mg intravenously daily (at same time as tocilizumab); continue for 2 additional days, if improves, change to dexamethasone and manage as grade 2.<br>Consider non-sedating antiseizure medications.	• Methylprednisolone 1000mg intravenously daily for 3 days, if improves, then change to dexamethasone and manage as grade 2 and 3.<br>• Consider non-sedating antiseizure medications.

6.7 Phase I Dose Escalation
Dose escalation will proceed within each cohort according to the following schema. Dose-limiting toxicity (DLT) is defined in section 6.8.

**Number of Subjects with DLT at a Given Dose Level**	**Escalation Decision Rule**
0 out of 3	Enter 3 subjects at the next dose level.
1 out of 3	Enter 3 more subjects at this dose level.<br>● If 0 of these 3 subjects experience DLT, proceed to the next dose level.<br>● If 1 or more of this group suffer DLT, then dose escalation is stopped, and this dose is declared the maximally administered dose. Three (3) additional subjects will be entered at the next lowest dose level if only 3 subjects were treated previously at that dose. In case this situation occurs at dose level 1, subsequent subjects will be dosed at level -1.
≥2	Dose escalation will be stopped. This dose level will be declared the maximally administered dose (highest dose administered). Three (3) additional subjects will be entered at the next lowest dose level if only 3 subjects were treated previously at that dose. In case this situation occurs at dose level 1, subsequent subjects will be dosed at level -1.
≤1 out of 6 at highest dose level below the maximally administered dose	This is generally the recommended maximally tolerated dose. At least 6 subjects should be entered at the recommended phase 2 dose.

6.8 Definition of Dose-Limiting Toxicity
Management of common toxicities and dose modifications are outlined in section 7.0.

In this study, a dose limiting toxicity (DLT) includes those adverse events graded according to NCI Common Terminology Criteria for Adverse Events (CTCAE) version 5.0, with the exception of cytokine release syndrome to be graded using criteria provided in Sec. 6.4. To be DLTs, adverse events must be suspected to be secondary to CAR-T cell infusion, occur during the first 30 days after infusion and meet the following criteria:

1. Grade ≥ 3 non-hematologic toxicities, with the following exceptions
   - Laboratory abnormalities without associated symptomatology or clinical consequence that resolve in less than 7 days;
   - Toxicities associated with Grade ≤ 2 cytokine release syndrome (graded per criteria provided in Sec. 6.4);

- Toxicities associated with grade 3 cytokine release syndrome that improves to grade ≤ 2 within 3 days of intervention;
- Laboratory abnormalities compatible with tumor lysis syndrome;
- Grade 3 neurotoxicity that resolves to grade 1 or less within 3 days (with or without intervention).

2. Grade ≥ 4 hematologic toxicities that persist at a grade ≥ 3 despite maximum supportive care for >21 days.

6.9 General Concomitant Medications and Supportive Care Guidelines
Subjects should receive full supportive care, including transfusions of blood and blood products, cytokines, antibiotics, antiemetics, etc when appropriate.

6.10 Criteria for Removal from Study
Treatment and follow up in this protocol may continue until one of the following criteria applies:

- Disease progression, treatment failure or recurrence at any time;
- Intercurrent illness that prevents infusion or follow up post-infusion;
- Pregnancy during the course of the study for a child-bearing participant
- The investigator considers it, for safety reasons, to be in the best interest of the patient;
- General or specific changes in the patient's condition render the patient unacceptable for treatment (before infusion) or follow-up (after infusion) in the judgment of the investigator,
- Patient decision to withdraw prior to infusion or from post-infusion follow-up,
- Death

The Sponsor reserves the right to temporarily suspend or prematurely discontinue this study. The date and reason for discontinuation must be documented. Every effort should be made to complete the appropriate assessments

NOTE: For subjects who discontinue their participation in the trial after CAR-T cell infusion, long-term follow up for monitoring of potential gene therapy-related delayed adverse events will continue as required per federal guidelines and as delineated in section 3.4.2. In patients who discontinue their participation after CAR-T cell infusion for reasons other than death or decision to withdraw from the study, correlative samples will continue to be collected as scheduled.
.
6.11 Duration of Follow Up
Subjects will be followed for toxicity for 30 days after treatment has been discontinued or until death, whichever occurs first.

The clinical course of each adverse event will be followed until resolution, stabilization, or

until it has been determined that the study treatment or participation is not the cause.

Serious adverse events that are still ongoing at the end of the study period will necessitate follow-up to determine the final outcome. Any serious adverse event that occurs after the study period and is considered to be possibly related to the study treatment or study participation will be recorded and reported immediately.

Follow up will be 15 years for monitoring for potential gene therapy – related delayed adverse events.

Subjects will be monitored for replication competent lentivirus (RCL), based on FDA recommendations, by analysis of peripheral blood mononuclear cells by quantitative PCR for the envelope gene sequence VSV-G. Blood samples will be obtained at pretreatment and post CART infusion at 3, 6, 12 months and yearly up to 15 years. If post infusion samples are negative for the first 12 months, yearly review of medical history will suffice and follow up samples can be discontinued.

During the first 5 years after treatment with anti-CD19 CAR-T cells, subject follow up will be done annually in clinical visits, in person, following the form delineated in Appendix 4, which will record the following information:

- Exposure to mutagenic agents;
- Exposure to medicinal products;
- History;
- Physical examination;
- Laboratory test results including assay for persistent vector sequence;
- Emergence of new conditions:
    - New malignancy(ies)
    - New incidence or exacerbation of a pre-existing neurologic disorder
    - New incidence or exacerbation of a pre-existing rheumatologic or autoimmune disorder
    - New incidence of a hematologic disorder.

During the subsequent 10 years (years 6 – 15 after treatment with anti-CD19 CAR-T cells), subjects will be contacted annually. If office visits are not scheduled, then patients may be contacted by telephone or written questionnaire. Contact throughout years 6 – 15 will be done following the form in Appendix 5, which will record the following information:

- Emergence of new conditions, including but not limited to:
    - New malignancy(ies)
    - New incidence or exacerbation of a pre-existing neurologic disorder
    - New incidence or exacerbation of a pre-existing rheumatologic or autoimmune disorder
    - New incidence of a hematologic disorder.

During years 6 – 15 after treatment, subjects with previous laboratory results

indicating vector persistence will have this test repeated annually until resolution.

All subjects will be provided with the study coordinator contact information and encouraged to report delayed adverse events, including unexpected illness or hospitalization.

6.12 Retreatment

Retreatment has been allowed in previous anti-CD19 CAR-T clinical trials and has resulted in clinical responses[15].

A second infusion may be offered to subjects who achieve a partial or complete response and subsequently relapse.

Retreatment within this protocol will only be done on a case-by-case basis and after discussion with the Principal Investigator (Caimi), the IND Sponsor (de Lima) and with prior communication with the Food and Drug Administration (FDA) for review and comment of the treatment plan of the subject to be retreated. This communication with the FDA will need to include the following:

1. Justification for retreatment;
2. Disease status;
3. Anti-CD19 dose changes from first treatment;
4. Summary of adverse events, and duration of these adverse events following the initial dose

Subjects will be re-consented, including a discussion regarding benefits and risks of a second infusion. Subjects undergoing retreatment may need to undergo leukapheresis a second time for the manufacturing of anti-CD19 CAR-T cells if sufficient residual CAR-T cells are not available from the initial procedure.

To be eligible for a second course of treatment:

- Subject must have presented at least a partial response after initial treatment with anti-CD19 CAR-T cell therapy.
- Subjects must be re-evaluated and continue to meet the original study eligibility criteria.
- Subjects should not have received additional therapy for the treatment of lymphoma.
- All clinically significant toxicity related to the conditioning regimen and first anti-CD19 CAR-T cell infusion (with the exception of alopecia) should be resolved to grade ≤1.
- At least 30 days from the initial dose of CAR-T cells must have elapsed.

Subjects who are retreated will follow the same treatment schedule and procedural requirements per the initial treatment. The treatment dose of anti-CD19 CAR-T cells to be infused will correspond to that of the latest safe dose level (i.e. the dose level that was last

completed prior to dose escalation if the trial is still ongoing or the dose level considered to be the maximum tolerated dose if the study is completed). Subjects who experience a DLT during initial CAR-T treatment will not be eligible for retreatment.

The last scan prior to retreatment will be considered the baseline for the purpose of response evaluation of retreatment. Repetition of scans will not be necessary prior to enrollment if these have been performed within 8 weeks. Other enrollment and organ function assessments will need to be repeated prior to retreatment.

A maximum of 1 retreatment course will be permitted per subject.

6.13 Prohibited medications

Treatment with anti-CD19 CAR-T cell depends on the collection and persistence of functional infused cells. Because of this, medications that may be lymphotoxic should be avoided in certain periods (**unless required for management of the underlying disease or toxicities of the anti-CD19 CAR-T**). Agents to avoid include:

- Corticosteroids;
- Lymphodepletive agents including alemtuzumab and antithymocyte globulin;
- Immunosupressants.

These agents should be avoided 10 days prior to peripheral blood collection, 5 days prior to anti-CD19 CAR-T cell infusion and for 90 days after infusion.

7 Dose Delays/Dose Modifications

7.1 Cyclophosphamide

There are no planned dose modifications for cyclophosphamide.

7.2 Fludarabine

Fludarabine dose will be modified for patients who present a creatinine clearance (CrCl) of less than 50ml/min, calculated by the Cockroft – Gault formula.

- If Creatinine Clearance is >50ml/min, then no fludarabine dose adjustment is necessary.
- If Creatinine Clearance is between 30 and 50ml/min, then fludarabine should be given at 75% of the calculated dose (25% dose reduction), on days -5 to -3.
- If Creatinine is less than 30ml/min, then use of fludarabine is contraindicated and the patient will be excluded from the trial.

Once fludarabine treatment has been initiated as adjusted above, there will be no further dose modifications.

7.3 Anti-CD19 CAR-T cells

There are no planned dose modifications for the CAR-T cell infusion.

8 Adverse Events and Potential Risks

The following is a list of AEs (Section 8.1) and the reporting requirements associated with observed AEs (Sections 8.3 and 8.4).

The clinical course of each event will be followed until resolution, stabilization, or until it has been determined that the study treatment or participation is not the cause.

Serious adverse events that are still ongoing at the end of the study period will necessitate follow-up to determine the final outcome. Any serious adverse event that occurs after the study period and is considered to be possibly related to the study treatment or study participation will be recorded and reported immediately (within approximately 24 hours of acknowledgement).

Please refer to the package insert(s) for the comprehensive list of adverse events. Many of these toxicities are recognized toxicities of transplantation and will not be considered adverse effects in the context of this study. For example, re-admissions are common after transplantation, and reasons for re-admissions most commonly include fever with or without infection. Unless inpatient hospitalizations are thought to be due to one of the study medications, they will not be reported as serious adverse events.

8.1 Adverse Events

8.1.1 Chimeric Antigen Receptor T (CAR-T) cells

Anti-CD19 CAR-T cells have presented two main toxicities: cytokine release syndrome and neurotoxicity.

Cytokine release syndrome (CRS) occurs usually in the first 2 weeks after CAR-T cell infusion and is manifested by the following spectrum of findings:

- fever;
- malaise and constitutional symptoms;
- hypotension;
- capillary leak, edema, pleural effusion or pulmonary congestion;
- hypoxemia;
- coagulopathy secondary to disseminated intravascular coagulation;
- end organ dysfunction, including respiratory failure, cardiovascular impairment, renal insufficiency;

Management of cytokine release syndrome includes early detection through frequent monitoring, intensive supportive care, including intensive care unit admission, monitoring of C reactive protein throughout the course of the reaction, and administration of tocilizumab, a humanized monoclonal antibody directed against interleukin 6 receptor, a cytokine that plays a central role in inflammatory and immune reactions.

Neurotoxicity occurs independently of CRS, although usually at a slightly later time point.

The pathogenesis of neurotoxicity is not well understood. The manifestations include:

- delirium,
- speech disturbances;
- focal neurologic deficits;
- fine motor impairment;
- seizures;
- coma (rare).

The management of neurotoxicity includes frequent monitoring of the neurologic status of the patient and their fine motor skills; once sustained or > Grade 2 neurotoxicity is observed, corticosteroids (primarily dexamethasone) are used for treatment of this adverse event.

Although rare, there are descriptions of cases of cerebral edema associated with CAR-T infusions. This latter reaction is idiosyncratic and there are no predictive parameters identified so far.

The modified T cells target a structure called CD19, which is shared by cancer cells and some normal cells in the body. The normal cells with CD19 make proteins called antibodies, which help fight and prevent infections. A potential risk of this treatment is that the body won't be able to make antibodies against infections as the normal cells with CD19 did before this treatment.

The majority of studies conducted with Anti-CD19 CAR-T cells for treatment of NHL have utilized some form of lymphodepletion to facilitate CAR-T cell persistence. The lymphodepletive chemotherapy with Fludarabine and Cyclophosphamide is expected to cause pancytopenia.

8.1.2 Fludarabine
The most common adverse events include myelosuppression (neutropenia, thrombocytopenia and anemia), fever and chills, infection, and nausea and vomiting. Other commonly reported events include malaise, fatigue, anorexia, and weakness. Serious opportunistic infections have occurred in CLL patients treated with fludarabine. Adverse events and those reactions that are more clearly related to the drug are arranged below according to body system.

Hematopoietic Systems
Hematologic events (neutropenia, thrombocytopenia, and/or anemia) were reported in the majority of CLL patients treated with fludarabine. During fludarabine treatment of 133 patients with CLL, the absolute neutrophil count decreased to less than 500/mm3 in 59% of patients, hemoglobin decreased from pretreatment values by at least 2 grams percent in 60%, and platelet count decreased from pretreatment values by at least 50% in 55%. Myelosuppression may be severe, cumulative, and may affect multiple cell lines. Bone marrow fibrosis occurred in one CLL patient treated with fludarabine.

Several instances of trilineage bone marrow hypoplasia or aplasia resulting in pancytopenia, sometimes resulting in death, have been reported in postmarketing surveillance. The duration of clinically significant cytopenia in the reported cases has ranged from approximately 2 months to approximately 1 year. These episodes have occurred both in previously treated or untreated patients.

Life-threatening and sometimes fatal autoimmune phenomena such as hemolytic anemia, autoimmune thrombocytopenia/ thrombocytopenic purpura (ITP), Evans syndrome, and acquired hemophilia have been reported to occur in patients receiving fludarabine. The majority of patients rechallenged with fludarabine developed a recurrence in the hemolytic process.

In postmarketing experience, cases of myelodysplastic syndrome and acute myeloid leukemia, mainly associated with prior, concomitant or subsequent treatment with alkylating agents, topoisomerase inhibitors, or irradiation have been reported.

Infections
Serious and sometimes fatal infections, including opportunistic infections and reactivations of latent viral infections such as VZV (Herpes zoster), Epstein-Barr virus and JC virus (progressive multifocal leukoencephalopathy) have been reported in patients treated with fludarabine.

Rare cases of Epstein Barr Virus (EBV) associated lymphoproliferative disorders have been reported in patients treated with fludarabine.

Metabolic
Tumor lysis syndrome has been reported in CLL patients treated with fludarabine. This complication may include hyperuricemia, hyperphosphatemia, hypocalcemia, metabolic acidosis, hyperkalemia, hematuria, urate crystalluria, and renal failure. The onset of this syndrome may be heralded by flank pain and hematuria.

Nervous System
Objective weakness, agitation, confusion, seizures, visual disturbances, optic neuritis, optic neuropathy, blindness and coma have occurred in CLL patients treated with fludarabine at the recommended dose. Peripheral neuropathy has been observed in patients treated with fludarabine and one case of wrist-drop was reported.

In postmarketing experience, cases of progressive multifocal leukoencephalopathy have been reported. Most cases had a fatal outcome. Many of these cases were confounded by prior and/or concurrent chemotherapy. The time to onset has ranged from a few weeks to approximately one year after initiating treatment.

Impairment of Fertility
Studies in mice, rats and dogs have demonstrated dose-related adverse effects on the male

reproductive system. Observations consisted of a decrease in mean testicular weights in mice and rats with a trend toward decreased testicular weights in dogs and degeneration and necrosis of spermatogenic epithelium of the testes in mice, rats and dogs. The possible adverse effects on fertility in humans have not been adequately evaluated.

Pulmonary System

Pneumonia, a frequent manifestation of infection in CLL patients, occurred in 16%, and 22% of those treated with fludarabine in the MDAH and SWOG studies, respectively. Pulmonary hypersensitivity reactions to fludarabine characterized by dyspnea, cough and interstitial pulmonary infiltrate have been observed.

In postmarketing experience, cases of severe pulmonary toxicity have been observed with fludarabine use which resulted in ARDS, respiratory distress, pulmonary hemorrhage, pulmonary fibrosis, and respiratory failure. After an infectious origin has been excluded, some patients experienced symptom improvement with corticosteroids.

Gastrointestinal System

Gastrointestinal disturbances such as nausea and vomiting, anorexia, diarrhea, stomatitis and gastrointestinal bleeding have been reported in patients treated with fludarabine.

Cardiovascular

Edema has been frequently reported. One patient developed a pericardial effusion possibly related to treatment with fludarabine. No other severe cardiovascular events were considered to be drug related.

Genitourinary System

Rare cases of hemorrhagic cystitis have been reported in patients treated with fludarabine.

Skin

Skin toxicity, consisting primarily of skin rashes, has been reported in patients treated with fludarabine.

Erythema multiforme, Stevens-Johnson syndrome, toxic epidermal necrolysis, and pemphigus have been reported, with fatal outcomes in some cases. Worsening or flare up of pre-existing skin cancer lesions, as well as new onset of skin cancer, has been reported in patients during or after treatment with fludarabine.

Data in the following table are derived from the 133 patients with CLL who received fludarabine in the MDAH and SWOG studies:

Table 8.1.2. Adverse events of fludarabine		
Adverse Event	MDAH (n = 101) (%)	SWOG (n = 32) (%)
Any adverse event	88	92
Body as a whole	72	84
Fever	60	69
Chills	11	19
Fatigue	10	38
Infection	33	44
Pain	20	22
Malaise	8	6
Diaphoresis	1	13
Alopecia	0	3
Anaphylaxis	1	0
Hemorrhage	1	0
Hyperglycemia	1	6
Dehydration	1	0
Neurological	21	69
Weakness	9	65
Paresthesia	4	12
Headache	3	0
Visual disturbance	3	15
Hearing loss	2	6
Sleep disorder	1	3
Depression	1	0
Cerebellar syndrome	1	0
Impaired mentation	1	0
Pulmonary	35	69
Cough	10	44
Pneumonia	16	22
Dyspnea	9	22
Sinusitis	5	0

Pharyngitis	0	9
Upper respiratory infection	2	16
Allergic pneumonitis	0	6
Epistaxis	1	0
Hemoptysis	1	6
Bronchitis	1	0
Hypoxia	1	0
Gastrointestinal	46	63
Nausea/vomiting	36	31
Diarrhea	15	13
Anorexia	7	34
Stomatitis	9	0
Gastrointestinal bleeding	3	13
Esophagitis	3	0
Mucositis	2	0
Liver failure	1	0
Abnormal liver function tests	1	3
Cholelithiasis	0	3
Constipation	1	3
Dysphagia	1	0
Cutaneous	17	18
Rash	15	15
Pruritus	1	3
Seborrhea	1	0
Genitourinary	12	22
Dysuria	4	2
Urinary infections	2	15
Hematuria	2	3
Renal failure	1	0
Abnormal renal function tests	1	0
Proteinuria	1	0

Hesitancy	0	3
Cardiovascular	12	38
Edema	8	19
Angina	0	6
Congestive heart failure	0	3
Arrhythmia	0	3
Supraventricular tachycardia	0	3
Myocardial infarction	0	3
Deep venous thrombosis	1	3
Phlebitis	1	3
Transient ischemic attack	1	0
Aneurysm	1	0
Cerebrovascular accident	0	3
Musculoskeletal	7	16
Myalgia	4	16
Osteoporosis	2	0
Arthralgia	1	0
Tumor lysis syndrome	1	0

Thousands of adult patients received fludarabine in studies of other leukemias, lymphomas, and other solid tumors. The spectrum of adverse effects reported in these studies was consistent with the data presented above.

8.1.3 Cyclophosphamide

Hematopoietic system: Neutropenia occurs in patients treated with cyclophosphamide. The degree of neutropenia is particularly important because it correlates with a reduction in resistance to infections. Fever without documented infection has been reported in neutropenic patients.

Gastrointestinal system: Nausea and vomiting occur with cyclophosphamide therapy. Anorexia and, less frequently, abdominal discomfort or pain and diarrhea may occur. There are isolated reports of hemorrhagic colitis, oral mucosal ulceration and jaundice occurring during therapy.

Skin and its structures: Alopecia occurs in patients treated with cyclophosphamide. Skin rash occurs occasionally in patients receiving the drug. Pigmentation of the skin and changes in nails can occur.

The following adverse reactions have been identified from clinical trials or post-marketing surveillance. Because they are reported from a population from unknown size, precise estimates of frequency cannot be made.

Cardiac: cardiac arrest, ventricular fibrillation, ventricular tachycardia, cardiogenic shock, pericardial effusion (progressing to cardiac tamponade), myocardial hemorrhage, myocardial infarction, cardiac failure (including fatal outcomes), cardiomyopathy, myocarditis, pericarditis, carditis, atrial fibrillation, supraventricular arrhythmia, ventricular arrhythmia, bradycardia, tachycardia, palpitations, QT prolongation.

Post Marketing Experience:

Congenital, Familial and Genetic: intra-uterine death, fetal malformation, fetal growth retardation, fetal toxicity (including myelosuppression, gastroenteritis).

Ear and Labyrinth: deafness, hearing impaired, tinnitus. Endocrine: water intoxication. Eye: visual impairment, conjunctivitis, lacrimation.

Gastrointestinal: gastrointestinal hemorrhage, acute pancreatitis, colitis, enteritis, cecitis, stomatitis, constipation, parotid gland inflammation.

General Disorders and Administration Site Conditions: multiorgan failure, general physical deterioration, influenza-like illness, injection/infusion site reactions (thrombosis, necrosis, phlebitis, inflammation, pain, swelling, erythema), pyrexia, edema, chest pain, mucosal inflammation, asthenia, pain, chills, fatigue, malaise, headache.

Hematologic: myelosuppression, bone marrow failure, disseminated intravascular coagulation and hemolytic uremic syndrome (with thrombotic microangiopathy).

Hepatic: veno-occlusive liver disease, cholestatic hepatitis, cytolytic hepatitis, hepatitis, cholestasis; hepatotoxicity with hepatic failure, hepatic encephalopathy, ascites, hepatomegaly, blood bilirubin increased, hepatic function abnormal, hepatic enzymes increased.

Immune: immunosuppression, anaphylactic shock and hypersensitivity reaction.

Infections: The following manifestations have been associated with myelosuppression and immunosuppression caused by cyclophosphamide: increased risk for and severity of pneumonias (including fatal outcomes), other bacterial, fungal, viral, protozoal and, parasitic infections; reactivation of latent infections, (including viral hepatitis, tuberculosis), Pneumocystis jiroveci, herpes zoster, Strongyloides, sepsis and septic shock.

Investigations: blood lactate dehydrogenase increased, C-reactive protein increased.

Metabolism and Nutrition: hyponatremia, fluid retention, blood glucose increased, blood

glucose decreased.

Musculoskeletal and Connective Tissue: rhabdomyolysis, scleroderma, muscle spasms, myalgia, arthralgia.

Neoplasms: acute leukemia, myelodysplastic syndrome, lymphoma, sarcomas, renal cell carcinoma, renal pelvis cancer, bladder cancer, ureteric cancer, thyroid cancer.

Nervous System: encephalopathy, convulsion, dizziness, neurotoxicity has been reported and manifested as reversible posterior leukoencephalopathy syndrome, myelopathy, peripheral neuropathy, polyneuropathy, neuralgia, dysesthesia, hypoesthesia, paresthesia, tremor, dysgeusia, hypogeusia, parosmia.

Pregnancy: premature labor.

Psychiatric: confusional state.

Renal and Urinary: renal failure, renal tubular disorder, renal impairment, nephropathy toxic, hemorrhagic cystitis, bladder necrosis, cystitis ulcerative, bladder contracture, hematuria, nephrogenic diabetes insipidus, atypical urinary bladder epithelial cells.

Reproductive System: infertility, ovarian failure, ovarian disorder, amenorrhea, oligomenorrhea, testicular atrophy, azoospermia, oligospermia.

Respiratory: pulmonary veno-occlusive disease, acute respiratory distress syndrome, interstitial lung disease as manifested by respiratory failure (including fatal outcomes), obliterative bronchiolitis, organizing pneumonia, alveolitis allergic, pneumonitis, pulmonary hemorrhage; respiratory distress, pulmonary hypertension, pulmonary edema, pleural effusion, bronchospasm, dyspnea, hypoxia, cough, nasal congestion, nasal discomfort, oropharyngeal pain, rhinorrhea.

Skin and Subcutaneous Tissue: toxic epidermal necrolysis, Stevens-Johnson syndrome, erythema multiforme, palmar-plantar erythrodysesthesia syndrome, radiation recall dermatitis, toxic skin eruption, urticaria, dermatitis, blister, pruritus, erythema, nail disorder, facial swelling, hyperhidrosis.

Tumor lysis syndrome: like other cytotoxic drugs, cyclophosphamide may induce tumor-lysis syndrome and hyperuricemia in patients with rapidly growing tumors.

Vascular: pulmonary embolism, venous thrombosis, vasculitis, peripheral ischemia, hypertension, hypotension, flushing, hot flush.

8.2 Definitions

8.2.1 Adverse Event

An **adverse event** (AE) is any unfavorable or unintended event, physical or psychological,

associated with a research study, which causes harm or injury to a research participant as a result of the participant's involvement in a research study. The event can include abnormal laboratory findings, symptoms, or disease associated with the research study. The event does not necessarily have to have a causal relationship with the research, any risk associated with the research, the research intervention, or the research assessments.

Adverse events may be the result of the interventions and interactions used in the research; the collection of identifiable private information in the research; an underlying disease, disorder, or condition of the subject; and/or other circumstances unrelated to the research or any underlying disease, disorder, or condition of the subject.

8.2.2 Serious Adverse Events

A **serious adverse event** (SAE) is any adverse experience occurring at any dose that results in any of the following outcomes:

- Results in **death.**
- Is a **life-threatening** adverse experience. The term life-threatening in the definition of serious refers to an adverse event in which the subject was at risk of death at the time of the event. It does not refer to an adverse event which hypothetically might have caused death if it were more severe.
- Requires **inpatient hospitalization or prolongation of existing hospitalization**. Any adverse event leading to hospitalization or prolongation of hospitalization will be considered as Serious, UNLESS at least one of the following expectations is met:
    - The admission results in a hospital stay of less than 24 hours OR
    - The admission is pre-planned (e.g., elective or scheduled surgery arranged prior to the start of the study) OR
    - The admission is not associated with an adverse event (e.g., social hospitalization for purposes of respite care.

  However, it should be noted that invasive treatment during any hospitalization may fulfill the criteria of "medically important" and as such may be reportable as a serious adverse event dependent on clinical judgment. In addition where local regulatory authorities specifically require a more stringent definition, the local regulation takes precedent.
- Results in **persistent or significant disability/incapacity**. The definition of disability is a substantial disruption of a person's ability to conduct normal life's functions.
- Is a **congenital anomaly/birth defect**.
- Is an **important medical event**. Important medical events that may not result death, be life-threatening, or require hospitalization may be considered a serious adverse experience when, based upon appropriate medical judgment, they may jeopardize the subject and may require medical or surgical intervention to prevent one of the outcomes listed in this definition. Examples of such medical

events include allergic bronchospasm requiring intensive treatment in an emergency room or at home, blood disease or disorders, or convulsions that do not result in inpatient hospitalization, or the development of drug dependency or drug abuse. The development of a new cancer is always considered an important medical event.

For the purpose of this study the following events would not be considered adverse events and would not be recorded in the database:

- Abnormal laboratory findings considered associated to the original disease.

8.2.3Adverse Event Evaluation

The investigator or designee is responsible for ensuring that all adverse events (both serious and non-serious) observed by the clinical team or reported by the subject which occur after the subject has signed the informed consent through 12 months after CAR-T cell infusion are fully recorded in the subject's medical records. Source documentation must be available to support all adverse events.

A laboratory test abnormality considered clinically relevant (e.g., causing the subject to withdraw from the study, requiring treatment or causing apparent clinical manifestations, result in a delay or dose modification of study treatment, or judged relevant by the investigator), should be reported as an adverse event.

The investigator or sub-investigator (treating physician if applicable) will provide the following for all adverse events (both serious and non-serious):

- Event term (as per CTCAE version 5.0)
- Description of the event
- Date of onset and resolution
- **Expectedness of the toxicity**
- **Grade of toxicity**
- **Attribution of relatedness to the investigational agent- (this must be assigned by an investigator, sub-investigator, or treating physician)**
- Action taken as a result of the event, including but not limited to; no changes, dose interrupted, reduced, discontinued, etc. or action taken with regard to the event, i.e. no action, received concomitant medication or other intervention, etc.
- Outcome of event

Descriptions and **grading scales** found in the NCI Common Terminology Criteria for Adverse Events (CTCAE) version **5.0** will be utilized for AE reporting, with the exception of grading provided in Sec. 6.4 for cytokine release syndrome.

An expected adverse event is an event previously known or anticipated to result from participation in the research study or any underlying disease, disorder, or condition of the subject. The event is usually listed in the Investigator Brochure, consent form or research protocol.

An unexpected adverse event is an adverse event not previously known or anticipated to result from the research study or any underlying disease, disorder, or condition of the subject.

Attribution is the relationship between an adverse event or serious adverse event and the study drug. Attribution will be assigned as follows:

- Definite – The AE is clearly related to the study drug.
- Probable – The AE is likely related to the study drug.
- Possible – The AE may be related to the study drug.
- Unlikely – The AE is doubtfully related to the study drug.
- Unrelated – The AE is clearly NOT related to the study drug.

Protocol must specify if attribution is required for individual components of the treatment regimen or the treatment regimen as a whole.

8.3 SAE Report Form

SAEs will be recorded on the FDA Form 3500A (MedWatch) but should only be reported as instructed below. The electronic FDA SAE reporting forms should not be used.

8.4 Reporting Procedures for Serious Adverse Events

For the purposes of safety reporting, all serious adverse events will be reported that occur from the start of the infusion of the first dose of lymphodepletion regimen through 30 days after the infusion of CAR-T cells. Adverse events, both serious and non-serious, and deaths that occur during this period will be recorded in the source documents. All SAEs should be monitored until they are resolved or are clearly determined to be due to a subject's stable or chronic condition or intercurrent illness(es). Related AEs will be followed until resolution to baseline or grade 1 or stabilization.

8.4.1 SAE Reporting Requirements

- Participating investigators (all sites) must report all serious adverse events to the Sponsor-Investigator within **24 hours** of discovery or notification of the event. The participating investigator must also provide follow-up information on the SAE until final resolution.
    - Paolo F. Caimi. Fax 216-844-1256, Phone 216-844-8220
- The Principal Investigator will review the SAE and report the event to the FDA, external collaborator(s), and IRB as applicable.
- It is the Sponsor-Investigator's responsibility to ensure that ALL serious adverse events that occur on the study (e.g. ALL SAEs that occur at each enrolling institution) are reported to all participating sites.

Institutional Review Board Reporting Requirements:

- Investigative sites will report adverse events to their respective IRB according to

the local IRB's policies and procedures in reporting adverse events.

FDA Reporting:

The University Hospitals Principal Investigator, as holder of the IND, will be responsible for all communication with the FDA. In accordance with 21 CFR 312.32, the University Hospitals Principal Investigator is responsible for notifying the FDA of SAEs that are serious, unexpected (not listed in the Investigator Brochure) and judged to be related (i.e., possible, probable, definite) to the study drug. Events meeting the following criteria need to be submitted to the FDA as Expedited IND Safety Reports.

7 Calendar Day IND Safety Report
Any unexpected fatal or life-threatening suspected adverse event represent especially important safety information and, therefore, must be reported more rapidly to FDA (21 CFR 312.32(c)(2)). Any unexpected fatal or life-threatening suspected adverse event must be reported to FDA no later than 7 calendar days after the University Hospitals Principal Investigator initial receipt of the information (21 CFR 312.32(c)(2)). University Hospitals Principal Investigator will complete a Medwatch Form FDA 3500A and notify the FDA by telephone or facsimile transmission.

15 Calendar Day IND Safety Report
The timeframe for submitting an IND safety report to FDA and all participating investigators is no later than 15 calendar days after the University Hospitals Principal Investigator determines that the suspected adverse event or other information qualifies for reporting (21 CFR 312.32(c)(1)). This includes any serious, unexpected adverse events considered reasonably or possibly related to the investigational agent. University Hospitals Principal Investigator will complete a Medwatch Form FDA 3500A and notify the FDA by telephone or facsimile transmission. If FDA requests any additional data or information, the University Hospitals Principal Investigator must submit it to FDA as soon as possible, but no later than 15 calendar days after receiving the request (21 CFR 312.32(c)(1)(v)).

Follow-up IND Safety Report
Any relevant additional information that the University Hospitals Principal Investigator obtains that pertains to a previously submitted IND safety report must be submitted to FDA as a Follow-up IND Safety Report without delay, as soon as the information is available (21 CFR 312.32(d)(2)). The University Hospitals Principal Investigator will maintain records of its efforts to obtain additional information.

Reporting Serious Problems to FDA
Medwatch Form FDA 3500A:
http://www.fda.gov/Safety/MedWatch/HowToReport/DownloadForms/default.htm
Telephone: 1-800-332-1088
Fax: 1-800-FDA-0178

8.5 SAEs and OnCore

- All SAEs will be entered into OnCore.
- A copy of the SAE form(s) submitted to the sponsor-investigator is also uploaded into OnCore.

8.6 Data Safety and Toxicity Committee

It is the responsibility of each site PI to ensure that ALL SAEs occurring on this trial (internal or external) are reported to the Case Comprehensive Cancer Center's Data and Safety Toxicity Committee. This submission is simultaneous with their submission to the sponsor and/or other regulatory bodies.

The sponsor-investigator is responsible for submitting an annual report to the DSTC as per CCCC Data and Safety Monitoring Plan.

8.7 Data and Safety Monitoring Plan (DSMP)

This protocol will adhere to the policies of the Case Comprehensive Cancer Center Data and Safety Monitoring Plan in accordance with NCI guidelines.

9 PHARMACEUTICAL INFORMATION

A list of the adverse events and potential risks associated with the investigational or commercial agents administered in this study can be found in Section 8.1.

9.1 Investigational Agents

9.1.1 Anti-CD19 Chimeric Antigen Receptor T (CAR-T) Cells

Solution preparation: The anti-CD19 CAR-T cells are prepared at the UHSCC Cellular Therapy Laboratory (CTL). Once manufacturing is complete and release criteria are met, anti-CD19 CAR-T cells can be released for immediate infusion or frozen, stored in cryomedia for infusion at a later date.

Storage requirements: When frozen, Anti-CD19 CAR-T cell products will be stored at the UHSCC-CTL in the vapor phase of liquid nitrogen.

Route of administration: Short intravenous infusion over 5 – 30 minutes.

Drug Procurement: The investigational agent will be manufactured at University Hospitals Seidman Cancer Center Cellular Therapy Laboratory.

Packaging: The anti-CD19 CAR-T cell product will be packaged in syringes or bags with an approximate content of 10-60 mL (syringe) or 25-80 mL (per bag), packed for transport in an outer protective package.

Labeling: Syringes/Bags containing the anti-CD19 CAR-T cell product will be labeled in accordance with applicable regulatory guidelines.

9.2 Commercial Agent

9.2.1 Cyclophosphamide

**Chemical Name**	Cyclophosphamide
**Other Names**	Cytoxan ®, Endoxan®, Neosar®, Procytox®, Revimmune®, Cycloblastin®
**Classification**	Alkylating agent
**Molecular Formula**	$C_7H_{15}Cl_2N_2O_2P$
**Mode of action**	The mechanism of action is thought to involve cross-linking of tumor cell DNA.
**Metabolism**	The liver is the major site of cyclophosphamide activation. Approximately 75% of the administered dose of cyclophosphamide is activated by hepatic microsomal cytochrome P450s including CYP2A6, 2B6, 3A4, 3A5, 2C9, 2C18 and 2C19, with 2B6 displaying the highest 4-hydroxylase activity. Cyclophosphamide is activated to form 4-hydroxycyclophosphamide, which is in equilibrium with its ring-open tautomer aldophosphamide. 4-hydroxycyclophosphamide and aldophosphamide can undergo oxidation by aldehyde dehydrogenases to form the inactive metabolites 4-ketocyclophosphamide and carboxyphosphamide, respectively. Aldophosphamide can undergo β-elimination to form active metabolites phosphoramide mustard and acrolein. This spontaneous conversion can be catalyzed by albumin and other proteins. Less than 5% of cyclophosphamide may be directly detoxified by side chain oxidation, leading to the formation of inactive metabolites 2-dechloroethylcyclophosphamide. At high doses, the fraction of parent compound cleared by 4-hydroxylation is reduced resulting in non-linear elimination of cyclophosphamide in patients. Cyclophosphamide appears to induce its own metabolism. Auto-induction results in an increase in the total clearance, increased formation of 4-hydroxyl metabolites and shortened t1/2 values following repeated administration at 12- to 24-hour interval.
**Product description**	**C**yclophosphamide for Injection, USP (lyophilized powder) is a sterile white cake containing cyclophosphamide and

mannitol and is supplied in vials for single dose use.

Solution preparation

Reconstitution of Cyclophosphamide: Reconstitute Cyclophosphamide using 0.9% Sodium Chloride Injection, USP or Sterile Water for Injection, USP with the volume of diluent listed below in Table 9.2.1. Add the diluent to the vial and gently swirl to dissolve the drug completely.

**Table 9.2.1. Reconstitution in preparation**		
**Strength**	**Volume of Diluent**	**Cyclophosphamide Concentration**
**500mg**	**25 mL**	**20mg per mL**
**1g**	**50 mL**	
**2g**	**100 mL**	

Dilution of Reconstituted Cyclophosphamide: Further dilute the reconstituted Cyclophosphamide solution to a minimum concentration of 2 mg per mL with any of the following diluents:

- 5% Dextrose Injection, USP
- 5% Dextrose and 0.9% Sodium Chloride Injection, USP 0.45% Sodium Chloride Injection, USP

To reduce the likelihood of adverse reactions that appear to be administration rate-dependent (e.g., facial swelling, headache, nasal congestion, scalp burning), cyclophosphamide should be injected or infused very slowly. Duration of the infusion also should be appropriate for the volume and type of carrier fluid to be infused.

Stability

Reconstituted lyophilized cyclophosphamide is chemically and physically stable for 24 hours at room temperature or for six days in the refrigerator; it does not contain any antimicrobial preservative and thus care must to ensure the sterility of prepared solutions.

Route of administration Intravenous

Drug procurement Cyclophosphamide must be obtained from commercial sources.

9.2.2 Fludarabine

Chemical Name: Fludarabine phosphate

Other Names: Fludara®

Classification: Purine analog, antimetabolite

Molecular Formula: $C_{10}H_{13}FN_5O_7P$

Mode of Action: Fludarabine phosphate is rapidly dephosphorylated to 2-fluoro-ara-A and then phosphorylated intracellularly by deoxycytidine kinase to the active triphosphate, 2-fluoro-ara-ATP. This metabolite appears to act by inhibiting DNA polymerase alpha, ribonucleotide reductase and DNA primase, thus inhibiting DNA synthesis. The mechanism of action of this antimetabolite is not completely characterized and may be multi-faceted.

Metabolism: Phase 1 studies in humans have demonstrated that fludarabine phosphate is rapidly converted to the active metabolite, 2-fluoro-ara-A, within minutes after IV infusion. Consequently, clinical pharmacology studies have focused on 2-fluoro-ara-A pharmacokinetics. After the five daily doses of 25 mg 2-fluoro-ara-AMP/m2 to cancer patients infused over 30 minutes, 2-fluoro-ara-A concentrations show a moderate accumulation. During a 5 day treatment schedule, 2-fluoro-ara-A plasma trough levels increased by a factor of about 2. The terminal half-life of 2-fluoro-ara-A was estimated as approximately 20 hours. In vitro, plasma protein binding of fludarabine ranged between 19% and 29%.

Product description: Fludara for injection is supplied as a white, lyophilized solid cake. Each vial contains 50 mg of fludarabine phosphate, 50 mg of mannitol and sodium hydroxide to adjust pH to 7.7. The pH range for the final product is 7.2-8.2.
Fludara for injection is supplied in a clear glass single dose vial (6 ml capacity).

Solution preparation: Fludarabine phosphate for injection should be prepared for parenteral use by aseptically adding sterile water for injection. When reconstituted with 2 ml of sterile water for injection, the solid cake should fully dissolve in 15 seconds or less; each ml of the resulting solution will contain 25 mg of fludarabine phosphate, 25 mg of mannitol, and sodium hydroxide to adjust the pH to 7.7. The pH range for the final product is 7.2-8.2. In clinical studies, the product has been diluted in 100 or 125 cc

	of 5% dextrose injection or 0.9% sodium chloride. Reconstituted fludarabine phosphate for injection contains no antimicrobial preservative and thus should be used within 8 hours of reconstitution. Care must be taken to assure the sterility of prepared solutions. Parenteral drug products should be inspected visually for particulate matter and discoloration prior to administration.
**Storage requirements*:***	Store under refrigeration, between 2-8°C (36-46°F).
**Stability:**	When stored as directed, the powder for injection is stable for at least 18 months after the date of manufacture. While early stability studies reported that the powder for injection was stable for at least 36 months when stored at 22–25°C, more recent studies employing assays with increased sensitivity have shown that the drug is less stable than this; therefore, the manufacturer currently recommends that fludarabine phosphate powder for injection not be stored at room temperature. Fludarabine phosphate is relatively stable in aqueous solutions, with optimal stability occurring at an approximately neutral pH. When reconstituted to a final concentration of 25 mg/mL, aqueous solutions of the drug are stable for at least 16 days at room temperature and normal light conditions. When diluted to a final concentration of 1 mg/mL, the drug is compatible in these diluents for at least 16 days at room temperature and normal light conditions. However, because such reconstituted and diluted fludarabine phosphate solutions contain no preservatives, the manufacturer recommends that they be used within 8 hours after preparation.
**Route of administration*:***	Intravenous
**Drug Procurement:**	Fludarabine must be obtained from commercial sources.

Fludarabine must be obtained from commercial sources and is available in 50 mg/6mL capacity vials. **The cost of this agent will be the subject's responsibility.**

10 EXPLORATORY or CORRELATIVE STUDIES

We will investigate the changes in cytokine serum concentrations, anti-CD19 CAR-T cell subpopulations and their correlation with adverse events, response and anti-CD19 CAR-T cell persistence (measured by flow cytometry and qPCR).

10.1 Methods

10.1.1 Cytokine plasma concentrations

Patient blood samples (5ml peripheral blood) will be collected according to the correlative sample calendar in Section 11.3. Samples will be processed in the Hematopoietic Biorepository and Cellular Therapy Core Facility of the Case Comprehensive Cancer Center. Samples will be held at -80°C for subsequent analysis using Meso Scale Discovery panels, with cytokines measured including Interleukin (IL)-1 Ra, IL-2 Rα, IL-6, IL-8, IL-10, IL-15, Granzyme A and B, Interferon gamma (IFNγ), chemokine (C-C motif) ligand 2 (CCL2), IFNγ – induced protein 10 (IP-10).

These analyses will be conducted by the Dr. David Wald's laboratory at the Case Comprehensive Cancer Center. MSD assays would be done using a Mesoscale (MSD) SECTOR® Imager 2400 multiplex analyzer.

Contact information for the processing of blood samples for cytokine serum concentrations:

David Wald, MD, PhD
Case Western Reserve University
Wolstein Research Building
2103 Cornell Road
WRB3-530
Cleveland, OH 44106
Telephone: 216-368-5668
Email: david.wald@uhhospitals.org

10.1.2 Flow cytometry - based phenotyping and persistence of anti-CD19 CAR-T cells

Each site will perform the flow cytometry - based phenotyping and persistence of anti-CD19 CAR-T cells on the cells processed and manufactured at their study site.

We will use flow cytometry to quantify the anti-CD19 CAR-T cell product prior to infusion as well as to characterize the changes of anti-CD19 CAR-T cell phenotype after infusion and *in vivo* expansion. Samples will include: A) anti-CD19 CAR-T cell product and B) peripheral blood samples from patients collected according to the correlative sample calendar in Section 11.3. In addition, flow cytometry will be used to measure the persistence of CART-T cells at the time points listed above.

We will perform flow cytometry testing of the anti-CD19 CAR-T cells using 6 panels of markers to assess the composition of the product, the transduction efficiency and viability, the differentiation status, proliferative capacity, immune cell sub population, exhaustion and activation status. The flow panels to be used for basic characterization are listed in table 10.1.2.

Dye	Cellular Composition and Viability	Transduction and Viability	Differentiation	Proliferative Capacity	Exhaustion	Activation
VioBlue	CD45	CD45	CD62L	CD27	CD223	CD154
VioGreen	CD4	CD4	CD4	CD4	CD4	CD4
FITC	CD3	CD3	CD3	CD3	CD3	CD3
PE	CD16/CD56	CAR*	CAR*	CAR*	CAR*	CAR*
PerCP	7-AAD	7-AAD	7-AAD	7-AAD	7-AAD	7-AAD
PE-Vio770	CD20		CD45RO	CD279	CD279	CD25**
APC	CD14	CD14	CD95	CD127	CD366	CD137
APC	CD8	CD8	CD8	CD8	CD8	CD8

Flow cytometry will be performed using a LSR2 (BD Biosciences) of the Flow Cytometry and Microscopy Core of the Case Comprehensive Cancer Center, and at Dr. Rafick Sekaly's laboratory for cells processed and manufactured at UHSCC-CTL. Washington University will perform this assay for cells processed and manufactured at their study site.

10.1.3 Anti-CD19 CAR-T Cell Persistance Assays

Each site will perform the Anti-CD19 CAR-T Cell Persistance Assays on the cells processed and manufactured at their study site.

Samples from patients collected according to the correlative sample calendar in Section 11.3.

For each patient sample collected, peripheral blood mononuclear cells (PBMCs) will be isolated using Ficoll centrifugation and cryopreserved. DNA will be extracted thawed samples using a Qiagen DNeasy blood and tissue kit. For detection of provirus, DNA from each timepoint will be assessed by qPCR using with a primers and probes specific for the CART vector backbone. The percentage of circulating T cells expressing the anti-CD19 CAR will be measured by flow cytometry using blood mononuclear cells stained with a fluorescein conjugated CD19 reagent. The percent CART detected will be reported as absolute numbers relative to blood lymphocytes and total mononuclear cells

These analyses will be conducted at the Hematopoietic Biorepository and Cellular Therapy Core Facility of the Case Comprehensive Cancer Center for cells processed and manufactured at UHSCC-CTL. Washington University will perform this assay for cells processed and manufactured at their study site.

10.1.4 Minimal residual disease

These analyses will be performed to determine the proportion of MRD negative patients as assessed by measurements of ctDNA with CAPP-Seq in patients treated with CAR-T cells. The overall hypothesis is that a large proportion of patients treated with this immune therapy achieve MRD negative status, which in turn correlates with improved progression free survival and long term survival of relapsed DLBCL patients. ctDNA measurements

will be done at the Laboratory of Prof. Carmelo Carlostella at Humanitas University in Milan, Italy.

Samples to be included in the analysis include those from patients collected according to the correlative sample calendar in Section 11.3.

Samples from participating sites will be shipped to Case Western Reserve University, and samples from across the entire study will be batched and shipped in temperature – regulated containers to the Laboratory of Professor Carmelo Carlo-Stella.

Carlo-Stella Laboratory.
Istituto Clinico Humanitas
Via Manzoni 56, Rozzano (MI), Italy.

For questions regarding the processing of blood samples for minimal residual disease or shipment to Case Western Reserve University, please contact:

Basabi Maitra
Case Western Reserve University
Telephone: 216 368-1495
Email: bxm@case.edu

11 STUDY PARAMETERS AND CALENDAR

11.1 Study Parameters

11.1.1 Screening Evaluation

Screening studies and evaluations will be used to determine the eligibility of each subject for study inclusion. All evaluations must be completed ≤ 28 days prior to patient enrollment on protocol.

Treatment Period

As this is a cellular therapy clinical trial, the treatment period will include the lymphodepletion regimen (starting on day -6), followed by the CAR-T cell infusion (day 0), with subsequent follow up, including the initial safety monitoring period (Day 1 to Day 30) and the survival observation period (day 30 and beyond). The trial observation period for dose limiting toxicities will conclude at day 30 (approximately 4 weeks after CAR-T cell infusion), while the trial follow-up will conclude 12 months after the CAR-T cell infusion. The study windows for day -6 through day 30 visits are ± 3 days, for laboratory tests and imaging studies. The study windows for visits from day 30 to 12 months/end of study are ± 7 days.

<u>Day - 6</u>

- Cyclophosphamide treatment (not on the reduced cyclophosphamide dosing cohort)

- Vital signs (body temperature, respiratory rate, blood pressure, heart rate, and weight)
- Physical examination
- ECOG Performance Status (PS)
- Assessment of adverse events
- Clinical laboratory assessments:
    - CBC with differential *(includes white blood cell count, hemoglobin, hematocrit, and platelets)*
    - CMP *(includes sodium, potassium, chloride, calcium, bicarbonate, blood urea nitrogen, creatinine, glucose, total bilirubin, alkaline phosphatase, ALT, AST, albumin, total protein)*
    - PT/PTT
    - magnesium
    - ferritin
- Serum pregnancy test for female patients of childbearing potential. Must be resulted prior to initiating treatment
- Correlative assay sample collection
- NOTE: In cases when CAR-T cell manufacturing requires extension for up to 96 hours, exact timing of lymphodepletive regimen may correspond up to 96 hours earlier.

Days -5 to -4

- Fludarabine treatment
- Cyclophosphamide treatment (ONLY ON THE REDUCED CYCLOPHOSPHAMIDE DOSING COHORT);
- Vital signs (body temperature, respiratory rate, blood pressure, heart rate, and weight)
- Physical examination
- ECOG PS
- Assessment of adverse events
- NOTE: In cases when CAR-T cell manufacturing requires extension for up to 96 hours, exact timing of lymphodepletive regimen may correspond up to 96 hours earlier.

Day- 3

- Fludarabine treatment
- Cyclophosphamide treatment (ONLY ON THE REDUCED CYCLOPHOSPHAMIDE DOSING COHORT);
- Vital signs (body temperature, respiratory rate, blood pressure, heart rate, and weight)
- Physical examination

- ECOG PS
- Assessment of adverse events
- Clinical laboratory assessment:
    - CBC with differential
    - CMP
    - PT/PTT
    - magnesium
    - ferritin
- NOTE: In cases when CAR-T cell manufacturing requires extension for up to 96 hours, exact timing of lymphodepletive regimen may correspond up to 96 hours earlier.

Day – 2
- Pre-infusion eligibility re-check (see section 4)

<u>Day 0</u>
- Vital signs (body temperature, respiratory rate, blood pressure, heart rate, and weight)
- ECOG PS
- Physical examination includine neurologic examination (to be performed prior to cell infusion)
- Assessment of adverse events
- Clinical laboratory assessment;
    - CBC with differential
    - CMP
    - PT/PTT
    - magnesium
    - ferritin
- CAR-T Cell infusion
- Correlative assay sample collection.

<u>Day +1 to day 7</u>
- Vital signs (body temperature, respiratory rate, blood pressure, heart rate, and weight)
- ECOG PS
- Physical examination including neurologic examination (day 6 only)
- CARTOX-10 assessment (see appendix 6)
- Assessment of adverse events
- Clinical laboratory assessment
- (to be done per protocol only on day 7; additional labs performed on other days as required per standard of care):
    - CBC with differential

  - CMP
  - PT/PTT
  - magnesium
  - ferritin
- Correlative assay sample collection (day 2 and day 6)
- Vector persistence on day 6.

<u>Day +14, Day +21, Day +30 (± 3 days)</u>

- Vital signs (body temperature, respiratory rate, blood pressure, heart rate, and weight)
- ECOG PS
- Physical examination including neurologic examination (days +14 and +21)
- Assessment of adverse events
- Clinical laboratory assessment:
  - CBC with differential
  - CMP
  - PT/PTT
  - magnesium
  - ferritin
- Response assessment (Day 30 only)
  - PET/CT for FDG avid lymphomas; CT scan (chest, abdomen and pelvis, neck if previously involved) for non FDG avid lymphomas.
  - Bone marrow biopsy based upon disease-specific studies described in Section 12.
- Vector persistence assay.
- Correlative assay sample collection

<u>Day + 60 (Follow up visits have a -/+ 7 day window)</u>

- Vital signs (body temperature, respiratory rate, blood pressure, heart rate, and weight)
- ECOG PS
- Physical examination
- Assessment of adverse events
- Clinical laboratory assessment
  - CBC with differential
  - CMP
  - PT/PTT
  - magnesium
  - ferritin
- Response assessment
  - PET/CT for FDG avid lymphomas; CT scan (chest, abdomen and pelvis, neck if previously involved) for non FDG avid lymphomas (may perform

    CT scan if subject already achieved remission and no suspicion of recurrence)
    - Bone marrow biopsy based upon disease-specific studies described in Section 12.
- Vector persistence assay.
- Correlative assay sample collection

Day +90 (Follow up visits have a -/+ 7 day window)

- Vital signs (body temperature, respiratory rate, blood pressure, heart rate, and weight
- Physical examination
- ECOG PS
- Assessment of adverse events
- Clinical laboratory assessment
    - CBC with differential
    - CMP
    - PT/PTT
    - magnesium
- Response assessment
    - PET/CT for FDG avid lymphomas if previous PET/CT scan was positive; CT scan (chest, abdomen and pelvis, neck if previously involved) if previous PET/CT scan was negative and for non FDG avid lymphomas (may perform CT scan if subject already achieved remission and no suspicion of recurrence).
- Vector persistence assay.
- RCL assay
- Correlative assay sample collection

Day +6 months, Day +12 months* (Follow up visits have a -/+ 7 day window)

- Assessment of disease status
- Record concomitant medications
- Vital signs (body temperature, respiratory rate, blood pressure, heart rate, and weight)
- ECOG PS
- Physical examination
- Clinical laboratory assessment;
    - CBC with differential
    - CMP
    - PT/PTT
    - magnesium
- Response assessment
- PET/CT for FDG avid lymphomas if previous PET/CT scan was positive; CT scan

(chest, abdomen and pelvis, neck if previously involved) if previous PET/CT scan was negative and for non FDG avid lymphomas (may perform CT scan if subject already achieved remission and no suspicion of recurrence).

- Vector persistence assay.
- RCL assay
- Correlative assay sample collection
- Long term follow up visit checklist (Appendix 4) (only for 12 month visit).

NOTE: ADDITIONAL SAMPLES FOR CORRELATIVE ASSAYS

- In the event a subject develops an immune mediated adverse event, additional samples for correlative assays (CAR-T cell detection, cytokines, T cell subpopulations) will be drawn. The samples will be drawn at the onset and follow up of the immune adverse event.

*Or End of Study Visit

11.2 Calendar

	Baseline	Day -8 or -9[1]	Day -6[2]	Day -5[2]	Day -4[2]	Day -3[2]	Day -1 or -2	Day 0	Day 1 - 7	Day 14	Day 21	Day 30	Day 60±7 days	Day 90±7 days	6 months	12 months	LTF[14] Years 2 - 5	LTF[14] Years 6 - 15
Autologous peripheral blood cell collection[1]		X																
Cyclophosphamide[2]			X[2]	X[2]	X[2]	X[2]												
Fludarabine[2]				X[2]	X[2]	X[2]												
CAR-T cell infusion								X										
Informed Consent	X																	
Inclusion/Exclusion	X																	
Pre-infusion Inclusion Criteria							X											
Medical History	X											X	X	X	X	X		
Disease status/Response assessment	X[3]											X[3]	X[3]	X[3]	X[3]	X[3]		
ECOG PS	X		X	X	X	X		X	X	X	X	X	X	X	X	X		
Vital Signs	X		X	X	X	X		X	X	X	X	X	X	X	X	X		
Physical Exam	X		X					X	X	X	X	X	X	X	X	X		
Neurologic examination								X[8]	X[9]	X	X							
CARTOX-10 assessment (Appendix 6)									X									
Concomitant Meds	X													X	X	X		
AE query	X		X	X	X	X	X	X	X	X	X	X	X	X	X	X		
LVEF measured with Echocardiogram or MUGA)	X																	
CBC w/diff	X		X			X		X	X[11]	X	X	X	X	X	X	X		
Serum Chemistries[4]	X		X			X		X	X[11]	X	X	X	X	X	X	X		
Ferritin	X		X			X		X	X[11]	X	X	X	X					
HIV	X																	
βHCG[5]	X[5]		**X**															
Bone Marrow Biopsy and Aspirate	X											X[3]	X[3]					
Imaging studies[6,7]	X											X	X	X	X	X		

	Baseline	Day -8 or -9[1]	Day -6[2]	Day -5[2]	Day -4[2]	Day -3[2]	Day -1 or -2	Day 0	Day 1 - 7	Day 14	Day 21	Day 30	Day 60±7 days	Day 90±7 days	6 months	12 months	LTF[14] Years 2 - 5	LTF[14] Years 6 - 15
CAR-T persistence assay[8]	X	X	X					X[8]	X[12]	X	X	X	X	X	X	X	X	
Long term follow up visit checklist[10]																X	X	
Long term follow up query checklist[10]																		X

Footnotes:

1. Autologous stem cell mobilization and collection to be done per institutional standards of University Hospitals Seidman Cancer Center. PLEASE NOTE ADDITIONAL REQUIREMENT to collect WBC and CD3 % the day PRIOR to collection. Autologous collection will occur on day -8 or -9 (± 96 hours) for fresh product infusions (variations in time will depend on available quantity of CAR-T cells after manufacturing period).
2. Cyclophosphamide is given on day -6 on standard dosing and on days -5, -4 and -3 on the reduced cyclophosphamide dosing cohort. If extension of CAR-T manufacturing is required to achieve planned dose, time points of conditioning regimen may vary in relation to day 0 up to 96 hours (i.e. extension of culture from 8 days to 12 days).
3. To be based on disease specific studies listed below and per section 12.
4. Albumin, alkaline phosphatase, total bilirubin, bicarbonate, BUN, calcium, chloride, creatinine, glucose, potassium, total protein, SGOT [AST], SGPT [ALT], sodium Calculated creatinine clearance will be done if creatinine and/or BUN are abnormal. **[[UH: Order COMP2],** Mg, , PT, and PTT.
5. Only for female participants of childbearing potential.
6. PET/CT scans for FDG avid lymphomas, CT scans for non-FDG avid lymphomas
7. PET/CT for FDG avid lymphomas if previous PET/CT scan was positive; CT scan (chest, abdomen and pelvis, neck if previously involved) if previous PET/CT scan was negative and for non FDG avid lymphomas.

To be done prior to CAR-T cell infTo be done on day 6

8. To be done on day 6
9. To be done on days 2, and **6**

10. Long term follow up. See Appendix 4 and 5 for LTF visit and query checklists, respectively.
11. Lab assessments to be done on day 7 only
12. if RCL not detected in first 12 months it can be discontinued

11.3 Correlation Sample Collection Calendar

Tubes	Short-Term Post-Infusion Follow-up								Long-Term	
	Baseline (prior to apheresis)	T-6	T0	T+2	T+6	T+14	T+21	T+30	T+60	T+9
EDTA *(10 ml, purple top)*	3 x 10 ml	2 x 10ml	3 x 10 ml	3 x 10 ml	3 x 10 ml	5 x 10 ml	2 x 10 ml	3 x 10 ml	3 x 10 ml	3 x 1
ACD *(8.5 ml, yellow top)*	2 x 8.5 ml	2 x 8.5ml	2 x 8.5 ml	2 x 8.5 ml	2 x 8.5 ml	2 x 8.5 ml	-	2 x 8.5 ml	2 x 8.5 ml	2 x 8.
PAXgene *(2.5 ml)*	1 x 2.5 ml	1 x 2.5ml	1 x 2.5 ml	1 x 2.5 ml	1 x 2.5 ml	1 x 2.5 ml	-	1 x 2.5 ml	1 x 2.5 ml	1 x 2.
**Total Volume**	**49.5 ml**	**39.5 ml**	**49.5 ml**	**49.5 ml**	**49.5 ml**	**69.5 ml**	**20 ml**	**49.5 ml**	**49.5 ml**	**49.5**

*** Twice yearly assays to be done on years 1 – 5. Subsequent assays to be done yearly on years 6 – 15 only on subjects with previously positive assays.**

12 MEASUREMENT OF EFFECT

The 2014 Lugano Response for Malignant Lymphoma will be used the following categories of response:

12.1 Lymphoma Response Criteria

NOTE: These criteria are based upon the criteria from the Revised Response Criteria for Malignant Lymphoma (22)

The criteria use the following categories of response: Complete Response (CR), Partial Response (PR), Stable Disease (SD), Relapse and Progression (PD). In the case of stable disease, follow-up assessments must have met the SD criteria at least once after entry to that step at a minimum interval of eight weeks.

The following guidelines are to be used for establishing tumor measurements at the baseline of each treatment step and for subsequent comparison:

- The six largest measurable nodes or extranodal masses must be identified as **Target Lesions** at baseline**.**
- If there are 6 or fewer measurable nodes and extranodal masses, all must be listed as **Target Lesions**
- If there are more than 6 involved measurable nodes or extranodal masses, the 6 largest nodes or extranodal masses should be selected as **Target Lesions** according to the following features: a) they should be clearly measurable in at least two perpendicular measurements; b) they should be from as disparate regions of the body as possible; and c) they should include mediastinal and retroperitoneal areas of disease whenever these sites are involved. When there are more than 6 involved measurable nodes or extranodal masses, any lesions

that are not included within these 6 Target Lesions will be considered non-measured lesions.

· **Nonmeasured lesions:** Any disease not selected as measured, dominant disease and truly assessable disease should be considered not measured. These sites include any nodes, nodal masses, and extranodal sites not selected as dominant or measurable or that do not meet the requirements for measurability but are still considered abnormal, as well as any site of suspected disease that would be difficult to follow quantitatively with measurement, including pleural effusions, ascites, bone lesions, leptomeningeal disease, abdominal masses, and other lesions that cannot be confirmed, measured or followed by imaging.

· Measurements for all Target Lesions will be reported at baseline of each treatment step. Measurements for non-measured lesions are not required.

· The lymph nodes or extranodal masses selected as **Target Lesions** for measurement should be measured in **two perpendicular diameters**, one of which is the longest perpendicular diameter. The lymph nodes should be measured in centimeters to the nearest one tenth of a centimeter (e.g. 2.0 cm, 2.1cm, 2.2 cm, etc.). **A measurable node must have a longest diameter (LDi) greater than 1.5 cm**. Measurable extranodal disease (eg, hepatic nodules) may be included in the six representative, measured lesions. A measurable extranodal lesion should have an LDi greater than 1.0 cm.

· The two measured diameters of each Target Lesion should be multiplied giving a product for each nodal site or extranodal mass. The product of each site should be added, yielding the sum of products of the diameters (SPD). The SPD will be used in determining the definition of response for those who have less than a complete response.

· PET-based response should use the following 5 point scale: PET 5 point scale: 1, no uptake above background; 2, uptake ≤ mediastinum; 3, uptake > mediastinum but ≤ liver; 4, uptake moderately > liver; 5, uptake markedly higher than liver and/or new lesions; X, new areas of uptake unlikely to be related to lymphoma.

12.1.1 Complete Response

Complete disappearance of all detectable clinical evidence of disease, and disease-related symptoms if present prior to therapy.

- PET-CT Based Criteria

  · Complete metabolic response with a 5 – point scale score of 1,2 or 3, with or without a residual mass.

  · In patients with bone marrow involvement before treatment there must be no residual FDG uptake in the marrow.

  · In patients with a typically FDG-avid lymphoma with no pre-treatment PET scan, or for lymphomas for which the PET scan was

positive prior to therapy: a post-treatment residual mass of any size is permitted as long as it is PET-negative.

- CT Based Criteria

  · For variably FDG-avid lymphomas without a pretreatment PET scan, or if a pretreatment PET scan was negative: all lymph nodes and extranodal masses must have regressed on CT to normal size (≤ 1.5 cm in their greatest transverse diameter for nodes > 1.5 cm prior to therapy). Previously involved nodes that were 1.1-1.5 cm in their long axis and > 1.0 cm in their short axis prior to treatment must have decreased to ≤ 1 cm in their short axis after treatment.

  · The spleen and/or liver, if considered enlarged prior to therapy on the basis of a physical examination or CT scan, should not be palpable on physical examination, and nodules related to lymphoma should disappear. However, no normal size can be specified because of the difficulties in accurately evaluating splenic and hepatic size and involvement. For instance, a spleen considered normal size may contain lymphoma, whereas an enlarged spleen may not necessarily reflect the presence of lymphoma, but variations in anatomy, blood volume, the use of hematopoietic growth factors, or other causes.

  · If the bone marrow was involved by lymphoma prior to treatment, the infiltrate must have cleared on repeat bone marrow biopsy. The biopsy sample on which this determination is made must be adequate (with a goal of

  > 20 mm unilateral core). If the sample is indeterminate by morphology, it should be negative by immunohistochemistry. A sample that is negative by immunohistochemistry but demonstrating a small population of clonal lymphocytes by flow cytometry will be considered a CR until data become available demonstrating a clear difference in patient outcome.

  § **NOTE:** Complete Remission/unconfirmed (CRu): Using the above definition for CR and that below for PR eliminates the category of CRu.

12.1.2 Partial Response (PR)

The designation of PR requires all of the following:

- PET-CT Based Criteria

  · Partial metabolic response with reduced uptake compared with baseline AND a 5 point scale score of 4 or 5.

  · For a typically FDG-avid lymphoma with no pretreatment PET scan or one that was PET-positive prior to therapy, the post-treatment PET should be positive at any previously involved sites.

  · In patients with bone marrow involvement before treatment, Residual uptake higher than uptake in normal marrow but reduced compared with

baseline (diffuse uptake compatible with reactive changes from chemotherapy allowed). If there are persistent focal changes in the marrow in the context of a nodal response, consideration should be given to further evaluation with MRI or biopsy or an interval scan.

- CT Based Criteria

    · For variably FDG-avid lymphomas/FDG-avidity unknown, without a pretreatment PET scan, or if a pretreatment PET scan was negative, CT scan criteria should be used.

    · A ≥ 50% decrease in sum of the product of the diameters (SPD) of up to 6 of the largest Target Lesions. These nodes or masses should be selected according to the following: (a) they should be clearly measurable in at least 2 perpendicular dimensions; if possible, they should be from disparate regions of the body; (b) they should include mediastinal and retroperitoneal areas of disease whenever these sites are involved.

    · No increase in the size of other nodes, liver or spleen.

    · Bone marrow assessment is irrelevant for determination of a PR if the sample was positive prior to treatment. However, if positive, the cell type should be specified, e.g., large-cell lymphoma or small cleaved cell lymphoma.

    · No new sites of disease.

    · Patients who achieve a CR by the above criteria, but who have persistent morphologic bone marrow involvement will be considered partial responders.

    · When the bone marrow was involved before therapy and a clinical CR was achieved, but with no bone marrow assessment after treatment, patients should be considered partial responders.

12.1.3 Stable Disease (SD)

- PET-CT Based Criteria:

    · Absence of metabolic response, with a score of 4 or 5 AND no significant change from baseline at interim or end of treatment.

    · In patients with bone marrow involvement before treatment, there must be no change from pre-treatment PET scan.

    · No new areas of FDG uptake.

- CT Based Criteria

    · For variably FDG-avid lymphomas/FDG-avidity unknown: For patients without a pretreatment PET scan or if the pre-treatment PET was negative, there must be no change in the size of the previous

lesions on the post-treatment CT scan.

· Less than 50% decrease from baseline in SPD of up to 6 Target Lesions.

· No increase in organ enlargement and non-measurable lesions compatible with progressive disease.

12.1.4 Progression (PD) and Relapse

- PET-CT Based Criteria

· Progressive metabolic disease:

· Individual target nodes and nodal masses must present increase intensity of uptake from baseline, with a 5 point score of 4 or 5, or

· Extranodal lesions with new FDG-avid foci consistent with lymphoma at interim or end of treatment assessment, or

· New FDG-avid foci consistent with lymphoma rather than another etiology (e.g. Infection, inflammation). If uncertain regarding the etiology of new lesions, a biopsy or repeat imaging scan should be considered.

- CT Based Criteria

· For determination of relapsed and progressive disease, lymph nodes should be considered abnormal if the long axis is more than 1.5 cm, regardless of the short axis. If a lymph node has a long axis of 1.1 to 1.5 cm, it should only be considered abnormal if the short axis is more than 1 cm. Lymph nodes ≤ 1 x ≤ 1 cm will not be considered as abnormal for relapse or progressive disease.

· At least a 50% increase from nadir in the SPD of any previously involved Target Lesions, or in a single involved node or extranodal mass, or the size of other lesions (e.g. splenic or hepatic nodules).

· To be considered progressive disease, a lymph node or extranodal mass with a diameter of the long or short axis axis of ≤ 2.0 cm must have increased by at least 0.5 cm; lesions larger than 2.0 cm must have increased by at least 1.0 cm.

· In the setting of splenomegaly, the splenic length must increase by >50% of the extent of its prior increase from baseline. If no prior splenomegaly, must increase by at least 2.0cm from baseline.

· New lesions: Regrowth of previously resolved lesions; or a new lymph node > 1.5cm in any axis; or a new extranodal site > 1.0 cm in any axis (new extranodal disease < 1.0 cm in any axis, can be considered progressive disease if its presence is unequivocal and attributable to lymphoma).

· New or recurrent bone marrow involvement

· Clinical Progressive Disease can be determined using the following criteria:

o ECOG PS of at least 3

o Patient unable to have follow-up radiologic Assessment due to performance status decline

o Symptomatic decline deemed related to metastatic disease or disseminated disease (not toxicity from therapy or concurrent illness)

12.2 Definitions of Time Periods

12.2.1 Duration of response

This is measured, only in responders, from the documented beginning of response (CR or PR) to the time of relapse.

12.2.2 Disease-free survival

Survival is defined as the date of study entry to the date of death. Disease-free survival is measured from the time of occurrence of disease-free state (e.g. the adjuvant setting following surgery or radiation therapy) or attainment of a complete remission) to disease recurrence or death from lymphoma or acute toxicity of treatment. This definition may be complicated by deaths that occur during the follow-up period that are unrelated to the lymphoma and there is controversy as to whether such deaths should be considered as events or censored at the time of occurrence. Whereas it is often possible to identify those deaths related to the lymphoma, there is the potential for bias in the attribution of deaths.

12.2.3 Disease-specific survival

Disease-specific survival (e.g., lymphoma-specific survival, cause-specific survival) is potentially subject to bias because the exact cause of death is not always easy to ascertain. To minimize the risk of bias, the event should be recorded as death from lymphoma, or from toxicity from the drug. Death from unknown causes should be attributed to the drug. For certain trials, time to next lymphoma treatment may be of interest, defined as time from the end of primary treatment until the initiation of the next therapy.

12.2.4 Progression-free survival

Progression-free Survival (PFS) is defined as the time from entry onto study until lymphoma progression or death from any cause. PFS reflects tumor growth and, therefore, occurs prior to the endpoint of overall survival. In addition, PFS is not confounded by the administration of subsequent therapy. Whether a prolongation of PFS represents direct clinical benefit or a surrogate for clinical benefit depends on the magnitude of the effect and the risk-benefit ratio of the therapy under investigation. Unlike survival, the precise date of progression is generally unknown. It may be defined as the first date of documentation of a new lesion or enlargement of a previous lesion, or the date of the scheduled clinic visit immediately after radiologic assessment has been completed. Where there is missing information, censoring of the data may be defined as the last date at which

progression status was adequately assessed or the first date of unscheduled new anti-lymphoma treatment.

12.2.5 Time to progression

Time to progression (TTP) is defined as the time from study entry until lymphoma progression or death due to lymphoma. In TTP, deaths from other causes are censored either at the time of death or at an earlier time of assessment, representing a random pattern of loss from the study. TTP is not as useful as PFS unless the majority of deaths on a study are unrelated to the lymphoma due to the efficacy of the treatment and/or prolonged follow up.

12.2.6 Time to treatment failure

Time to treatment failure (event-free survival) is measured from the time from study entry to any treatment failure including discontinuation of treatment for any reason, such as disease progression, toxicity, patient preference, initiation of new treatment without documented progression, or death. This composite endpoint is generally not encouraged by regulatory agencies because it combines efficacy, toxicity and patient withdrawal.

12.3 Response Review

Responses will be reviewed by the investigator (PI or co-investigator) who is treating the patient at each participating site.

13 DATA REPORTING / REGULATORY CONSIDERATIONS

Adverse event lists, guidelines, and instructions for AE reporting can be found in Section 8.0 (Adverse Events: List and Reporting Requirements).

13.1 Data Reporting

The OnCore™ and Forte EDC™ Databases will be utilized, as required by the Case Comprehensive Cancer Center, to provide data collection for both accrual entry and trial data management. OnCore™ is a Clinical Trials Management System housed on secure servers maintained at Case Western Reserve University. Forte EDC™ is electronic data capture system which supports 21 CRF Part 11 compliance. Access to data through Overture™ is restricted by user accounts and assigned roles. Once logged into the OnCore™ and Forte EDC™ systems with a user ID and password, defined roles for each user limit access to appropriate data. User information and password can be obtained by contacting the OnCore™ Administrator at OnCore-registration@case.edu.

OnCore™ and Forte EDC™ are designed with the capability for study setup, activation, tracking, reporting, data monitoring and review, and eligibility verification. This study will utilize electronic Case Report Form completion in the Forte EDC™ database. A calendar of events and required forms are available in OnCore™ and Forte EDC™

13.2 Regulatory Considerations

The study will be conducted in compliance with ICH guidelines and with all applicable

federal (including 21 CFR parts 56 & 50), state or local laws.

13.2.1 Written Informed consent

Provision of written informed consent must be obtained prior to any study-related procedures. The Principal Investigator will ensure that the subject is given full and adequate oral and written information about the nature, purpose, possible risks and benefits of the study as well as the subject's financial responsibility. Subjects must also be notified that they are free to discontinue from the study at any time. The subject should be given the opportunity to ask questions and be allowed time to consider the information provided.

The original, signed written Informed Consent Form must be kept with the Research Chart in conformance with the institution's standard operating procedures. A copy of the signed written Informed Consent Form must be given to the subject. Additionally, documentation of the consenting process should be located in the research chart.

13.2.2 Subject Data Protection

In accordance with the Health Information Portability and Accountability Act (HIPAA), a subject must sign an authorization to release medical information to the sponsor and/or allow the sponsor, a regulatory authority, or Institutional Review Board access to subject's medical information that includes all hospital records relevant to the study, including subjects' medical history.

13.2.3 Retention of records

The Principal Investigator of The Case Comprehensive Cancer Center supervises the retention of all documentation of adverse events, records of study drug receipt and dispensation, and all IRB correspondence for as long as needed to comply with local, national and international regulations. No records will be destroyed until the Principal Investigator confirms destruction is permitted.

13.2.4 Audits and inspections

Authorized representatives of the sponsor, a regulatory authority, an Independent Ethics Committee (IEC) or an Institutional Review Board (IRB) may visit the site to perform audits or inspections, including source data verification. The purpose of an audit or inspection is to systematically and independently examine all study-related activities and documents to determine whether these activities were conducted, and data were recorded, analyzed, and accurately reported according to the protocol, Good Clinical Practice (GCP), guidelines of the International Conference on Harmonization (ICH), and any applicable regulatory requirements. For multi-center studies, participating sites must inform the sponsor-investigator of pending audits.

13.2.5 Results Reporting

This study is registered with the National Institutes of Health National Library of Medicine's ClinicalTrials.gov and has been assigned the number NCT03434769. Study results will be released via ClinicalTrials.gov according to Section 801 of the Food and

Drug Administration Amendments Act of 2007 (FDAAA) and the Final Rule for Clinical Trials Registration and Results Information Submission (September 2016) within 1 year of the completion of the primary endpoint. Should the study be terminated early results will be reported via ClinicalTrials.gov within 1 year of study termination.

13.2.6 Relevance to Medicare Beneficiary Population

This study will investigate the feasibility of using CAR T-cells to treat relapsed/refractory non-Hodgkin lymphoma. The eligible study population includes subjects of Medicare beneficiary age. We anticipate that subjects of the Medicare beneficiary population will accrue at a similar rate to those of the non-Medicare beneficiary population. Results of this study can be generalized to the Medicare beneficiary population relapsed/refractory lymphoma.

14 STATISTICAL CONSIDERATIONS

With this type of phase I study design, the exact number of patients needed to complete the study is unknown, as it depends on the number of cohorts required to reach the MTD. A maximum of 18 patients can theoretically participate in the dose escalation, based on 3 dose levels, with a maximum of 6 patients at each dose level. Table 14.1 gives the probabilities of escalating the dose level under a true but unknown underlying rates of DLT. At a true DLT rate of 20%, the chance of escalating to the next dose level is 71% and of establishing the lower dose level as MTD is 29%. At a true DLT rate of 50%, the probability of escalating to the next dose level is 17%, and of establishing the prior dose level as the MTD is 83%.

Table 14.1 Probability of escalating dose levels

	True underlying DLT rate				
	0.1	0.2	0.3	0.4	0.5
Probability of escalating to next dose level	0.91	0.71	0.49	0.32	0.17
Probability of not escalating and establishing prior dose level as MTD	0.09	0.29	0.51	0.68	0.83

Expansion plan

In dose levels that have completed enrollment per dose escalation and which have not exceeded the maximum tolerated dose, enrollment may be expanded to further delineate toxicity. Each dose level that has not exceeded the maximum tolerated dose can be expanded by up to 12 patients.

If dose levels are expanded to further evaluate toxicity, up to 36 additional patients may be added if all 3 dose levels were expanded to the maximum.

14.1 Correlative studies

The results of correlative studies conducted in this trial will be analyzed with descriptive statistics, including evaluations of median, minimum and peak values. Area under the curve (AUC) will be used as a measure of cytokine secretion as well as of CAR-T cell expansion and persistence. Comparisons will be done between responding and non-responding patients using the Mann – Whitney test for quantitative variables and Fisher's exact test for categorical variables. The Wilcoxon test will be used to evaluate the changes in cytokine concentrations, CAR-T cell expansion and changes in T cell phenotype over time. While the sample size may not allow for achievement of statistical significance in association tests, we will evaluate the presence of associations between cytokine serum concentrations and CAR-T cell expansion (measured in absolute number changes, percentage changes and AUC) as well as with presence or absence or response and adverse events. Correlations between quantitative variables will be done with Spearman's correlation test, whereas correlation between categorical variables will be done using Fisher's exact test. Statistical analysis will be done using XLSTAT ®.

15 REFERENCES

(1) Howlader N, Mariotto AB, Besson C, et al. Cancer-specific mortality, cure fraction, and noncancer causes of death among diffuse large B-cell lymphoma patients in the immunochemotherapy era. Cancer 2017;123:3326-3334.

(2) Coiffier B, Lepage E, Briere J, et al. CHOP chemotherapy plus rituximab compared with CHOP alone in elderly patients with diffuse large-B-cell lymphoma. N Engl J Med 2002;346:235-242.

(3) Witzig TE, Vukov AM, Habermann TM, et al. Rituximab therapy for patients with newly diagnosed, advanced-stage, follicular grade I non-Hodgkin's lymphoma: a phase II trial in the North Central Cancer Treatment Group. J Clin Oncol 2005;23:1103-1108.

(4) Hiddemann W, Buske C, Dreyling M, et al. Treatment strategies in follicular lymphomas: current status and future perspectives. J Clin Oncol 2005;23:6394-6399.

(5) Rummel MJ, Niederle N, Maschmeyer G, et al. Bendamustine plus rituximab versus CHOP plus rituximab as first-line treatment for patients with indolent and mantle-cell lymphomas: an open-label, multicentre, randomised, phase 3 non-inferiority trial. Lancet 2013;381:1203-1210.

(6) Park JH, Riviere I, Wang X, Purdon T, Sadelain M Brentjens RJ. Impact of disease burden on long-term outcome of 19-28z CAR modified T cells in adult patients with relapsed B-ALL. JCO 2016;34:7003-7003.

(7) Lee DW, Kochenderfer JN, Stetler-Stevenson M, et al. T cells expressing CD19 chimeric antigen receptors for acute lymphoblastic leukaemia in children and young adults: a phase 1 dose-escalation trial. Lancet 2015;385:517-528.

(8) Maude SL, Teachey DT, Rheingold SR, et al. Sustained remissions with CD19-specific chimeric antigen receptor (CAR)-modified T cells in children with relapsed/refractory ALL. JCO 2016;34:3011-3011.

(9) Frey NV, Shaw PA, Hexner EO, et al. Optimizing chimeric antigen receptor (CAR) T cell therapy for adult patients with relapsed or refractory (r/r) acute lymphoblastic leukemia (ALL). JCO 2016;34:7002-7002.

(10) Turtle CJ, Hanafi LA, Berger C, et al. CD19 CAR-T cells of defined CD4+:CD8+ composition in adult B cell ALL patients. J Clin Invest 2016;126:2123-2138.

(11) Kochenderfer JN, Dudley ME, Kassim SH, et al. Chemotherapy-refractory diffuse large B-cell lymphoma and indolent B-cell malignancies can be effectively treated with

autologous T cells expressing an anti-CD19 chimeric antigen receptor. J Clin Oncol 2015;33:540-549.

(12) Kochenderfer J, Somerville R, Lu T, et al. Anti-CD19 chimeric antigen receptor T cells preceded by low-dose chemotherapy to induce remissions of advanced lymphoma. JCO 2016;34:LBA3010-LBA3010.

(13) Schuster SJ, Svoboda J, Dwivedy Nasta S, et al. Sustained Remissions Following Chimeric Antigen Receptor Modified T Cells Directed Against CD19 (CTL019) in Patients with Relapsed or Refractory CD19+ Lymphomas. Blood 2015;126:183-183.

(14) Turtle CJ, Hanafi LA, Berger C, et al. Immunotherapy of non-Hodgkin's lymphoma with a defined ratio of CD8+ and CD4+ CD19-specific chimeric antigen receptor-modified T cells. Sci Transl Med 2016;8:355ra116.

(15) Neelapu SS, Locke FL, Bartlett NL, et al. Axicabtagene Ciloleucel CAR T-Cell Therapy in Refractory Large B-Cell Lymphoma. N Engl J Med 2017.

(16) Schuster SJ, Svoboda J, Chong EA, et al. Chimeric Antigen Receptor T Cells in Refractory B-Cell Lymphomas. N Engl J Med 2017.

(17) Kochenderfer JN, Somerville RPT, Lu T, et al. Lymphoma Remissions Caused by Anti-CD19 Chimeric Antigen Receptor T Cells Are Associated With High Serum Interleukin-15 Levels. J Clin Oncol 2017;35:1803-1813.

(18) Lee DW, Gardner R, Porter DL, et al. Current concepts in the diagnosis and management of cytokine release syndrome. Blood 2014;124:188-195.

(19) Neelapu SS, Tummala S, Kebriaei P, et al. Chimeric antigen receptor T-cell therapy - assessment and management of toxicities. Nat Rev Clin Oncol 2018;15:47-62.

(20) Till BG, Jensen MC, Wang J, et al. Adoptive immunotherapy for indolent non-Hodgkin lymphoma and mantle cell lymphoma using genetically modified autologous CD20-specific T cells. Blood 2008;112:2261-2271.

(21) Till BG, Jensen MC, Wang J, et al. CD20-specific adoptive immunotherapy for lymphoma using a chimeric antigen receptor with both CD28 and 4-1BB domains: pilot clinical trial results. Blood 2012;119:3940-3950.

16 Appendices

16.1 APPENDIX 1. PERFORMANCE STATUS CRITERIA

**ECOG Performance Status Scale**		**Karnofsky Performance Scale**	
Grade	Description	Percent	Description
0	Normal activity. Full active, able to carry on all pre-disease performance without restriction.	100	Normal, no complaints, no evidence of disease.
		90	Able to carry on normal activity; minor signs or symptoms of disease.
1	Symptoms, but ambulatory. Restricted in physically strenuous activity, but ambulatory and able to carry out work of a light or sedentary nature (e.g., light housework, office work).	80	Normal activity with effort; some signs or symptoms of disease.
		70	Cares for self, unable to carry on normal activity or to do active work.
2	In bed < 50% of the time. Ambulatory and capable of all self-care, but unable to carry out any work activities. Up and about more than 50% if waking hours.	60	Requires occasional assistance, but is able to care for most of his/her needs.
		50	Requires considerable assistance and frequent medical care.
		30	Severely disabled, hospitalization indicated. Death not imminent.
4	100% bedridden. Completely disabled. Cannot carry on any self-care. Totally confined to bed or chair.	20	Very sick, hospitalization indicated. Death not imminent.
		10	Moribund, fatal processes progressing rapidly.
5	Dead.	0	Dead

16.2 APPENDIX 2. CELL PRODUCT RELEASE FORM

CASE 2417 WKS-002 CD19 CAR-T CHARACTERIZATION

**Patient Information**		
**Patient Study Number:**	**Secondary ID:**	**Date:**

**Release Testing**				
**The following Release Specifications for the cryopreserved product will be met prior to infusion.**				
**Test**	**Specification**	**Test Result**	**Pass/Fail**	**Performed By:**
Viability at harvest	≥ 70%			
CD19CAR+ cell dose	___________/kg per Cohort ___			
Endotoxin	≤ 5.00 EU/kg			
Detection of Microorganisms of Final Product	No bacteria or fungus present after 14 days			
Mycoplasma QPCR	Negative			
% CD4 and %CD8	Report results			
Replication Competent Lentivirus	Negative			

**QA Review**	
**Signature:**	**Date:**
**Comments:**	

16.3 APPENDIX 3. MANAGEMENT OF ADVERSE EVENTS FOLLOWING IMMUNE EFFECTOR CELL THERAPY (UHSCC SOP)

University Hospitals
Seidman Cancer Center

Stem Cell Transplant Clinical Program

SOP B7.41 MANAGEMENT OF PATIENTS RECEIVING IMMUNE EFFECTOR CELL THERAPY

PURPOSE: To describe the procedure for management of patients who have received immune effector cells (IECs) (including CAR T cells, virus-specific T cells, natural killer (NK) cells, regulatory T cells and dendritic cells). This SOP includes precautions and monitoring of adverse events including cytokine release syndrome (CRS).

OBJECTIVE: To provide a standardized and effective process for identifying and treating systemic adverse events resulting from severe inflammatory response to cytokines released by IECs. These cytokines include interleukin-6 and TNF-alpha.

SCOPE: SCT Physicians, SCT Inpatient and Ambulatory Nursing Staff, Code White Team, Medical ICU Staff (Physicians and Nurses), other ICU staff, Emergency Room Staff (Physicians and Nurses), Cellular Therapy Service, Transfusion Medicine, SCT Coordinators, Infusionists, Advanced Practice Professionals, Pharmacists, and all other staff who coordinate or manage the care for patients receiving IEC therapy. .

MATERIALS AND EQUIPMENT:

The pharmacies attached to the concerned units (SCC, MICU, ER) must have on formulary and in stock at all times: 0.9% Sodium Chloride, Intravenous Methylprednisolone, Intravenous Dexamethasone, Tocilizumab, Levetiracetam.

Transfusion medicine must readily have available blood products to include Cryoprecipitate, fresh frozen plasma (FFP).

PROCEDURE:

Routine care for ALL patients hospitalized following administration of IECs:

1. Verify the following:
    a. Patient has a patent double lumen central venous catheter at a minimum.
    b. Baseline Neuro exam and brain imaging are complete and available for review.
2. Verify the patient is receiving the following prophylactic therapies:
    a. Microbial prophylaxis with Bactrim and Acyclovir (or comparable agents). If patient is neutropenic (ANC<500) confirm patient is also receiving antibacterial prophylaxis with Ciprofloxacin (or comparable alternative).
    b. Allopurinol for tumor lysis prophylaxis
    c. Levetiracetam 500-750mg every 12 hours for seizure prophylaxis (till 30 days after cell infusion)
    d. Ursodiol 300mg every 8 hours.

3. ***AVOID*** Acetaminophen or NSAIDs.
4. ***Do Not*** administer any anti-hypertensive medications on the day of IEC infusion.
5. Obtain Vital Signs at following intervals:
    a. Baseline; prior to IEC infusion
    b. Upon completion of IEC infusion and then
    c. Every 15 minutes for 1 hour post completion of infusion until stable
    d. Every 4 hours until discharge ***(increase to every 2 hours if T>38°C (100.4°F) or HR>115bpm or oxygen dependent)***
    e. Consider telemetry monitoring for first seven days
6. Monitor ***strict*** Intake and Output every 6 hours
7. Obtain daily weights
8. Perform Neuro checks every 12 hours (CARTOX-10 exam to be completed at bedside); *see Figure 1 for summarized Neurological assessment definitions*
9. Obtain the following daily labs:
    a. CBC with differential
    b. Comprehensive metabolic panel
    c. Uric acid
    d. LDH
    e. PT
    f. PTT
    g. Fibrinogen
    h. Ferritin
    i. CRP
10. Minimum transfusion parameters for blood products
    a. Hb<8g/dL
    b. Platelets<20,000/uL
    c. Fibrinogen <150mg/dL (Cryoprecipitate)
11. If ANC is < 500/uL, initiate filgrastim until > 1500/uL
12. If IgG is < 400 mg/dL, provide monthly IVIG replacement and Long-term monitoring
13. Call APP/MD for the following:
    a. T>38°C (100.4°F)
    b. HR>115bpm, SBP<100mmHg (or <75% baseline)
    c. RR≥30, SaO_2<92%,
    d. Mental status changes.
14. Refer to Figures 2 to 5 for summarized CRS or Neurotoxicity Grading System (to be documented in patient's EMR) and management outline.

Care to be activated by SCC or Emergency Department staff in the event of suspected CRS or Neurotoxicity (by organ-system): ***summary in Figures 2 to 5***

Note: For fever, tachycardia, hypotension, tachypnea or hypoxia as outlined below, increase vital signs to every 2 hours until patient is back to normal parameters for at least 4 hours.

1. Fever (T>38°C (100.4°F)):
   a. Draw two sets of blood cultures (peripheral and central line access) AND order STAT broad-spectrum IV antibiotics (Zosyn, Meropenem or comparable)
   b. Obtain vital signs every 2 hours; consider telemetry monitoring.
   c. Obtain chest x-ray.
   d. Obtain Urinalysis with urine culture.
   e. If febrile and hypotensive, consider Vancomycin.
   f. Provide cooling blanket if T≥40°C (104°F).
   g. Avoid Meperidine (Demerol) for rigors (can lower seizure threshold).
   h. Consult Infectious Disease team.
2. Serum inflammatory markers: Notify MICU if CRP >20 (associated with onset of Grade >2 CRS)
3. Hypotension (SBP <90mmHg):
   a. Give 1L 0.9% Sodium Chloride bolus wide open.
   b. **If no change after 1L bolus, obtain ECG, draw serum troponin and notify Code White Team.**
   c. **If no change after second liter bolus, give Tocilizumab, obtain echocardiogram and request MICU transfer**
4. Tachycardia (HR>115bpm):
   a. Place patient on continuous cardiac monitoring (telemetry).
   b. Address fever or hypotension if also present as outlined above.
   c. **If sustained tachycardia over 2 hours, draw serum troponin, obtain ECG, and notify Code White Team.**
5. Tachypnea (RR>30) or Hypoxia (SaO_2 <92% on room air):
   a. Order STAT portable chest x-ray.
   b. Administer bronchodilator nebulizer treatment (Ipratropium / Atrovent).
   c. Start oxygen by nasal cannula at 2Lpm, **notify Code White Team.**
   d. **If requiring >4Lpm or FiO_2>40% to keep SaO_2>92%, give Tocilizumab and transfer to MICU.**
6. Disseminated intravascular coagulopathy / DIC (PT or PTT>1.5 times the upper limit of normal; Fibrinogen <150):
   a. Order Cryoprecipitate.
   b. Transfuse platelets if count is ≤20,000.

   c. Consider FFP if PTT>1.5 times upper limit of normal.
   d. **If PT/PTT is >2.5 X upper limit of normal OR Fibrinogen <75mg/dL, give Tocilizumab**
7. Neurotoxicity:
   a. Notify APP/MD in the event of headache, mental status changes, confusion, delirium, word finding difficulty, difficulty reproducing written name / signature, frank aphasia, hallucinations, new tremor or incoordination, altered gait.
   b. Obtain STAT brain imaging and Neurology Consult, Consider LP, EEG.
   c. **For severe headache lasting >24hours and resulting in new neurologic symptoms or any other neurotoxicity symptoms of Grade 3 (see Table 2 below), start IV Dexamethasone 10mg every 6 hours for 8 doses OR IV Methylprednisolone 1mg/kg every 12hours until symptoms have improved to ≤Grade 1.**
   d. **If Grade 4 Neurotoxicity (obtunded, seizures, cerebral edema, papilledema or new motor weakness) start IV Methylprednisolone 1g/day for 3 days then taper steroids slowly**
   e. **If seizures, start load with Lorazepam and Levetiracetam 1000mg.**

SPECIAL CIRCUMSTANCES (GRADE 3 or HIGHER CRS / NEUROTOXICITY REQUIRING MICU CARE)

1. **Respiratory Toxicity:**
   a. Oxygen requirement ≥40% FiO_2 or ≥4Lpm or requiring mechanical ventilation.
   b. Management per MICU guidelines.
2. **Cardiovascular Toxicity:**
   a. If requiring >1L normal saline bolus to maintain SBP>90mmHg or >75% baseline, or wide pulse pressure, track serial troponins, ECG, obtain echocardiogram.
   b. Give Tocilizumab if SBP remains <90mmHg after second fluid bolus
   c. Start low dose vasopressor (Norepinephrine is preferred as first-line) if requiring >2L normal saline to maintain MAP>60 or unable to tolerate bolus intravenous fluids due to volume overload or depressed LVEF.
3. **Indications for Tocilizumab Administration:**
   a. Hypotension not responsive to 2L normal saline bolus; LVEF<40%.
   b. FiO_2 requirement >40% for >2h; Dyspnea requiring mechanical ventilation.
   c. PT/aPTT>2.5xULN; Fibrinogen <150mg/dL on more than 2 consecutive days or less than 75mg/dL; clinically significant bleeding due to DIC.
   d. Creatinine >3 times higher than pre-infusion baseline or >3xULN
   e. Transaminitis >20 ULN or Total bilirubin >10xULN

Fig 2. Acute Toxicity Management: Grade 1

Organ / System	Definition	Management
Fever	Temp >38C / 100.4F	•Draw two sets of blood cultures<br>•STAT broad-spectrum IV antibiotics (+Vanco if also hypotensive)<br>•Vital signs q2h<br>•Chest x-ray.<br>•UA + culture.<br>•Acetaminophen; Cooling blanket if T≥40°C (104°F; start maintenance IV fluids<br>•Avoid meperidine (Demerol) for rigors (can lower seizure threshold).<br>•Consult Infectious Disease.
Systemic	N/V, diarrhea, myalgia	Symptomatic management
Neuro	Headache<br>CARTOX-10 score 7-9	Analgesics; aspiration precaution; low dose lorazepam/haloperidol for agitation; Fundoscopy; Neuro consult; CT/MRI brain; Consider LP and EEG; Toci if also has CRS Grade 2
Consider Tocilizumab if fever >72 hours and no infection		

Fig 3. Acute Toxicity Management: Grade 2

Organ / System	Definition	Management
Fever	As in Grade 1	As in Grade 1
Cardiac	BP<90mmHg; HR>120x2h	NS bolus up to 2L; Tocilizumab if no response after 2L; start low dose vasopressor (norepi preferred initial)<br>Obtain ECG, Troponins
Respiratory	SaO2<92% on room air, O2 needs <40% FiO2	Obtain CXR; supplemental O2; support; consider Tocilizumab if sustained >24h
Coagulation	PT/PTT 1.5-2.5xULN; Fibrinogen 75-149mg/dL	As needed Cryoprecipitate, FFP
Neuro	CARTOX-10 score 3-6	As for Grade 1 *and* ICU transfer if + CRS Grade 2; Dex 10mg q6h or Methylpred 1mg/kg q12h
Renal	Cr 1.5-3.0xbase; 1.5-3.0xULN	IV fluids; Renal consult
Liver	ALT/AST 3-5xULN, ALP2.5-5xULN, Bili 1.5-3xULN	Symptomatic, liver ultrasound, Ursodiol

Fig 4. Acute Toxicity Management: Grade 3

Organ / System	Definition	Management
Fever	As in Grade 1	As in Grade 1
Cardiac	As in Grade 2 ***AND*** needs high dose of 1 or multiple vasopressors	As in Grade 2 + Dex 10mg q6h
Respiratory	Oxygen needs ≥40% FiO2	Tocilizumab; NIPPV; + Dex 10mg q6h
Coagulation	PT/PTT >2.5xULN; Fibrinogen 30-74mg/dL; abn + hemorrhage	As in Grade 2 + Tocilizumab
Neuro	CARTOX-10 score 0-2; CSF opening pressure <20; papilloedema Stage 1-2; partial seizures	ICU transfer<br>Steroids as in Grade 2, continue till Grade 1 before taper;<br>Lorazepam + Levetiracetam for seizures
Renal	Cr >3.0xbase; 3-6xULN	As in Grade 2 + Tocilizumab
Liver	ALT/AST/ALP 5-20xULN, T.bili>10xULN	As in Grade 2 + Tocilizumab
**ICU transfer required for Grade 3 and Grade 4**		

Fig 5. Acute Toxicity Management: Grade 4

Organ / System	Definition	Management
Fever	As in Grade 1	As in Grade 1
Cardiac	As in Grade 3, life-threatening	As in Grade 3 + Methylpred 1g/day
Respiratory	Needs mechanical ventilation	As in Grade 3 + Methylpred 1g/day
Coagulation	>Grade 3	As in Grade 2 + Tocilizumab
Neuro	Critically obtunded; cerebral edema; CSF opening pressure ≥ 20; papilloedema Stage 3-5; generalized seizures; new motor weakness	Mech vent for airway protection; Dex 10mg/kg q6h x 8 doses or Methylpred 1g/day x 3; taper after improvement to Grade 1
Renal	>Grade 3	As in Grade 3 + Tocilizumab + Dex 10mg q6h
Liver	>Grade 3	As in Grade 3 + Tocilizumab + Dex 10mg q6h
**ICU transfer required for Grade 3 and Grade 4**		

References:

1. Bonifant CL, Jackson HJ, Brentjens RJ, Curran KJ. Toxicity and management in CAR T-cell therapy. Mol Ther Oncolytics. 2016 Apr 20;3:16011. doi: 10.1038/mto.2016.11. eCollection 2016.
2. Brudno JN, Kochenderfer JN. Toxicities of chimeric antigen receptor T cells: recognition and management. Blood. 2016; 127(26):3321-3330
3. Casucci M, Hawkins RE, Dotti G, Bondanza A. Overcoming the toxicity hurdles of genetically targeted T cells. Cancer Immunol Immunother (2015) 64:123–130
4. Davila ML, Riviere I, Wang X et al. Efficacy and Toxicity Management of 19-28z CAR T Cell Therapy in B Cell Acute Lymphoblastic Leukemia. Sci Transl Med 6, 224ra25224ra25.
5. Lee DW, Gardner R, Porter DL, Louis CU, Ahmed N, Jensen M et al. Current concepts in the diagnosis and management of cytokine release syndrome. Blood. 2014;124(2):188-195)
6. Neelapu SS, Tummala S, Kebriaei P et al. Chimeric antigen receptor T-cell therapy — assessment and management of toxicities. Nat Rev Clin Oncol. 2017 Sep 19. doi: 10.1038/nrclinonc.2017.148.
7. https://evs.nci.nih.gov/ftp1/CTCAE/CTCAE_4.03_2010-06-14_QuickReference_5x7.pdf
8. http://factwebsite.org/immuneeffectorcells/

Change History:

N/A – New

16.4 APPENDIX 4: LONG TERM FOLLOW UP CHECKLIST. YEARS 1 – 5 AFTER TREATMENT.

CHECKLIST FOR CLINICAL VISIT FOR LONG TERM FOLLOW UP OF CLINICAL TRIAL CASE 2417. YEARS 1 – 5 AFTER CAR-T CELL THERAPY FOR B CELL MALIGNANCIES	
DOCUMENT DATE AND TIME	▪ Document date and time<br>▪ Document time since Anti-CD19 CAR-T cell therapy
HISTORY	▪ Perform clinical history<br>▪ Document new conditions<br>▪ New malignancy(ies)<br>▪ New incidence or exacerbation of a pre-existing neurologic disorder<br>▪ New incidence or exacerbation of a pre-existing rheumatologic or autoimmune disorder<br>▪ New incidence of a hematologic disorder.
EXPOSURE HISTORY PHYSICAL EXAMINATION	▪ Exposure to mutagenic agents<br>▪ Exposure to new medications<br>▪ Perform physical examination
LABORATORY RESULTS	▪ CBC and differential<br>▪ Vector persistence assay<br>▪ RCL assay
UPDATE STUDY CONTACT INFORMATION	▪ Give subject the latest contact information for the clinical trial coordinator and principal investigator.

16.5 APPENDIX 5. LONG TERM FOLLOW UP CHECKLIST. YEARS 6 - 15 AFTER TREATMENT.

CHECKLIST FOR QUERY FOR LONG TERM FOLLOW UP OF CLINICAL TRIAL CASE 2417. YEARS 6 - 15 AFTER CAR-T CELL THERAPY FOR B CELL MALIGNANCIES	
Subjects may be contacted during an in office clinical visit, via telephone or written questionnaire. If vector persistence testing needed, this will be obtained at the time of an office visit.	
DOCUMENT DATE AND TIME	▪ Document date and time<br>▪ Document time since Anti-CD19 CAR-T cell therapy
HISTORY	▪ Perform clinical history<br>▪ Document new conditions<br>▪ New malignancy(ies)<br>▪ New incidence or exacerbation of a pre-existing neurologic disorder<br>▪ New incidence or exacerbation of a pre-existing rheumatologic or autoimmune disorder<br>▪ New incidence of a hematologic disorder.
LABORATORY RESULTS	▪ Vector persistence assay (if previously positive)<br>▪ RCL assay (if previously positive)
UPDATE STUDY CONTACT INFORMATION	▪ Give subject the latest contact information for the clinical trial coordinator and principal investigator.

16.6 APPENDIX 6. CARTOX-10 NEUROLOGIC ASSESSMENT OF CAR-T – ASSOCIATED ENCEPHALOPATHY (19)

**CARTOX-10**	**Points (normal = 10)**
**Orientation**<br>Year<br>Month<br>City<br>Hospital<br>President	 **1**<br>**1**<br>**1**<br>**1**<br>**1**
**Write name and a standard sentence**	**1**
**Name 3 objects**	**3**
**Count back from 100 in tens**	**1**

Supplementary Note 2: ALL study protocol

NMRC PHOI MB.CAR-T.CD19-ALL

Clinical Study Protocol

Safety and efficiency of anti-CD19 chimeric antigen receptor (CAR)- transduced T-cell therapy for pediatric and young adult patients with relapsed/refractory B-cell acute lymphoblastic leukemia: a single centre, non-randomised, open label phase I-II clinical trial of automatically produced cell therapy product MB CART 19.1 using CliniMACS Prodigy

Principal Investigator
Maschan M. A.

Study Coordinator
Molostova O. O.

Study Group

Maschan M. A.
Karachunsky A. I.
Myakova N. V.
Shelikhova L. N. (Deputy Study Manager, clinic)
Kurnikova E. E.
Muzalevsky Y. O. (Production Team Manager)
Pershin D. E. (Cell Preparation Quality Control)
Popov A. M.
Khamin I. G.
Shekhovtsova Z. B.
Novichkova G. A.
Dyakonova Y. G.

Table of Contents

Abbreviations……………………………………………………………………………
Summary…………………………………………………………………………………..
Introduction…………………………………………………………………………….
Justification of the approach……………………………………………………………
Principle of the method………………………………………………………………
Results of preclinical studies…………………………………………………………
Results of clinical studies……………………………………………………………
Unresolved issues……………………………………………………………………
Purposes and objectives of the study……………………………………………………
Purpose of the study…………………………………………………………………….
Objectives of the study………………………………………………………………..
Criteria for patients' inclusion in the study………………………………………………
Characteristics of the medical cell product………………………………………………
Chimeric receptor structure ……………………………………………………………..
Method of sampling and selection of initial cell population ………………………………
Transduction method…………………………………………………………………….
Expansion method……………………………………………………………………
Final characteristics of autologous cell product……………………………………………
Treatment plan…………………………………………………………………………..
Lymphodepleting
chemotherapy…………………………………………………………………………
Autologous cell product…………………………………………………………………
Dose modification during the study…………………………………………………….
Prevention and treatment of complications…………………………………………………
Clinical and laboratory monitoring plan…………………………………………………..
Endpoints…………………………………………………………………………………
Primary endpoints……………………………………………………………………
Secondary endpoints…………………………………………………………………..
Statistical plan of the study……………………………………………………………
Planned sample size……………………………………………………………………..
Safety precautions for study participants………………………………………………..
Use of data obtained during the study…………………………………………………..
Information for the study participants…………………………………………………

Informed consent ……………………………………………………………………………
List of reference…………………………………………………………………………………

Abbreviations

HSCT – hematopoetic stem cell transplantation

ALL – acute lymphoblastic leukaemia

CAR-T – lymphocytes expressing the chimeric antigenic receptor

Synopsis

Sponsor:	Dmitriy Rogachev National medical research center of pediatric hematology, oncology and immunology<br>Samory Mashela street, 1 117997, Moscow
Conditions:	B-lineage acute lymphoblastic leukemia
Purpose:	Evaluation of the safety and efficiency of autologous CD19 CAR-T lymphocytes in a cohort of pediatric and young adult patients with relapsed /refractory B—lineage acute lymphoblastic leukemia
Phase:	Phase I-II
Study type:	Interventional<br>Intervention:<br>- Leukepheresis<br><br>- Drug therapy:<br>Fludarabine<br>Cyclophosphamide<br><br>- Biological:<br>autologous CD19 CAR-T lymphocytes<br><br>Dose finding<br><br>Dose escalation<br>• Level 1 $5x10^5$/kg CD19 CAR-T lymphocytes<br>• Level 2 $1x10^6$/kg CD19 CAR-T lymphocytes<br>• Level 1 $3x10^6$/kg CD19 CAR-T lymphocytes<br>• Level 0 $1x10^5$/kg CD19 CAR-T lymphocytes<br>(in case of dose-limiting toxicity at dose level 1)
Study design:	Endpoint : safety/efficacy study<br>Intervention model: Single Group Assignment<br>Masking: open label<br>Primary Purpose: Treatment
Official Title:	Safety and efficiency of anti-CD19 chimeric antigen receptor (CAR)-transduced T-cell therapy for pediatric and young adult patients with relapsed/refractory B-cell acute lymphoblastic leukemia: a single centre, non-randomised, open label phase I-II clinical trial of automatically produced cell therapy product MB CART 19.1

Primary Outcome Measures:	<u>Safety:</u><br>Toxicity evaluation following CD19 CAR T-cell infusion (time frame 1 month):<br>- incidence of grade 3-5 SAE occurring within 30 days of CD19CAR T-cell infusion<br>- incidence of grade 3-4 Severe Cytokine Release Syndrome<br>- incidence of grade 3-5 neurotoxicity occurring within 30 days of CD19 CAR T-cell infusion<br><br><u>Efficacy:</u><br>- Probability of MRD-negative remission in all patients (time frame: 1 month)<br>- Probability of hematologic remission in all patients (time frame: 1 month)<br>- The proportion of patients achieving molecular remission at 1 month
Secondary outcome measures	• Duration of MRD-negative remission (time frame 2 years)<br>• Persistence/frequency of CD19 CAR T lymphocytes in peripheral (FC+qPCR) (time frame 2 years)<br>• Duration of B-cell aplasia ang hypogammaglobulinemia (time frame 5 years)<br>• Overall survival (time frame 5 years)<br>• Safety and adverse effects long-term documentation (time frame 5 years)
Planned enrollement	18 patients
Study start:	Q12018
Stady finish:	Q12023
Eligibility:	Diagnosis: CD19+B-cell leukemia that has not responded to standard treatment<br>Age: between 3 months and 25 years of age<br>Genders: both<br>Healthy volunteers: no
Inclusion criteria:	0) Ability to give informed consent (for patients > 14 years old). For subjects < 18 years old their legal guardian must give informed consent<br><br>1) Patients with relapsed or refractory CD19-expressing B cell ALL :<br>• Induction failure, no CR after course 2 or MRD>0,1% after 3 courses of high-risk protocol

	• early bone marrow or combined relapse of acute lymphoblastic leukaemia, no CR or MRD>0,1% after 1 course 2-nd line therapy<br>• ALL post ≥ 2nd relapse, no CR or MRD>0,1% after 1 course 2-nd line therapy<br>• Relapse or MRD >0,1% of ALL after stem cell transplant (> 60 days post alloHSCT)<br>• Late bone marrow or combined relapse of acute lymphoblastic leukaemia, no CR or MRD>0,1% after 2nd course of 2-nd line therapy<br>2) There must be no available alternative curative therapies<br>3) CD19 expression must be detected on greater than 30% by flow cytometry<br><br>4) Patients must have measurable or evaluable disease at the time of enrollment,<br>which may include any evidence of disease including minimal residual disease<br>detected by flow cytometry, cytogenetics, or polymerase chain reaction (PCR)<br>analysis.<br><br>5) Patient Clinical Performance Status: Karnofsky >50% or Lansky >50%<br><br>6) Patient Life Expectancy > 8 weeks<br><br>8) Patients recovered from acute toxic effects of all prior chemotherapy, immuno-<br>or radiotherapy<br><br>9) Patient absolute lymphocyte N > or =100/mm3<br><br>11) Patient cardiac function: left ventricular ejection fraction greater than or equal to 40% by MUGA or cardiac MRI, or fractional shortening greater than or equal to 28% by ECHO or left ventricular ejection fraction greater than or equal to 50% by ECHO.<br><br>12) Patients who agree to long-term follow up for up to 5 years (if received CD19 CAR-T cell infusion)
Exclusion criteria:	1. <30% expression of CD19 on the leukemic population<br>2. Active hepatitis B, C or HIV infection<br>3. Oxygen saturation < or = 90%<br>4. Bilirubin >3x upper norma limit

	5. Creatinine >3x upper norma limit<br>6. Active acute GVHD overall grade ≥2 (Seattle criteria)<br>7. Moderate/severe chronic GVHD (NIH consensus) requiring systemic steroids<br>8. Clinical signs of grade >3 CNS disorders (seizure disorder, paresis, aphasia, cerebrovascular, ischemia/hemorrhage, severe brain injuries, dementia, cerebellar disease, organic brain syndrome, psychosis, coordination or movement disorder)<br>9. Pregnant or lactating women.<br>10. Active severe infection
Arms	One Arm - experimental CD19 CAR T-cells
Study plan	<u>Key stages</u><br><br>1. Registration and informed consent sign<br>2. Clinical and laboratory evaluation, eligibility check<br>3. Unstimulated leukapheresis<br>4. Lymphodepleting chemotherapy: cyclophosphamide total dose 750 mg/m^2 and fludarabine total dose 120 mg/m^2<br>5. Infusion of the CD19 CAR-T lymphocytes<br>6. Monitoring and clinical care inpatient (till recovery of blood counts)<br>7. Monitoring and clinical care outpatient (according to schedule)<br>8. Replacement therapy by IVIG in patients with persistent hypogammaglobulinemia
Further therapy	Choice of further therapy beyond primary endpoint evaluation date, including HSCT indication, will be determined by the protocol study group according to the best interest of the patient
Follow up	Subjects will be followed every 6 months for 5 years following the 1st infusion<br>of CD19 CAR- T cells.

Introduction

Modern programmed chemotherapy for acute lymphoblastic leukaemia (ALL) ensures recovery of 70-80% of patients in the paediatric population and up to 50% of patients among adults. Allogenous hematopoietic stem cell transplantation (HSCT) is necessary to cure high-risk patients and recurrent disease. Despite the improvement of chemotherapy and transplantation methods, up to 20% of children and up to 50% of adult patients with ALL die because of disease progression. Patients cured of ALL often face a number of severe complications resulting in chronic health issues and disability. In view of the aforesaid, there is urgent need to develop innovative therapy methods ALL, which would exceed the existing standard methods in terms of efficacy and safety indicators.

One of the most promising new ALL therapy methods is immunotherapy based on derivatives of monoclonal antibodies aimed at surface antigens, selectively expressed on the surface of tumour cells. The most significant therapeutic efficacy was shown by autologous T-lymphocytes carrying the chimeric antigen receptor (CAR) to the tissue-specific antigen CD19 expressed on the surface of tumour cells with B-linear ALL and normal B lymphocytes. Expression of chimeric antigenic receptor for CD19 is provided by stable transduction of patient's T-lymphocytes ex-vivo. This type of cellular immunotherapy was named CAR-T therapy (deriving from chimeric antigen receptor T cells). The results of several published clinical studies confirm the unprecedented efficacy of CD19 CAR-T lymphocytes in treatment of recurrent and refractory ALL in children and adults, including the incidence of molecular remissions of 70-90% and event-free survival for 1 year to 50%. The technology of ALL CD19 CAR-T lymphocyte therapy is in the early stage of development; several technologies of autologous CD19 CAR-T lymphocyte production are undergoing registration in the USA and the EU for use in case of indications of recurrent/refractory B-linear ALL in children and young adults and recurrent/refractory B-cell non-Hodgkin's lymphomas.

The purpose of this protocol is to carry out the study of phases 1-2 of efficacy and safety for autologous CD19 CAR-T lymphocytes in recurrent/refractory ALL in children and young adults. The principal technological innovation is the semi-automatic system for production of an individual autologous cellular product using bioreactor CliniMACS Prodigy.

Justification of the approach

Principle of the method

The principle of use of chimeric antigenic receptors in biology and medicine was formulated in the late 1980s by Z. Eschar. The method is based on combining an antigen-recognising fragment borrowed from a monoclonal antibody of required specificity and signal sequence borrowed from the CD3 complex – physiological signal component of T-cell receptor into one molecule. Expression of such chimeric antigenic receptor in T-lymphocytes results in creation of effector population with predetermined specificity. Improvement of technology using single-chain, variable fragments of antibodies (scFv from a single-chain variable fragment) facilitated the creation of chimeric receptors of different specificity. A fundamental breakthrough that opened the way to clinical application of CAR-T lymphocytes occurred in the early 2000s, when second generation CARs appeared; a signal fragment of CD28 molecule – physiological co-gene ensuring the second signal required for full activation, proliferation of T-lymphocyte and maturation of its effector functions – was sequentially inserted into the intracellular part of the receptor (Figure 1). It has now been proven that the inclusion of signal sequences derived from various natural co-stimulatory receptors in the chimeric antigen receptor significantly influences the important functional characteristics of CAR-T lymphocytes, such as proliferation, persistence, depletion, and others. Additional necessary requirements for clinical use of CAR-T lymphocytes were the choice of the target antigen and the development of an effective transduction mechanism. The key characteristics of the antigen are stable expression on the surface of all (vast majority) tumour cells and lack of expression on healthy (critically important) tissues. CD19 antigen mostly meets these requirements, as the function of B-lymphocytes normally expressing this antigen can be safely replaced for a long time by donor intravenous immunoglobulin infusions. The retroviral vector was used as transduction method of chimeric antigenic receptor in earlier works; lentiviral vectors and alternative delivery systems based on mobile genetic elements were used in more recent works. The key requirement for transduction system is high efficacy, stable transgene expression and safety from the point of view of insertional oncogenesis. It should be noted that, unlike transduction of hematopoietic stem cells, the genetic modification of mature T lymphocytes, irrespective of the vector used, did not result in oncogenesis in numerous preclinical and clinical studies. A lentiviral vector of the third generation will be used in this protocol to produce an autologous medical cellular product. The most developed scheme of CD19 CAR-T lymphocyte application attached to the clinical application includes the following key stages: 1) collection of leukocyte fraction in a patient with ALL; 2) ex-vivo transduction of the patient's T lymphocytes with introduction of the transgene encoding an antigenic receptor specific of the second generation

for CD19 antigen; 3) a course of lymphoblastic chemotherapy, and 4) autologous CD19 infusion for CAR-T patient (Figure 2).

Results of preclinical studies for similar drugs

The main CD19 CAR constructions were studied in various preclinical models, including NSG mouse models. Numerous studies showed high efficiency of constructions of the second generation in terms of xenografts of human B-linear tumours. Significant toxic effects in preclinical models were not foreseen.

Results of clinical studies for similar drugs

The first data from clinical studies of CD19 CAR T lymphocytes in B-linear tumours were published in 2011. The first reports already demonstrated the key therapeutic characteristics of CD19 CAR T lymphocytes: 1) ability to induce deep molecular remission in case of chemo-resistant ALL in children and adults; 2) ability to persist for many months and carry out massive expansion in vivo; 3) ability to cause prolonged depletion of normal B lymphocytes; 4) ability to cause cytokine release syndrome (see below); 5) need for lymph-depleting chemotherapy prior to administration of CD19 CAR T lymphocytes.
Based on the first successful experience of CD19 CAR T lymphocyte clinical use, pilot studies were initiated in children and adults with recurrent and refractory ALL. Key parameters of the main studies are summarised in Table 1 (Appendix).

CD19 CAR T lymphocytes equipped with chimeric receptors of the second generation, containing 4-1BB signal, were used in all protocols sequences (UPenn/Children's hospital of Philadelphia, Seattle Children's Hospital) or CD28 (Memorial Sloan Kettering Cancer Center, NCI Pediatric branch). The technological approach to production of a therapeutic cellular product was differentiated by the type of vector (lentiviral or retroviral) and composition of the initial cell population. Despite certain differences in production technology of cellular product, the key results of the main studies coincided: incidence of remissions was from 70 to 90%. Hematologic remission was accompanied by deep molecular remission in most patients. The rate of hematologic remission was from 14 to 30 days. The incidence of relapse was 30-50%. The probability of

remission did not depend on the initial tumour mass or previous allogenous transplantation of hematopoietic stem cells.
Clinical studies of various groups differed in terms of the approach to therapy after remission. The percentage of patients who received an allogenous HSCT after remission varied from 10 to 80%. Taking into account the heterogeneity of the patients' sample and absence of a standardised approach to HSCT implementation, it is currently impossible to make a conclusion about optimal tactics of therapy after remission. The published data allow to claim that CD19 CAR T therapy ensures remission lasting more than 2 years without additional consolidation therapy in some patients. According to the data of published studies, 1-year event-free survival is from 30 to 50%. Full data after follow-up for more than 1 year has not been currently published.

Toxicity

Potential toxicity of therapy using autologous CD19 CAR-T lymphocytes observed in clinical studies includes four groups of side effects:

1. “on-target/off-tumour" toxicity caused by specific destruction of normal B lymphocytes expressing CD19 antigen
2. toxicity due to nonspecific effects of activation of a large mass of immune effector cells – cytokine release syndrome (CRS) and toxic encephalopathy, also referred to as CRES (CAR-T related encephalopathy syndrome)
3. toxicity due to immune response to chimeric protein – allergic reactions and CAR-T lymphocyte inactivation
4. insertion oncogenesis

B-cell aplasia

Depletion of normal B lymphocytes is a natural consequence of cytotoxic effect of CD19 CAR-T lymphocytes on a molecular target. B-lymphopenia is accompanied by hypogammaglobulinemia and persists throughout the persistence period of CD19 CAR-T lymphocytes. Extended hypogammaglobulinemia is associated with the risk of developing infectious complications typical for humoral immunodeficiency, and requires replacement therapy with intravenous

immunoglobulin drugs. B-lymphocyte count in the blood can be used as an indirect marker of persistence and functional competence of CD19 CAR-T lymphocytes.

Cytokine release syndrome

Cytokine release syndrome (CRS) is a complex of symptoms accompanying systemic hypercytokinemia associated with uncontrolled activation of the immune system and coinciding with the peak of activation and expansion of CAR-T lymphocytes. Cytokine release syndrome develops during the period from day 0 to day 28 after CD19 CAR-T lymphocyte infusion, and the most severe forms usually develop within three days after CAR-T lymphocyte infusion. The main manifestations of SRS are high fever, myalgia, weakness, tachycardia, arterial hypotension, hypoxemia, respiratory insufficiency, edematous syndrome, rash, coagulopathy, cytolysis, and azotaemia. Severe cases of SRS are accompanied by development of distributive or cardiogenic shock and secondary hemophagocytic syndrome. Cytokine release syndrome is based on hyperproduction of cytokines and chemokines by CAR-T lymphocytes and other immune response effectors, including activated monocytes and macrophages. The most significant CRS drivers include γ-interferon, interleukin-6, TNF-α, and others. Along with proinflammatory cytokines, high concentration of acute phase proteins, such as C-reactive protein and ferritin, and hepatic cytolysis markers: LDH, ALT, AST, are registered in the blood plasma of the patients with CRS. The incidence of CRS is 30-60%, according to different studies, and correlates with the residual tumour mass at the time of CAR-T lymphocyte infusion. Severity of CRS varies from isolated fever, which resolves spontaneously, to development of multiple organ failure. Assessment CRS severity required for selection of therapy is performed according to the modified NCT CTCAE scale. Severe forms of CRS threaten patients' life and require immunosuppressive therapy. The most effective drugs ensuring CRS control are interleukin-6 receptor antagonists – Tocilizumab and corticosteroids. Tocilizumab is a monoclonal antibody binding to the membranous and circulating form of interleukin-6 receptor and blocking its interaction with interleukin-6. Tocilizumab administration in most patients with CRS quickly corrects fever and results in resolution of all clinical and laboratory manifestations of CRS in a short time. Systemic therapy with corticosteroids also ensures CRS control effectively, with a slightly larger interval. Due to the fact that prolonged therapy with corticosteroids can theoretically lead to CAR-T lymphocyte inactivation and loss of therapeutic effect, most clinicians prefer Tocilizumab as the first-line therapy for CRS. Corticosteroids are used in severe and refractory forms of CRS, and in simultaneous development of neurotoxicity, as Tocilizumab penetration through the blood-brain barrier is limited by the size of the molecule.

Neurotoxicity

Symptoms of nervous system damage develop in 10-50% of patients receiving CAR-T lymphocyte therapy in early clinical trials. Neurologic impairment may include such manifestations as headache, seizures, facial nerve paresis, aphasia, apraxia, impaired consciousness, hallucinations, ataxia, tremor, somnolence and, in extreme cases, coma. The development mechanisms of neurotoxic CAR-T therapy effects are not fully studied. It is suggested that an increased concentration of pro-inflammatory cytokines in the cerebrospinal fluid may be the driver of neurotoxic effects. The development of neurotoxicity may coincide in time with the clinical manifestations of CRS, but it may also precede it or develop after the resolution of CRS. There are currently no reliable clinical or laboratory predictors for neurotoxicity development. Neurotoxicity is reversible in the large majority of patients. When selecting the therapy of pronounced side effects from the central nervous system, most clinicians prefer Dexamethasone due to good penetration of the drug in the central nervous system, unlike Tocilizumab.

Allergy and CAR-T lymphocyte inactivation

The chimeric antigenic receptor includes a fragment of a mouse antibody to CD19 antigen, which is a derivative of FMC63 clone. A heterologous sequence can induce an immune response leading to formation of human anti-mouse antibodies or T-cell response. The literature describes separate cases of immune response to chimeric protein, leading to an allergic reaction in case of repeated administration of CD19 CAR-T lymphocytes and/or inactivation of therapeutic cells. Considering the level of previous immunosuppression in the cohort of patients with recurrent/refractory ALL and automatic targeting of the pool of normal B lymphocytes, the probability of developing this type of reactions is negligible.

Insertion oncogenesis

Tumour development due to oncogene activation as a result of accidental transgene incorporation in the corresponding regions of the genome has been described in a number of studies on gene therapy for hereditary immunodeficiencies. In these studies, retroviral vectors of the early generations were used, and the target cell population was represented by hematopoietic stem cells. The experience of mature T lymphocyte transduction using modern retroviral and lentiviral

vectors, including more than 1,000 patients with follow-up period from 1 to 10 years, did not reveal any case of leukaemia development caused by insertional mutagenesis. Thus, this risk is currently minimal and does not contradict the use of this type of genetic modification in treatment of patients with recurrent/refractory ALL.

Unresolved issues

Relapses of acute lymphoblastic leukaemia

Mechanism of relapse development

The follow-up of patients who received CD19 CAR T therapy revealed two main patterns of ALL relapses: 1) CD19-negative relapses: this type of relapse involves the expansion of leukemic population that does not carry CD19 antigen on the surface. The mechanism of CD19 expression loss is partially deciphered and may be associated with alternative splicing of CD19 mRNA resulting in loss of exon 2 and the epitope encoded by it. Another mechanism of CD19 expression loss is associated with CD81. 2) CD19-positive relapses: this type of relapse accompanies the discontinuation of CD19 CAR T-cell persistence as a result of pool exhaustion (more typical for constructions with CD28 co-stimulatory domain and retroviral vectors) or occurrence of anti-CAR immune response. There are also potential additional tumour escape mechanisms associated with the activity of immunoregulatory populations, such as Treg and MDSC (myeloid-derived suppressor cells) and expression of inhibitory immunoreceptor ligands on the surface of tumour cells.

Prognostics and relapse prevention

According to public reports, 30-50% of patients who achieved remission after CD19 CAR-T lymphocyte therapy have relapse of the disease within 1 year. The risk of relapse may vary depending on the CD19 CAR construction used (4-1-BB signal domain ensures a longer persistence of CD19 CAR-T as compared with CD28 signal domain) and general treatment strategy, in particular the approach to hematopoietic stem cell transplantation. Accurate prognostics of relapses is currently difficult.

Toxicity

The key unresolved issue is the optimal approach to prevention of severe side effects. Considering the accumulated data on insignificant role of interleukin-6 in cytotoxic effects of CD19 CAR-T lymphocytes, preventive administration of Tocilizumab is planned in this protocol. The principles of monitoring and therapy in case of side effects are outlined below.

Positioning of CD19 CAR-T therapy in the context of programmed ALL therapy in children and adults

The place of CD19 CAR-T lymphocyte therapy in ALL treatment is not defined. This type of therapy is currently the most effective way of achieving remission in patients with chemo-resistant ALL. Data from earlier studies suggest that the part of the achieved remission is long. The issue of the need to perform allogenous transplantation of hematopoietic stem cells to all patients after remission and of the possibility to replace alloHSCT with CD19 CAR-T therapy in the group of patients with high-risk ALL is still unresolved. According to this protocol, standard therapy after achieving remission is allogenous transplantation of hematopoietic stem cells.

Purposes and objectives of the study

Purpose of the study

Study the efficacy and safety of autologous CD19 CAR T therapy (biomedical cellular product MB CAR-T CD19) in children and young adults with recurrent and refractory course of acute lymphoblastic leukaemia

Objectives of the study

1. Study the safety of autologous CD19 CAR T therapy in children and young adults with recurrent and refractory course of acute lymphoblastic leukaemia based on prospective

assessment of incidence and severity of side effects pursuant to the standard CTCAE criteria v.

2. Study the efficacy of autologous CD19 CAR T lymphocyte therapy in children and young adults with recurrent and refractory course of acute lymphoblastic leukaemia based on assessment of probability of complete hematologic and molecular remission in 28 days after cellular product infusion.
3. Study the long-term efficacy of autologous CD19 CAR T lymphocyte therapy in children and young adults with recurrent and refractory course of acute lymphoblastic leukaemia based on assessment of the overall and event-free survival in 1 year and three years after cellular product infusion.

Criteria for patients' inclusion in the study

Inclusion criteria

Criteria for patients' inclusion in the study

1. Informed consent signed by the patient (aged from 14 to 25 years) and his legal representative (aged from 0 to 18 years).
2. B-lineage acute lymphoblastic leukaemia, disease status complies with one of the following criteria:
    a. Induction failure (primary refractory course), absence of clinical and hematologic remission after the 2nd chemotherapy block or MRD ≥0.1% after the 3rd chemotherapy block in high-risk patients
    b. Early bone marrow or combined relapse, absence of remission or MRD ≥0.1% after 2 blocks of therapy (chemo- or immunotherapy).
    c. Relapse or MRD ≥ 0.1% after allogenous hematopoietic stem cell transplantation
    d. Second and further relapses, absence of remission after the 1st course of therapy (chemo- or immunotherapy) or MRD ≥0.1%
    e. Late bone marrow or combined relapse, absence of clinical and hematologic remission after the 2nd block of therapy (chemo- or immunotherapy) or MRD ≥0.1% after the 3rd chemotherapy block
3. Absence of alternative curative treatment methods

4. Presence of a measurable mass of tumour cells in the bone marrow at the time of patient's inclusion in the study
5. Expression of CD19 antigen by ≥ 30% of tumour cell population
6. Karnofsky or Lansky index over 50%
7. Life expectancy at least 4 weeks
8. Absolute CD3+ lymphocyte count in the peripheral blood is more than 100 in µl
9. Heart function: ejection fraction at least 40%

Exclusion criteria

1. CD19 expression level on the surface of tumour cells is <30%
2. Acute/active hepatitis B, C or acute HIV infection
3. Hypoxemia with SaO2 <90%
4. Bilirubin> 3-x norm
5. Creatinine > 3-x norm
6. Acute GVHD ≥ stage 2
7. Chronic GVHD requiring systemic corticosteroid therapy
8. Pregnancy and breastfeeding
9. Sepsis or other severe uncontrolled infection
10. Severe (>stage 3) CNS pathology (epilepsy, dementia, organic CNS damage)
11. CD3+ (T) lymphocyte count in the peripheral blood is less than 100 in µl
12. Absolute contraindications or technical inability to perform leukocytopheresis

Characteristics of the autologous medical cellular product MB CART 19.1

Chimeric receptor structure

A chimeric antigenic receptor based on the antigen-recognition part of FMC63 and co-stimulatory fragment of 4-1BB will be used in this study. The construction encoding the chimeric antigenic receptor for CD19 (designated as LTI 1563) is structurally very similar to the widely used construction, that was described previously, encoding a chimeric antigenic receptor for CD19 (Porter et al. 2011, Grupp et al. 2013, Maude et al. 2014, Lee et al. 2015, Porter et al. 2015. The part binding to the target is a single-stranded variable fragment of antibodies to human CD19 produced by FMC63 clone of mouse hybridoma, producing antibodies to human CD19, as previously described (Nicholson et al. 1997). The

sequence is bound to the loop region of the human CD8 antigen, costimulatory domain of the CD137/4-1BB protein gene and CD3 antigen signal domainζ. The only difference between the construction MB CD19 CAR 1563 and the widely used construction CD19 CAR (in this case, construction 1538 with the published sequence (Imai et al. (Imai et al. 2004)) is the structure of the transmembrane domain: in our construction, the sequence of the transmembrane domain of human CD8a antigen is replaced by the sequence of another signal molecule of T-lymphocytes, called the protein of the tumour necrosis factor receptor superfamily 19 (TNFRSF19).

Figure 1: Structure of the constructions encoding chimeric antigen receptors compared in preclinical studies.

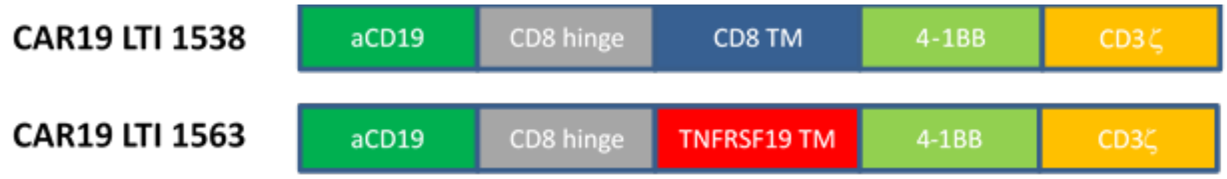

Hinge

General plan of medical intervention

General plan of medical intervention is summarised in Figure

The main stages of the therapy with autologous CAR-T lymphocytes includes the following steps:

1) Leukocytapheresis
2) Cycle of automated production of the biomedical cell product
3) Lymphodepleting chemotherapy
4) Autologous cell product infusion
5) Prevention and treatment of infectious and immunological complications
6) Control studies

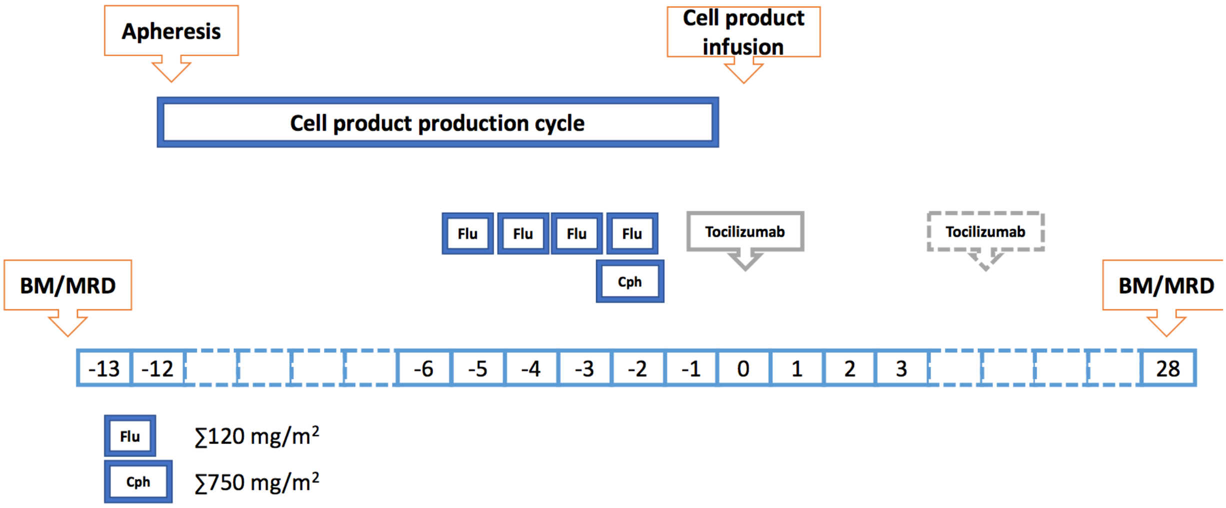

Method of sampling and selection of initial cell population

After a patient is included in the study, mechanical apheresis of peripheral blood mononuclear cells will be performed. The target indicator for the content of T-lymphocytes in the apheresis product (initial material) is at least $50x10^6$ T-lymphocytes (CD3+). Apheresis should be preferably performed prior to chemotherapy using nucleoside analogs to avoid deep depletion of T lymphocytes. The presence of circulating blasts in peripheral blood is not a reason for refusal to perform apheresis.

The first step in the automated cell product production process will be the positive selection of T lymphocytes based on the expression of CD4 and CD8. As a result, the initial culture will be purified from leukaemia blasts and monocytes, and the optimal initial density of lymphocytes in culture is ensured.

Transduction method

A lentiviral vector of the third generation will be used in this protocol for T-lymphocyte transduction. Due to the use of self-inactivating lentiviral vectors, a system of 4 plasmids for their production and minimisation of the intersection of vector sequences and packing constructions, the risk of formation of lentiviral particles capable of replication is minimised. To date, the study of large lots of lentiviruses has not revealed any detectable viral particles (Bear et al. 2012). Only proven lots of lentiviral vector produced according

to GMP standard, which do not contain replicable virus particles, are used for production of MB-CART19.1. In the development of production process, testing for presence of cell-bound and free lentivirus showed that neither free nor cell-bound lentivirus remained in the culture by the 10th day of cultivation. Thus, the risk of transferring the lentiviral particles to the patient and transduction of non-target populations is minimised.

Expansion method

The polyclonal stimulator TransAct (antibodies to CD3 and CD28 immobilised on nanomatrix), interleukin-7 and interleukin-15 will be used for activation and subsequent expansion of CD19 CAR T-lymphocytes. TexMACS medium without the addition of serum will be used as the medium for cultivation. The expansion will be carried out in automatic mode in a closed single-use TS520 system in the CliniMACS Prodigy bioreactor. The duration of production of an autologous cellular product will be 12 days.

Final characteristics of autologous biomedical cell product

The subpopulation of the autologous cell product will be represented by T lymphocytes (CD3 +> 99% of all cells). Viability will be at least 95%. Transduction efficacy will be at least 15%. Negative test for endotoxin, mycoplasma and bacteria and micromycete culture test from +10 days of cultivation.

Quality control in production process

Tests characterising the cellular composition and microbiological safety of the product are planned to be performed in the process of production and final quality control of the biomedical cellular product. The planned tests are summarised in the Table below. A product that has passed quality control pursuant to the established criteria is allowed to be issued.

	Product of apheresis	Enriched fraction (CD4/CD8)	Day 5	Day 10	BMCP	
Panel A (cellular composition, viability)	X	X	X	X	X	
Panel B (transduction efficacy)			X		X	
Endotoxin			X		X	
Mycoplasm				X		
Automatic bacteriologic examination (BactAlert)	X		X	X		
Abundance of vector					X	

Diagnostics and treatment plan

After signing the informed consent and registration, the patient is examined according to the plan stated below:

Plan of the patient's initial examination

- Automatic blood count + leukocyte count
- Biochemical blood test (MACRO biochemistry)
- Coagulogram
- ECG
- Check-up by neurologist
- ECHO cardiography and US dopplerography of the main blood vessels
- External respiratory function
- Peripheral blood immunophenotyping (identification of T and B lymphocyte count)
- Serum immunoglobulins
- Myelogram

- Bone marrow immunophenotyping (blast population (immunophenotype, CD19 expressing blast fraction)
- Minimal residual disease
- Spinal puncture + cytopreparation
- Biobanking (preservation of a tumour sample, DNA, RNA, serum)

Leukocytapheresis

When fulfilling the inclusion criteria, a two-channel central venous catheter will be inserted in an operation room, with a rate corresponding to the requirements for the procedure for leukocytapheresis. The previously inserted catheter can be used provided there is no data suggesting the presence of the catheter-associated infection and thrombosis.

Mechanical leukocytapheresis will be carried out in the medical treatment room of the Blood Transfusion Department using Cobe Spectra or Optia device. The volume of apheresis will be calculated individually based on T-lymphocyte count in the patient's peripheral blood and will range from 0.5 to 1.5 CBV. Leukocytapheresis product will be transferred to the production team (leader Y. Muzalevsky). If the apheresis product corresponds to the necessary initial parameters of the cell composition (the volume should not exceed 250 ml, leukocyte concentration below $1x10^6$/ml, CD3+ lymphocyte count $\geq 50x10^6$) and microbiological safety, the cell production cycle will be initiated pursuant to the regulations (reference to the regulations).

Lymph-depleting chemotherapy

Lymph-depleting therapy is an essential element of autologous CD19 CAR T therapy when using constructions of the second generation. Lymph-depleting therapy will include two drugs: Fludarabine and Cyclophosphamide.

Fludarabine is administered at a course dose of 120 mg/m^2 (30 mg/m^2 on days -5, -4, -3, -2, each injection by intravenous infusion within 30 minutes)

Cyclophosphamide is administered at a dose 750 mg/m^2 (infusion within 1 hour on day -2).

During the chemotherapy the patient will receive infusion therapy in the amount of 2,500 ml/m^2/day, hyperuricemia prevention in the composition of Allopurinol 300 mg/m^2/day (day -4 – day +14), prevention of haemorrhagic cystitis in the composition of uromitexane at a dose of 1,800 mg/m^2/day, daily infusion on day -2, anti-emetic therapy pursuant to the standard adopted in NMRC PHOI.

Synchronisation of lymph-depleting chemotherapy with the production cycle of autologous cell product

In this protocol, a fresh autologous cell product is envisaged to be used as the main option, immediately after the production cycle. The standard production cycle is 12 days, therefore, the first day of the course of lymphodepleting chemotherapy should coincide with the 6th day of the production cycle of an autologous cell product.

Autologous cell product

Dose selection and modification during the study

This study suggests an escalation of the dose of CD19 CAR T lymphocytes pursuant to the pre-established levels. In case of severe side effects at the respective dose level, a dose reduction to the previous level is envisaged for the following patients.

Level 0	$1x10^5$ CD19 CAR T tymphocytes/kg of body mass	
Level 1	$5x10^5$ CD19 CAR T tymphocytes/kg of body mass	5 patients
Level 2	$1x10^6$ CD19 CAR T tymphocytes/kg of body mass	5 patients
Level 3	$3x10^6$ CD19 CAR T tymphocytes/kg of body mass	5 patients

In case of adverse side effects ≥ of grade 4 in 40% (2 of 5) of patients at the respective dose level, the following patients will get CD19 CAR T lymphocytes at a dose of the previous level.

Repeated use of CD19 CAR T tymphocytes

Repeated use of CD19 CAR T tymphocytes us allowed after a patient has reached the assessment period of the main estimated parameters. The condition for repeated use of CD19 CAR T tymphocytes

1) absence of MRD-negative remission on day 28 after the first infusion of CD19 CAR T lymphocytes.
2) preservation of CD19 antigen expression by > 30% of leukemic clone cells.

The decision on the repeated administration of CD19 CAR T lymphocytes, cell dose, regime of lymph-depleting chemotherapy, is made by the protocol group based on clinical feasibility.

Correction of expected side effects

The cytokine release syndrome is the main expected severe adverse effect of CD19 CAR T lymphocyte therapy. This clinical study protocol will implement a strategy for preventive use of Tocilizumab.

Cytokine release syndrome prevention

Tocilizumab

Tocilizumab (Actemra) is a recombinant humanised monoclonal antibody to the interleukin-6 receptor. It binds to the membrane and soluble form of the interleukin-6 receptor and interferes with the interaction of the interleukin-6 receptor with interleukin-6.

The drug will be administered on day 0, 1 hour before infusion of CD19 CAR T lymphocytes, at a dose of 8 mg/kg (maximum dose 800 mg), by intravenous infusion within 1 hour.

Cytokine release syndrome prevention monitoring

Cytokine release syndrome prevention monitoring will include clinical and laboratory monitoring

Clinical monitoring will include measurement of the axillary body temperature every 6 hours, assessment of vital functions every six hours.

Laboratory monitoring will include the following laboratory tests: C-reactive protein and ferritin follow-up daily on days 1-14 after infusion of CD19 CAR T lymphocytes, biochemical blood test (LDH, creatinine, urea, ALT, AST, bilirubin, electrolytes) – once every two days, coagulograms (fibrinogen, APPT, INR) – three times a week.

Diagnostics and assessment of cytokine release syndrome severity

Diagnostics of the cytokine release syndrome will be carried out according to the modified criteria ... Table.

	**Severity**			
**Symptom**	**I**	**II**	**III**	**IV**
Vital signs				
Temperature > 38^0C	Yes	Any	Any	Any
Arterial hypotension	No	Correction by volume or low-dose vasopressors	High doses of vasopressors or >1 drug	Refractory hypotension
Need for oxygen therapy to maintain SaO_2>90%	No	FiO2 < 40%	FiO2 < 40%	Artificial lung ventilation
Organ toxicity				
• Cardiovascular system: tachycardia, arrhythmia, low ejection fraction, block<br>• Respiration: tachypnea, pleural effusion, pulmonary edema<br>• Gastrointestinal tract: nausea, vomiting, diarrhoea<br>• Liver: ALT, AST, bilirubin<br>• Kidneys: oliguria, creatinine<br>• Skin: rash<br>• Coagulopathy: APPT, PI	Degree I	Degree II	Degree III or ALT/AST degree IV	Degree IV, except for ALT/AST

Cytokine release syndrome therapy

If the cytokine release syndrome of degree > 1 develops, the patient will receive therapy of dexamethasone + tocilizumab.

Dexamethasone

0.4 mg/kg once a day for 3 days with assessment of the severity of the condition and dynamics of CRS criteria

Tocilizumab

dose of 8 mg/kg (maximum dose 800 mg), by intravenous infusion within 1 hour.

Intensive care

If intensive care is required, the patient should be transferred to the intensive care unit. Criteria for transfer to the ICU are: 1) arterial hypotension requiring cardiotonics; 2) hypoxemia requiring assisted ventilation; 3) acute renal failure requiring renal replacement therapy; 4) seizures; 5) impaired consciousness

Toxic encephalopathy

Expected manifestations of neurotoxicity

Neurological complications of CD19 CAR T lymphocyte therapy may include the following clinical manifestations: seizures, memory impairment, impaired consciousness, cerebral nerve damage, aphasia, ataxia.

Monitoring of neurological complications will include a daily structural neurological examination pursuant to the form.

In case of toxic encephalopathy, a patient will undergo spinal puncture and MRI of the brain for diagnostic purposes.

Therapy of CAR-T-associated encephalopathy will include Dexamethasone 0.4 mg/kg once a day for 3 days with assessment of the severity of the condition and dynamics of encephalopathy. If

there is no response to Dexamethasone, Kineret (Anakinra) at a dose of 5 mg/kg/day may be used as an anticytokine preparation by the decision of the protocol group and the board of physicians of D. Rogachev NMRC PHOI.

B-cell aplasia and hypogammaglobulinemia

B-cell aplasia is a direct consequence of the effective cytolytic activity of CAR-T lymphocytes against CD19-positive targets. B-lymphopenia is a surrogate marker of the persistence of CAR-T lymphocytes and may persist for 2 weeks up to > 1 year. Hypogammaglobulinemia is the consequence of B-lymphopenia. It is planned to monitor the concentration of serum immunoglobulins at intervals once every three weeks under this protocol. If the concentration of serum immunoglobulins is reduced to <5 g/l, a replacement therapy with an immunoglobulin drug (i/v or s/c) at a dose of 0.4 g/kg of the patient's body mass will be provided.

Allergy and CAR-T lymphocyte inactivation

The chimeric receptor contains fragments of the mouse immunoglobulin structure that may be immunogenic and may induce a cellular and humoral immune response. Such an immune response may lead to development of allergic sensitisation, and to mediate the elimination of CAR-T lymphocyte effector population. Rare cases of allergic reactions and CAR-T lymphocyte inactivation caused by the recipient's immune response were registered in clinical studies. Considering the rare occurrence of allergic reactions, this protocol provides for standard precautions, including presence of anti-shock positioning and access to an oxygen source in the room on the day of CAR-T lymphocyte administration.

Neutropenic fever and approach to infection control

After the course of lymph-depleting therapy with Fludarabine and Cyclophosphamide (see above), neutropenia is expected to last up to three weeks. During the neutropenia period, the procedure of infection prevention and therapy will be observed pursuant to the local protocol for patients with high-risk neutropenia. Considering the combined nature of the emerging immunodeficiency condition, the approach to infection control will correspond to the approach in case of auto-HSCT. Preventive pharmacotherapy will include the following drugs:

- Valaciclovir day 0 - +30

- Trimethoprim/sulphometoxazole day 0 – before recovery of CD4 T lymphocytes> 200/µL
- Fluconazole day 0 – before recovery of granulocytes> 500/µL
- The granulocyte colony-stimulating factor may be prescribed by the decision of the clinical team taking into account the duration of neutropenia and severity of infectious complications

Tactics of hematopoetic stem cell transplantation

The potential of CAR-T lymphocyte therapy in terms of the possibility of curing recurrent and refractory B-linear ALL is not currently fully studied. According to the published data, the incidence of relapses after CAR-T lymphocyte therapy is 30 to 50%. However, it is obvious that CAR-T lymphocyte therapy induces a prolonged unsupported remission in some patients, possibly corresponding to recovery. At the time of this study, allogenous transplantation of hematopoietic stem cells remains the standard therapy for the group of patients with early first relapses, recurrent relapses and refractory forms of ALL. Therefore, all patients included in this protocol are considered candidates for TSCC. The selection of the donor and conditioning composition is carried out pursuant to the current HSCT protocol in D. Rogachev NMRC PHOI. The decision to carry out transplantation is made based on the balance of the expected benefit and risk of the procedure. Transplantation may be performed on day + 45 after the date of CAR-T lymphocyte administration at the earliest.

Clinical and laboratory monitoring plan

Clinical and laboratory monitoring plan is summarised in table.

General clinical care and monitoring

At the time of the course of lymphoblastic chemotherapy and until the recovery of peripheral blood indicators, the patient is admitted to NMRC PHOI hospital. Follow-up after recovery of peripheral blood indicators can be performed in short-stay facility of NMRC PHOI. Clinical monitoring includes

- Examination by attending physician, including structural neurologic examination, – daily
- Vital function control (heart rate, breathing rate, temperature, nBP, SaO2) – every 6 hours
- Body mass control – every 12 hours (more often – in case of clinical necessity)
- Fluid balance control – every 12 hours (more often – in case of clinical necessity)

General laboratory monitoring

Peripheral blood sample is collected daily, starting from the day of the beginning of the course of lymphocytic decongestion until the neutrophil count > $0.5x10^9$/L for three days and platelets > $20x10^9$/L for three days without thrombo concentrate transfusion. Blood is collected for additional and special studies within the decreed term (see table). General laboratory monitoring includes

- Automatic blood test – daily, until peripheral blood indicators are recovered, then twice a week
- Biochemical blood test – once every two days
- Acid-base balance and electrolytes (venous blood) – once every two days
- C-reactive protein – daily
- Coagulogram – 3 times a week
- Myelogram on day 28,

CAR-T lymphocyte persistence monitoring

CAR-T lymphocyte persistence in peripheral blood is assessed on the basis of flow cytometry stained by chimeric antigen receptor detection reagent on T-lymphocyte surface (laboratory monitoring protocol of the laboratory).

Immunological monitoring

Immunological monitoring includes

- peripheral blood lymphocyte subpopulation control
- serum immunoglobulin concentration control
- blood plasma cytokin control

Minimal residual disease monitoring

The minimal residual disease is the key parameter of CAR-T lymphocyte therapy efficacy. In this study, minimal residual disease monitoring will be performed by the following methods:

- Flow cytometry (laboratory of cellular immunology, Popov A. M.)
- TCR gene and immunoglobulin gene rearrangement by sequencing of the next generation (laboratory of cytogenetics and molecular genetics, Olshanskaya Y. V.)
- Real-time PCR (for patients with known chromosomal rearrangement and a verified molecular target for monitoring) (laboratory of cytogenetics and molecular genetics, Olshanskaya Y. V.)

MRD examination will be performed within the established time limit (see table) in bone marrow aspirate.

Cellular composition of cerebrospinal fluid

Cerebrospinal fluid will be collected on days to monitor the status of the central nervous system leukaemia and to study CAR T lymphocyte migration and persistence in the cerebrospinal fluid under the present study.

Biobanking

To preserve the possibility of carrying out additional biological studies, this protocol envisages biobanking of mononuclear cells from peripheral blood and serum, and mononuclear cells of the bone marrow according to table.

Endpoints

Primary endpoints

The main parameter for assessment in this study is the safety and efficacy of autologous CAR T lymphocyte therapy

Safety

- Incidence of side effects of grade ≥ 3 within 1 month after CD19 CAR lymphocyte infusion

Efficacy

- Incidence of clinical haematological remission
- Incidence of MRD-negative remission
- Rate of patients with MRD-negative remission

Additional endpoints

- Incidence of cytokine release syndrome of grade ≥3 within 1 month after CD19 CAR T lymphocyte infusion
- Incidence of toxic encephalopathy of grade ≥ 3 within 1 month after CD19 CAR lymphocyte infusion
- Duration of B-cell aplasia
- General and event-free survival in 1 and 3 years after CD19 CAR T lymphocyte therapy
- CD19 CAR T lymphocyte persistence

Statistical plan of the study

Planned sample size

It is planned to include 18 patients in the study with the possibility of increasing the number to 20 patients. The size of the sample is based on the hypothesis that the difference in probability of achieving an MOB-negative remission in the group with standard chemotherapy (historical control) and CD19-CAR T-cells (intervention) is 0.5 (0.3 vs. 0.8, respectively). When calculating using the chi-square criterion, the required number of patients for the first type of error $\alpha = 0.05$ (bilateral) and power 80% is 18 patients, with the power 90% – 23.

Safety precautions for study participants

Safety monitoring procedure is envisaged in the present protocol to ensure the safety of the study participants. The Safety Monitoring Board is established as follows to ensure safety monitoring:

Zhiganova T. V.
Pashanov E. D.

The Safety Monitoring Board reviews within 5 days after the established time (day 30, day 100 and day 365 after CAR T lymphocyte infusion) the registration form No., containing the report on registered side effects which were registered during the relevant period. Considering the incidence of undesirable side effects of grade 3-4 in the completed clinical trials of similar cell products (up to 80%) and based on the expected cumulative incidence of transplantation mortality in this population of patients of 10%, the study shall be suspended in case of treatment-related undesirable side effects of grade 5 (patient's death) in 20% of patients (> 1 of the first 5 patients,> 2 of the first 10 patients). Death caused by microbiologically documented sepsis/septic shock in a patient with haematopoiesis aplasia shall not be considered as related to CAR T lymphocyte therapy.

Use of data obtained during the study

This study is conducted by D. Rogachev NMRC PHOI in cooperation with the company Miltenyi Biotec. The scientific results of the clinical study are the property of D. Rogachev NMRC PHOI. Prior to the publication of the study results D. Rogachev NMRS PHOI shall inform the company Miltenyi Biotec about the main results at least 60 days before publication. A package of minimal essential clinical data on the study participants' status shall be transferred to the company Miltenyi Biotec according to the following schedule:

1. Bone marrow morphology at the time of inclusion in the study and on day +28 (no later than 14 days after the study)
2. Date of biomedical cell product infusion (within 3 days after the date of infusion)
3. Side effects (within 14 days after the date of registration)

    a. Cytokine release syndrome (of any severity)
    b. Toxic encephalopathy (of any severity)
    c. Secondary tumours
    d. Severe infections (grade 3-5)
    e. All-cause mortality
4. Relapse/progression of acute leukaemia (within 14 days after the date of registration)
5. Persistence and phenotypeMB-CART19.1 (6, 12, 24 months)
6. Remission status (6, 12, 24 months)
7. Absolute B lymphocyte count and immunoglobulin level (6, 12, 24 months)

Reference list

Tedder, T. F., L. J. Zhou and P. Engel (1994). The CD19/CD21 signal transduction complex of B lymphocytes.Immunol Today. 15: 437-42.

Cooper, L. J., Z. Al-Kadhimi, D. DiGiusto, M. Kalos, D. Colcher, A. Raubitschek, S. J. Forman and M. C. Jensen (2004). Development and application of CD19-specific T cells for adoptive immunotherapy of B cell malignancies.Blood Cells Mol Dis. 33: 83-9.

Kalos, M., B. L. Levine, D. L. Porter, S. Katz, S. A. Grupp, A. Bagg and C. H. June (2011). T cells with chimeric antigen receptors have potent antitumor effects and can establish memory in patients with advanced leukemia.Sci Transl Med. 3: 95ra73.

Porter, D. L., B. L. Levine, M. Kalos, A. Bagg and C. H. June (2011). Chimeric antigen receptor-modified T cells in chronic lymphoid leukemia.N Engl J Med. 365: 725-33.

Brentjens, R. J., M. L. Davila, I. Riviere, J. Park, X. Wang, L. G. Cowell, S. Bartido, J. Stefanski, C. Taylor, M. Olszewska, O. Borquez-Ojeda, J. Qu, T. Wasielewska, Q. He, Y. Bernal, I. V. Rijo, C. Hedvat, R. Kobos, K. Curran, P. Steinherz, J. Jurcic, T. Rosenblat, P. Maslak, M. Frattini and M. Sadelain (2013). CD19-targeted T cells rapidly induce molecular remissions in adults with chemotherapy-refractory acute lymphoblastic leukemia.Sci Transl Med. 5: 177ra38.

Grupp, S. A., M. Kalos, D. Barrett, R. Aplenc, D. L. Porter, S. R. Rheingold, D. T. Teachey, A. Chew, B. Hauck, J. F. Wright, M. C. Milone, B. L. Levine and C. H. June (2013). Chimeric antigen receptor-modified T cells for acute lymphoid leukemia.N Engl J Med. 368: 1509-18.

Kochenderfer, J. N., M. E. Dudley, R. O. Carpenter, S. H. Kassim, J. J. Rose, W. G. Telford, F. T. Hakim, D. C. Halverson, D. H. Fowler, N. M. Hardy, A. R. Mato, D. D. Hickstein, J. C. Gea-Banacloche, S. Z. Pavletic, C. Sportes, I. Maric, S. A. Feldman, B. G. Hansen, J. S. Wilder, B. Blacklock-Schuver, B. Jena, M. R. Bishop, R. E. Gress and S. A. Rosenberg (2013). Donor-derived CD19-targeted T cells cause regression of malignancy persisting after allogeneic hematopoietic stem cell transplantation.Blood. 122: 4129-39.

Davila, M. L., I. Riviere, X. Wang, S. Bartido, J. Park, K. Curran, S. S. Chung, J. Stefanski, O. Borquez-Ojeda, M. Olszewska, J. Qu, T. Wasielewska, Q. He, M. Fink, H. Shinglot, M. Youssif, M. Satter, Y. Wang, J. Hosey, H. Quintanilla, E. Halton, Y. Bernal, D. C. Bouhassira, M. E. Arcila, M. Gonen, G. J. Roboz, P. Maslak, D. Douer, M. G. Frattini, S. Giralt, M. Sadelain and R. Brentjens (2014). Efficacy and toxicity management of 19-28z CAR T cell therapy in B cell acute lymphoblastic leukemia.Sci Transl Med. 6: 224ra25.

Maude, S. L., N. Frey, P. A. Shaw, R. Aplenc, D. M. Barrett, N. J. Bunin, A. Chew, V. E. Gonzalez, Z. Zheng, S. F. Lacey, Y. D. Mahnke, J. J. Melenhorst, S. R. Rheingold, A. Shen, D. T. Teachey, B. L. Levine, C. H. June, D. L. Porter and S. A. Grupp (2014). Chimeric antigen receptor T cells for sustained remissions in leukemia.N Engl J Med. 371: 1507-17.

Lee, D. W., J. N. Kochenderfer, M. Stetler-Stevenson, Y. K. Cui, C. Delbrook, S. A. Feldman, T. J. Fry, R. Orentas, M. Sabatino, N. N. Shah, S. M. Steinberg, D. Stroncek, N. Tschernia, C. Yuan, H. Zhang, L. Zhang, S. A. Rosenberg, A. S. Wayne and C. L. Mackall (2015). T cells expressing CD19 chimeric antigen receptors for acute lymphoblastic leukaemia in children and young adults: a phase 1 dose-escalation trial.Lancet. 385: 517-28.

Kochenderfer, J. N., M. E. Dudley, S. H. Kassim, R. P. Somerville, R. O. Carpenter, M. Stetler-Stevenson, J. C. Yang, G. Q. Phan, M. S. Hughes, R. M. Sherry, M. Raffeld, S. Feldman, L. Lu, Y. F. Li, L. T. Ngo, A. Goy, T. Feldman, D. E. Spaner, M. L. Wang, C. C. Chen, S. M. Kranick, A. Nath, D. A. Nathan, K. E. Morton, M. A. Toomey and S. A. Rosenberg (2015). Chemotherapy-refractory diffuse large B-cell lymphoma and indolent B-cell malignancies can be effectively treated with autologous T cells expressing an anti-CD19 chimeric antigen receptor.J Clin Oncol. 33: 540-9.

Turtle, C. J., L. A. Hanafi, C. Berger, T. A. Gooley, S. Cherian, M. Hudecek, D. Sommermeyer, K. Melville, B. Pender, T. M. Budiarto, E. Robinson, N. N. Steevens, C. Chaney, L. Soma, X. Chen, C. Yeung, B. Wood, D. Li, J. Cao, S. Heimfeld, M. C. Jensen, S. R. Riddell and D. G. Maloney (2016). CD19 CAR-T cells of defined CD4+:CD8+ composition in adult B cell ALL patients.J Clin Invest. 126: 2123-38.

Turtle, C. J., L. A. Hanafi, C. Berger, M. Hudecek, B. Pender, E. Robinson, R. Hawkins, C. Chaney, S. Cherian, X. Chen, L. Soma, B. Wood, D. Li, S. Heimfeld, S. R. Riddell and D. G. Maloney (2016). Immunotherapy of non-Hodgkin's lymphoma with a defined ratio of CD8+ and CD4+ CD19-specific chimeric antigen receptor-modified T cells.Sci Transl Med. 8: 355ra116.

Gardner, R. A., O. Finney and C. Annesley (2017). Intent-to-treat leukemia remission by CD19 CAR T cells of defined formulation and dose in children and young adults. 129: 3322-3331.

Turtle, C. J., K. A. Hay, L. A. Hanafi, D. Li, S. Cherian, X. Chen, B. Wood, A. Lozanski, J. C. Byrd, S. Heimfeld, S. R. Riddell and D. G. Maloney (2017). Durable Molecular Remissions in Chronic Lymphocytic Leukemia Treated With CD19-Specific Chimeric Antigen Receptor-Modified T Cells After Failure of Ibrutinib.J Clin Oncol. Jco2017728519.

Scholler, J., T. L. Brady, G. Binder-Scholl, W. T. Hwang, G. Plesa, K. M. Hege, A. N. Vogel, M. Kalos, J. L. Riley, S. G. Deeks, R. T. Mitsuyasu, W. B. Bernstein, N. E. Aronson, B. L. Levine, F. D. Bushman and C. H. June (2012). Decade-long safety and function of retroviral-modified chimeric antigen receptor T cells.Sci.Transl.Med. 4: 132ra53.

Porter, D. L., W. T. Hwang, N. V. Frey, S. F. Lacey, P. A. Shaw, A. W. Loren, A. Bagg, K. T. Marcucci, A. Shen, V. Gonzalez, D. Ambrose, S. A. Grupp, A. Chew, Z. Zheng, M. C. Milone, B. L. Levine, J. J. Melenhorst and C. H. June (2015). Chimeric antigen receptor T cells persist and induce sustained remissions in relapsed refractory chronic lymphocytic leukemia.Sci Transl Med. 7: 303ra139.

Frey, N. V. and S. M. Luger (2015). How I treat adults with relapsed or refractory Philadelphia chromosome-negative acute lymphoblastic leukemia.Blood. 126: 589-96.

Wang, K., G. Wei and D. Liu (2012). CD19: a biomarker for B cell development, lymphoma diagnosis and therapy.Exp Hematol Oncol. 1: 36.

Schuster, S. J., J. Svoboda, S. D. Nasta, D. L. Porter, E. A. Chong, D. J. Landsburg, A. R. Mato, S. F. Lacey, J. J. Melenhorst and A. Chew (2015). Sustained remissions following chimeric antigen receptor modified T cells directed against CD19 (CTL019) in patients with relapsed or refractory CD19+ lymphomas. Am Soc Hematology.

Neelapu, S. S., F. L. Locke and N. L. Bartlett (2016). LBA-6 Kte-C19 (anti-CD19 CAR T Cells) Induces Complete Remissions in Patients with Refractory Diffuse Large B-Cell Lymphoma (DLBCL): Results from the Pivotal Phase 2 ZUMA-1.ASH Abstract.

Brudno, J. N. and J. N. Kochenderfer (2016). Toxicities of chimeric antigen receptor T cells: recognition and management.Blood. 127: 3321-30.

Frey, N. V., B. L. Levine, S. F. Lacey, S. A. Grupp, S. L. Maude, S. J. Schuster, P. Shaw, W.-T. Hwang, M. A. Wasik and A. Obstfeld (2014). Refractory cytokine release syndrome in recipients of chimeric antigen receptor (CAR) T cells. Am Soc Hematology.

Kochenderfer, J. N., W. H. Wilson, J. E. Janik, M. E. Dudley, M. Stetler-Stevenson, S. A. Feldman, I. Maric, M. Raffeld, D. A. Nathan, B. J. Lanier, R. A. Morgan and S. A. Rosenberg (2010). Eradication of B-lineage cells and regression of lymphoma in a patient treated with autologous T cells genetically engineered to recognize CD19.Blood. 116: 4099-102.

Savoldo, B., C. A. Ramos, E. Liu, M. P. Mims, M. J. Keating, G. Carrum, R. T. Kamble, C. M. Bollard, A. P. Gee, Z. Mei, H. Liu, B. Grilley, C. M. Rooney, H. E. Heslop, M. K. Brenner and G. Dotti (2011). CD28 costimulation improves expansion and persistence of chimeric antigen receptor-modified T cells in lymphoma patients.J Clin Invest. 121: 1822-6.

Brentjens, R. J., I. Riviere, J. H. Park, M. L. Davila, X. Wang, J. Stefanski, C. Taylor, R. Yeh, S. Bartido, O. Borquez-Ojeda, M. Olszewska, Y. Bernal, H. Pegram, M. Przybylowski, D. Hollyman, Y. Usachenko, D. Pirraglia, J. Hosey, E. Santos, E. Halton, P. Maslak, D. Scheinberg, J. Jurcic, M. Heaney, G. Heller, M. Frattini and M. Sadelain (2011). Safety and persistence of adoptively transferred autologous CD19-targeted T cells in patients with relapsed or chemotherapy refractory B-cell leukemias.Blood. 118: 4817-28.

Kochenderfer, J. N., M. E. Dudley, S. A. Feldman, W. H. Wilson, D. E. Spaner, I. Maric, M. Stetler-Stevenson, G. Q. Phan, M. S. Hughes, R. M. Sherry, J. C. Yang, U. S. Kammula, L. Devillier, R. Carpenter, D. A. Nathan, R. A. Morgan, C. Laurencot and S. A. Rosenberg (2012). B-cell depletion and remissions of malignancy along with cytokine-associated toxicity in a clinical trial of anti-CD19 chimeric-antigen-receptor-transduced T cells.Blood. 119: 2709-20.

Cruz, C. R., K. P. Micklethwaite, B. Savoldo, C. A. Ramos, S. Lam, S. Ku, O. Diouf, E. Liu, A. J. Barrett, S. Ito, E. J. Shpall, R. A. Krance, R. T. Kamble, G. Carrum, C. M. Hosing, A. P. Gee, Z. Mei, B. J. Grilley, H. E. Heslop, C. M. Rooney, M. K. Brenner, C. M. Bollard and G. Dotti (2013). Infusion of donor-derived CD19-redirected virus-specific T cells for B-cell malignancies relapsed after allogeneic stem cell transplant: a phase 1 study.Blood. 122: 2965-73.

Grupp, S. A., M. Kalos, D. Barrett, R. Aplenc, D. L. Porter, S. R. Rheingold, D. T. Teachey, A. Chew, B. Hauck, J. F. Wright, M. C. Milone, B. L. Levine and C. H. June (2013). Chimeric Antigen Receptor–Modified T Cells for Acute Lymphoid Leukemia.New England Journal of Medicine. 368: 1509-1518.

Lee, D. W., R. Gardner, D. L. Porter, C. U. Louis, N. Ahmed, M. Jensen, S. A. Grupp and C. L. Mackall (2014). Current concepts in the diagnosis and management of cytokine release syndrome.Blood. 124: 188-95.

Dai, H., W. Zhang, X. Li, Q. Han, Y. Guo, Y. Zhang, Y. Wang, C. Wang, F. Shi, Y. Zhang, M. Chen, K. Feng, Q. Wang, H. Zhu, X. Fu, S. Li and W. Han (2015). Tolerance and efficacy of autologous or donor-derived T cells expressing CD19 chimeric antigen receptors in adult B-ALL with extramedullary leukemia.Oncoimmunology. 4: e1027469.

Garfall, A. L., M. V. Maus, W. T. Hwang, S. F. Lacey, Y. D. Mahnke, J. J. Melenhorst, Z. Zheng, D. T. Vogl, A. D. Cohen, B. M. Weiss, K. Dengel, N. D. Kerr, A. Bagg, B. L. Levine, C. H. June and E. A. Stadtmauer (2015). Chimeric Antigen Receptor T Cells against CD19 for Multiple Myeloma.N Engl J Med. 373: 1040-7.

Schuster, S. J., J. Svoboda, S. Dwivedy Nasta, D. L. Porter, E. A. Chong, D. J. Landsburg, A. R. Mato, S. F. Lacey, J. J. Melenhorst, A. Chew, J. Hasskarl, G. D. Shah, M. A. Wasik, K. T. Marcucci, Z. Zheng, B. L. Levine and C. H. June (2015). Sustained Remissions Following Chimeric Antigen Receptor Modified T Cells Directed Against CD19 (CTL019) in Patients with Relapsed or Refractory CD19+ Lymphomas.Blood. 126: 183-183.

Brudno, J. N., R. P. Somerville, V. Shi, J. J. Rose, D. C. Halverson, D. H. Fowler, J. C. Gea-Banacloche, S. Z. Pavletic, D. D. Hickstein, T. L. Lu, S. A. Feldman, A. T. Iwamoto, R. Kurlander, I. Maric, A. Goy, B. G. Hansen, J. S. Wilder, B. Blacklock-Schuver, F. T. Hakim, S. A. Rosenberg, R. E. Gress and J. N. Kochenderfer (2016). Allogeneic T Cells That Express an Anti-CD19 Chimeric Antigen Receptor Induce Remissions of B-Cell Malignancies That Progress After Allogeneic Hematopoietic Stem-Cell Transplantation Without Causing Graft-Versus-Host Disease.J Clin Oncol. 34: 1112-21.

Kebriaei, P., H. Singh, M. H. Huls, M. J. Figliola, R. Bassett, S. Olivares, B. Jena, M. J. Dawson, P. R. Kumaresan, S. Su, S. Maiti, J. Dai, B. Moriarity, M. A. Forget, V. Senyukov, A. Orozco, T. Liu, J. McCarty, R. N. Jackson, J. S. Moyes, G. Rondon, M. Qazilbash, S. Ciurea, A. Alousi, Y. Nieto, K. Rezvani, D. Marin, U. Popat, C. Hosing, E. J. Shpall, H. Kantarjian, M. Keating, W. Wierda, K. A. Do, D. A. Largaespada, D. A. Lee, P. B. Hackett, R. E. Champlin and L. J. Cooper (2016). Phase I trials using Sleeping Beauty to generate CD19-specific CAR T cells.J Clin Invest. 126: 3363-76.

Wang, X., L. L. Popplewell, J. R. Wagner, A. Naranjo, M. S. Blanchard, M. R. Mott, A. P. Norris, C. W. Wong, R. Z. Urak, W. C. Chang, S. K. Khaled, T. Siddiqi, L. E. Budde, J. Xu, B. Chang, N. Gidwaney, S. H. Thomas, L. J. Cooper, S. R. Riddell, C. E. Brown, M. C. Jensen and S. J. Forman (2016). Phase 1 studies of central memory-derived CD19 CAR T-cell therapy following autologous HSCT in patients with B-cell NHL.Blood. 127: 2980-90.

Teachey, D. T., S. F. Lacey, P. A. Shaw, J. J. Melenhorst, S. L. Maude, N. Frey, E. Pequignot, V. E. Gonzalez, F. Chen, J. Finklestein, D. M. Barrett, S. L. Weiss, J. C. Fitzgerald, R. A. Berg, R. Aplenc, C. Callahan, S. R. Rheingold, Z. Zheng, S. Rose-John, J. C. White, F. Nazimuddin, G. Wertheim, B. L. Levine, C. H. June, D. L. Porter and S. A. Grupp (2016). Identification of Predictive Biomarkers for Cytokine Release Syndrome after Chimeric Antigen Receptor T-cell Therapy for Acute Lymphoblastic Leukemia.Cancer Discov. 6: 664-79.

Cai, B., M. Guo, Y. Wang, Y. Zhang, J. Yang, Y. Guo, H. Dai, C. Yu, Q. Sun, J. Qiao, K. Hu, H. Zuo, Z. Dong, Z. Zhang, M. Feng, B. Li, Y. Sun, T. Liu, Z. Liu, Y. Wang, Y. Huang, B. Yao, W. Han and H. Ai (2016). Co-infusion of haplo-identical CD19-chimeric antigen receptor T cells and stem cells achieved full donor engraftment in refractory acute lymphoblastic leukemia.J Hematol Oncol. 9: 131.

Grupp, S. A., T. W. Laetsch, J. Buechner, H. Bittencourt, S. L. Maude, M. R. Verneris, G. D. Myers, M. W. Boyer, S. Rives, B. De Moerloose, E. R. Nemecek, K. Schlis, P. L. Martin, M. Qayed, P. Bader, H. Hiramatsu, F. Mechinaud, G. A. Yanik, C. Peters, A. Biondi, A. Baruchel, N. Boissel, J. Krueger, C. H. June, K. Sen, Y. Zhang, K. E. Thudium, P. A. Wood, T. Taran and M. A. Pulsipher (2016). Analysis of a Global Registration Trial of the Efficacy and Safety of CTL019 in Pediatric and Young Adults with Relapsed/Refractory Acute Lymphoblastic Leukemia (ALL).Blood. 128: 221-221.

Maude, S. L., D. M. Barrett, S. R. Rheingold, R. Aplenc, D. T. Teachey, C. Callahan, D. Baniewicz, C. White, M. K. Talekar, P. A. Shaw, J. L. Brogdon, R. M. Young, J. Scholler, K. T. Marcucci, B. L. Levine, N. Frey, D. L. Porter, S. F. Lacey, J. J. Melenhorst, C. H. June and S. A. Grupp (2016). Efficacy of Humanized CD19-Targeted Chimeric Antigen Receptor (CAR)-Modified T Cells in Children and Young Adults with Relapsed/Refractory Acute Lymphoblastic Leukemia.Blood. 128: 217-217.

Locke, F. L., S. S. Neelapu, N. L. Bartlett, T. Siddiqi, J. C. Chavez, C. M. Hosing, A. Ghobadi, L. E. Budde, A. Bot, J. M. Rossi, Y. Jiang, A. X. Xue, M. Elias, J. Aycock, J. Wiezorek and W. Y. Go (2017). Phase 1 Results of ZUMA-1: A Multicenter Study of KTE-C19 Anti-CD19 CAR T Cell Therapy in Refractory Aggressive Lymphoma.Mol Ther. 25: 285-295.

Kochenderfer, J. N., R. P. T. Somerville, T. Lu, V. Shi, A. Bot, J. Rossi, A. Xue, S. L. Goff, J. C. Yang, R. M. Sherry, C. A. Klebanoff, U. S. Kammula, M. Sherman, A. Perez, C. M. Yuan, T. Feldman, J. W. Friedberg, M. J. Roschewski, S. A. Feldman, L. McIntyre, M. A. Toomey and S. A. Rosenberg (2017). Lymphoma Remissions Caused by Anti-CD19 Chimeric Antigen

Receptor T Cells Are Associated With High Serum Interleukin-15 Levels.J Clin Oncol. 35: 1803-1813.

Qasim, W., H. Zhan, S. Samarasinghe, S. Adams, P. Amrolia, S. Stafford, K. Butler, C. Rivat, G. Wright, K. Somana, S. Ghorashian, D. Pinner, G. Ahsan, K. Gilmour, G. Lucchini, S. Inglott, W. Mifsud, R. Chiesa, K. S. Peggs, L. Chan, F. Farzeneh, A. J. Thrasher, A. Vora, M. Pule and P. Veys (2017). Molecular remission of infant B-ALL after infusion of universal TALEN gene-edited CAR T cells.Sci Transl Med. 9.

Fitzgerald, J. C., S. L. Weiss, S. L. Maude, D. M. Barrett, S. F. Lacey, J. J. Melenhorst, P. Shaw, R. A. Berg, C. H. June, D. L. Porter, N. V. Frey, S. A. Grupp and D. T. Teachey (2017). Cytokine Release Syndrome After Chimeric Antigen Receptor T Cell Therapy for Acute Lymphoblastic Leukemia.Crit Care Med. 45: e124-e131.

Pan, J., J. F. Yang, B. P. Deng, X. J. Zhao, X. Zhang, Y. H. Lin, Y. N. Wu, Z. L. Deng, Y. L. Zhang, S. H. Liu, T. Wu, P. H. Lu, D. P. Lu, A. H. Chang and C. R. Tong (2017). High efficacy and safety of low-dose CD19-directed CAR-T cell therapy in 51 refractory or relapsed B acute lymphoblastic leukemia patients.Leukemia.

Hu, Y., Z. Wu, Y. Luo, J. Shi, J. Yu, C. Pu, Z. Liang, G. Wei, Q. Cui, J. Sun, J. Jiang, J. Xie, Y. Tan, W. Ni, J. Tu, J. Wang, A. Jin, H. Zhang, Z. Cai, L. Xiao and H. Huang (2017). Potent Anti-leukemia Activities of Chimeric Antigen Receptor-Modified T Cells against CD19 in Chinese Patients with Relapsed/Refractory Acute Lymphocytic Leukemia.Clin Cancer Res. 23: 3297-3306.

Table Initial screening

Table Structured neurological examination

Table Criteria for transfer to Intensive Care Unit

Table Cytokine release syndrome diagnostics

Table Laboratory monitoring up to day +30

Table Laboratory monitoring and follow-up plan

Protocol study plan

	**Before CD19 CAR-T infusion**			**After CD19 CAR-T infusion**												
			**Day**							**Month**						
	**Screen**	**apheresis**	**-6**	**0**	**1**	**2**	**7**	**14**	**28**	**2**	**3**	**4**	**6**	**8**	**10**	**12**
Inclusion/exclusion criteria	x															
Informed consent	x															
Medical history	x															
Physical examination	x	x	x	x	x	x	x	x	x	x	x	x	x	x	x	x
Neurological examination	x			x	x	x	x	x	x		x					

Hemogram, blood biochemistry, coagulogram	x	x	x	x	x	x	x	x	x	x	x	x	x	x	x	x
Clinical monitoring of toxicity and side effects	x	Throughout the entire monitoring period														
**Efficacy control**																
Bone marrow and MRD morphology	x								x		x		x			x
Examination of cerebrospinal fluid (cytopreparation)	x								x		x		x			x
**Immunological monitoring**																
IgG serum concentration				x			x	x	x	x	x	x	x	x	x	x
Peripheral blood lymphocyte subpopulation	x			x			x	x	x	x	x	x	x	x	x	x
Persistence and phenotype of CAR T lymphocytes				x		x	x	x	x		x		x			x
Serum cytokines, CRP, ferritin				x	x	x	x	x	x							
Transgene immunogenicity				x							x					
CSF cytokine test, lymphocyte composition,									x		x		x			x
PK biobanking	x			x	x	x	x	x	x		x	x	x	x	x	x
BM biobanking	x								x		x		x			x
CSF biobanking	x								x		(x)		(x)			(x)

Information for the study participants

CAR-T lymphocytes are a new treatment method for acute lymphoblastic leukaemia in children and adults. This method is developed for patients who are unable to achieve remission of the disease using any of the existing methods of drug therapy. This type of leukaemia is called chemoresistant and, until recently, it was considered absolutely incurable. The method is based on genetic engineering and cellular immunology techniques, the use of which allows equipping the patient's own lymphocytes with a special receptor enabling them to recognise leukaemia cells which have a CD19 molecule on their surface. This molecule is found on the surface of tumour cells in the vast majority of patients with acute lymphoblastic leukaemia. Peripheral blood leukocytes are collected from a patient using the mechanical apheresis technique to produce CAR-T lymphocytes in the patient. A patient may need to have a special catheter, however, a normal catheter through which the patient receives chemotherapy is sometimes sufficient. At the second stage the patient's lymphocytes are treated in a special device – bioreactor CliniMACS Prodigy – using special reagents based on lentiviral particles, which incorporate a new receptor into T-lymphocytes. After that, the lymphocytes multiply in the bioreactor for 12 days. The procedure results in formation of several hundred million T-lymphocytes equipped with a new receptor capable of recognising tumour cells. Before injecting CAR-T lymphocytes to the patient, a 4-day chemotherapy course is given; it consists of two drugs: Cyclophosphamide and Fludarabine. These drugs are required to create favourable conditions for antitumour cells in the patient's body. After chemotherapy, the patient is transfused with CAR-T lymphocytes at a dose of about 1 million cells per kilogram of the patient's body mass. When CAR-T lymphocytes get in the patient's blood, they begin to find and destroy tumour cells. Deep remission can be achieved in 90% of patients with ALL one month after CAR-T lymphocyte administration. CAR-T lymphocytes act like true lymphocytes and can persist for

months in the patient's body, continuing to control the tumour. In some patients who received CAR-T lymphocyte therapy remission persists for more than 4 years, and these patients are most likely cured of leukaemia. Approximately 30-50% of patients have recurrent leukaemia, so it is now considered that the best way to maintain remission is hematopoietic stem cell transplantation.

Complications

CAR-T lymphocyte therapy may be accompanied by several types of complications:

1) CD19 molecule is found on tumour cells and on healthy B lymphocytes. CAR-T lymphocytes destroy healthy B lymphocytes along with the tumour. As a result, synthesis of immunoglobulins – antibodies to different germs protecting us from infections – in the patient's body is temporarily discontinued. Fortunately, missing immunoglobulins can easily be replaced by donor immunoglobulin transfusion, until the patient's immune system recovers.

2) When CAR-T lymphocytes attack tumour cells, they produce a huge amount of special signalling substances – cytokines – as in case of a severe infection. This complication is called "cytokine release syndrome" or "cytokine storm". The main manifestation of cytokine release is a very high fever, chills, weakness, muscle aches, poor health. The cytokine release syndrome develops in 50% of patients, usually within the first two weeks after CAR-T lymphocyte administration. In severe cases, the cytokine release syndrome may result in shock and respiratory failure, which require treatment in the intensive care unit. Two drugs Tocilizumab and Dexamethasone are used to prevent and treat cytokine release syndrome. These drugs block the main harmful cytokines and help to avoid the risk of loss of life. The vast majority of patients safely recover from this complication.

3) Another special complication of CAR-T lymphocyte therapy is toxic encephalopathy. This manifestation is also most likely associated with synthesis of cytokines and their effect on the brain. Toxic encephalopathy after CAR-T lymphocyte therapy develops within the first four weeks and may coincide with the cytokine release syndrome or develop on its own. The main manifestations of encephalopathy are somnolence, slowness, impaired speech and coordination, in some cases - seizures. Encephalopathy responds well to Dexamethasone treatment and is completely resolved in the vast majority of patients.
4) CAR-T lymphocytes include a foreign protein that may be recognised by the patient's immune system and cause allergic reactions. Such reactions are very rare when CAR-T lymphocytes are administered, and can be treated in the same way as allergic reactions to other drugs.
5) When using CAR-T lymphocytes, there is a <u>theoretical</u> risk of malignant T lymphocyte transformation and development of secondary leukaemia. To date, no such case has been reported after CAR-T lymphocyte administration in several hundred patients worldwide.

Despite relatively high incidence of complications, the risk of fatal complications is less than 5% in case necessary precautions are observed. Considering the absolute risk of death due to progression in case of chemo-resistant ALL, the expected benefit of CAR-T lymphocyte therapy is many times greater than the risk associated with their administration.

Voluntary informed consent to participation in the study

"Safety and efficiency of anti-CD19 chimeric antigen receptor (CAR)- transduced T-cell therapy for pediatric and young adult patients with relapsed/refractory B-cell acute lymphoblastic leukemia: a single centre, non-randomised, open label phase I-II clinical trial of automatically produced cell therapy product MB CART 19.1 using CliniMACS Prodigy"

I,_________*full name* ____,

passport No. _____ _______________, issued ____, residing at ____,

being the legal representative - mother/father/adopter, guardian/custodian (underline as appropriate) of minor child, ______ *full name*____, MIS ID_____________, undergoing treatment in FSBI NMRC Dmitry Rogachev PHOI of the Russian Ministry of Healthcare (hereinafter referred to as NMRC Dmitry Rogachev PHOI), pursuant to Articles 30, 31, 32 of the Basic Legislative Principles of the Russian legislation on health protection of citizens adopted 22.07.1993, am informed about (a) gist of the disease my child

suffers from: recurrent/chemo-resistent B-linear acute lymphoblastic leukaemia.

I received clear explanations about the options for the course and prognosis of my child's disease, possible options of medical intervention, considering the course of the disease and changes in my child's body.

I am informed that cellular immunotherapy with CAR-T lymphocytes is the most effective method of treating recurrent and refractory B-linear acute lymphoblastic leukaemia, and that this therapy can be performed to my child dd.mm. 201__

I was explained that CD19 CAR-T lymphocyte therapy is a new treatment method for B-linear acute lymphoblastic leukaemia. The method is based on genetic modification of the patient's own lymphocytes, which leads to their acquired ability to attack and destroy cells of B-linear acute lymphoblastic leukaemia, as there is CD19 antigen on their surface. Two cellular drugs based on this technology were registered in 2017 in the USA. Dmitry Rogachev NMRC PHOI together with the German company Miltenyi Biotec are currently conducting a pilot study of the cell product produced in the CliniMACS Prodigy automatic system. The composition and structure of the cell product is similar to the drugs registered in the USA for clinical use.

The purpose of this study is to examine the efficacy and safety of the cell product in the treatment of chemo-resistant ALL in children. The treatment plan consists of 5 stages:

1) Apheresis (collection) of lymphocytes from the patient's blood
2) Production of CD19 CAR-T lymphocytes in automatic bioreactor CliniMACS Prodigy
3) Course of chemotherapy using Fludarabine and Cyclophosphamide
4) CD19 CAR-T lymphocyte infusion
5) Follow-up and control examination of the patient

I was explained that there are two main reasons for failure of CAR-T therapy for acute lymphoblastic leukaemia:

1) Resistance (stability) of acute lymphoblastic leukaemia to CAR-T therapy – observed in 10-15% of patients
2) Relapse of acute lymphoblastic leukaemia after remission – observed in 30% of patients

I was explained that there are three main side effects of CD19 CAR-T therapy

1) Cytokine release syndrome is an immunological reaction associated with the activity of therapeutic cells. The main manifestations are high fever, chills, palpitations, frequent breathing, weakness. Cardiovascular and respiratory failure may develop in severe cases.

Cytokine release syndrome occurs in 30-50% of patients and it can be treated with the drug Tocilizumab in most cases.

2) Toxic encephalopathy is a dysfunction of the central nervous system, which may be manifested by somnolence, impaired speech, coordination and memory, and seizures. Toxic encephalopathy develops in 10-30% of patients and is reversible in most cases. The drug Dexamethasone is necessary in case of severe encephalopathy.
3) B-lymphopenia and hypogammaglobulinemia are a direct consequence of the effective function of CD19 CAR-T lymphocytes. B-lymphocytes are not synthesised while CAR-T lymphocytes are found in the patient's body. During this time, replacement therapy of intravenous immunoglobulin drug is necessary.

I was explained that the following side effects may also develop:

- In the haemopoietin system:thrombocytopenia, neutropenia, anaemia, lymphopenia
- Infectious complications: possible bacterial, viral, fungal infections, including sepsis, pneumonia, enterocolitis
- In the gastrointestinal tract: nausea, vomiting, diarrhoea, constipation

I am informed that there is a theoretical risk of malignant transformation of therapeutic cells, but this risk is less than 0.1%.

I received clear explanations about the expected efficacy of the proposed method, the extent and risk factors for my child, and the activities in case of unforeseen effects on the child's health.

I am forewarned and understand that implementation of this method is related to risks described above and that saving a patient's life can not be achieved without risk.

I am informed that doctors and nurses will assume all possible measures to prevent risks to my child's health when using this method during the proposed treatment.

I am informed that, in order to study the efficacy and safety of CD19 CAR-T lymphocyte therapy, an in-depth study of therapeutic cell activity in the patient's body is necessary after administration and change in the number of tumour cells. Regular collection of blood, bone marrow and cerebrospinal fluid is necessary for this study. I agree to the collection, storage and subsequent study of biosamples.

I understand that the purpose of this new method in NMRC Dmitry Rogachev PHOI is to ensure significant improvement in the outcomes of treatment of recurrent and refractory acute lymphoblastic leukaemia in children and increased chance of curing both my child and other patients with similar disease.

I am informed that I have the right to refuse to participate in the proposed clinical trial and, in case of my refusal, the treatment will be performed in accordance with the standard accepted in the clinic, there will be no restrictions to my child's rights and interests.

I was given the opportunity to ask all questions about the degree of risk and benefit of the proposed treatment method, and the doctor gave me clear and exhaustive answers in an intelligible form.

My signature under this consent means that I have read the text of this consent, I understand everything, I have received satisfactory answers to all questions and I agree to my child's participation in this study.

____ Full name of the parent/guardian

____ Date (to be written by the signatory), signature

Full name of the physician who conducted the discussion of the informed consent

__________________________ _______________________________ date, signature

Clinical Study	Study design	Phase	CAR type/ gene transfer	Number of patients	Cell dose	Control follow-[illegible]
**(Kochenderfer et al. 2010) (registration number: NCT00924326)**	**CD19**<br>• FL (follicular lymphoma)<br><br>preparatory chemotherapy regimen with subsequent administration of autologous T-lymphocytes (preparatory regimen: Cyclophosphamide 60 mg/kg a day for 2 days, then Fludarabine 25 mg/m^2 a day for 5 days)<br>Cytokine support after the second T-lymphocyte infusion: 720000 IU/kg every 8 hours depending on tolerability	I	scFvCD19.CD28-CD3ξ retrovirus	1	1x10^8 T-lymphocytes with chimeric antigenic receptors on Day 1; 3x10^8 T-lymphocytes with chimeric antigenic receptors on Day 2	39 weeks
**(Savoldo et al. 2011) (registration number: not provided)**	**CD19**<br>• non-Hodgkin lymphoma<br>• simultaneous administration of two autologous T-lymphocyte drugs expressing the chimeric antigenic receptors<br>• Without preparatory regimen	no data	scFvCD19.CD3ξ as compared with scFvCD19.CD28-CD3ξ retrovirus	6	2x10^7–2x10^8 T-lymphocytes with chimeric antigenic receptors on 1 m^2<br>1-2 infusions	
**(Brentjens et al. 2011) (registration number: NCT00466531, registration number: NCT01044069)**	**CD19**<br>• 8 patients out of 10 with chronic lymphatic leukaemia<br>• 2 patients out of 10 with acute lymphatic leukaemia<br>• Patients' preparation:<br>• CLL:<br>*Stage 1*: 3 patients out of 8 did not have<br>*Stage 2*: 5 patients out of 8 received Cyclophosphamide<br>(doses: 1.5 and 3.0 g/m^2)<br>ALL: 2 patients out of 2 received Cyclophosphamide<br>(dose: 3.0 mg/m^2)	I/II	scFvCD19.CD28-CD3ξ retrovirus	10	CLL:<br>*Stage 1:*<br>level 1 dose<br>1.2-3.0x10^7 T-lymphocytes with chimeric antigenic receptors on 1 kg of body mass<br>(without prior preparation using Cyclophosphamide)<br>*Stage 2:*<br>Chemotherapy with increase in Cyclophosphamide doses (1.5 and 3.0 g/m^2) with subsequent infusion in 2 days of modified T-lymphocytes with level 1 dose (1.2-3.0x10^7 T-lymphocytes with chimeric antigenic receptors per 1 kg of body mass)<br>(the first case of death of a patient during the study) or infusion of modified T-lymphocytes with level -1 dose<br>0.4-1.0x10^7 T-lymphocytes with chimeric antigenic receptors per 1 kg of body mass (2-3 infusions) with divided dose for administration during 2 days<br>ALL:<br>Cyclophosphamide (3.0 g/m^2) infusion of divided dose of autologous T-lymphocytes in 2 days increase in dose<br>with three pre-planned T-lymphocyte doses: 3x10^6, 1x10^7 and 3x10^7 T-lymphocytes with chimeric antigenic receptors per 1 kg of body mass	15 months
**(Kalos et al. 2011, Porter et al. 2011) (registration number: NCT01029366)**	**CD19**<br>• CLL (chronic lymphatic leukaemia)<br><br>• preliminary therapy with anti-lymphoma chemotherapy drugs 1-4 days before infusions of T-lymphocytes with chimeric antigenic receptors<br><br>Preparatory therapy with Pentostatin 4 mg/m^2 and Cyclophosphamide 600 mg/m^2	I	scFvCD19.4-1BB-CD3ξ lentivirus	3	1.4x10^5/kg – 1.6x10^7/kg	24 months
**(Kochenderfer et al. 2012) (registration number: NCT00924326)**	**CD19**<br>• 4 patients with chronic lymphatic leukaemia<br>• 3 patients with follicular lymphoma<br>• 1 patient with SMZL (splenic marginal zone lymphoma)<br><br>Preparatory regimen: Cyclophosphamide (60 mg/m^2 x2) + Fludaribine (25 mg/m^2 x5) + IL-2	I	scFvCD19.CD28-CD3ξ retrovirus	8	0.3-2.8x10^7/kg	≥15 months

**(Cruz et al. 2013) (registration number: NCT00840853)**	**CD19**<br>• 4 patients out of 8 with acute lymphatic leukaemia<br>• 4 patients out of 8 with chronic lymphatic leukaemia<br>• Infusion of T-lymphocytes with chimeric antigenic receptors within 3 months – 13 years after allogeneic stem cell transplantation<br>Without preparatory regimens	I	scFvCD19.CD28-CD3ξ retrovirus	8	1.5, 4.5, 12 × 10^7 T-lymphocytes with chimeric antigenic receptors per 1 m^2	≥12 weeks
**(Grupp et al. 2013) (registration number: NCT01626495)**	**CD19**<br>• ALL (acute lymphoblastic leukaemia)<br>Preparatory regimen in 1 patient out of 2 was not applied, 1 patient out of 2 received Etoposide in combination with Cyclophosphamide (data about concentrations was not provided)	I/IIa	scFvCD19.4-1BB-CD3ξ lentivirus	2 (children)	0.14-1.2x10^7/kg (single-time)	≥11 months
**(Brentjens et al. 2013) (registration number: NCT01044069)**	**CD19**<br>• ALL (acute lymphoblastic leukaemia)<br>Preliminary preparatory treatment with Cyclophosphamide (doses from 1.5 to 3.0 g/m2)	I	scFvCD19.CD28-CD3ξ retrovirus	5	1.5-3.0x10^6 T-lymphocytes with chimeric antigenic receptors per 1 kg of body mass (the dose is divided for infusion on Day 1 and 2)	45 weeks
**(Kochenderfer et al. 2013) (registration number: NCT01087294)**	**CD19**<br>• 4 patients with chronic lymphatic leukaemia<br>• 2 patients with diffuse large cell B-cell lymphoma<br>• 4 patients with mantle cell lymphoma<br>• All patients had malignant neoplasms that persisted after hematopoietic stem cell allografting and standard infusions of donor lymphocytes<br>• None of the patients received any therapy of preparatory regimen	I	scFvCD19.CD28-CD3ξ retrovirus	10	0.4-7.8x10^6/kg (received from an allogenic donor)	≥11 months
**(Maude et al. 2014) (registration number: NCT01626495 and NCT01029366)**	**CD19**<br>• ALL (acute lymphoblastic leukaemia)<br>• anti-lymphoma chemotherapy 1 week before infusions of T-lymphocytes with chimeric antigenic receptors<br>• individualised preparatory regimen for each patient	I	scFvCD19.4-1BB-CD3ξ lentivirus	30<br>25 patients out of 30 are paediatric<br>5 patients out of 30 are adults	0.76x10^6–20.6x10^6 T-lymphocytes with chimeric antigenic receptors per 1 kg of body mass<br>The dose was administered in parts	The median duration of contr follow-up was 7 months<br>(from 1 to 24 months)

	• none in 3 patients out of 30; in 27 patients out of 30 reserve therapy as selected by attending physician<br>• 5 patients out of 27 had Etoposide 100 mg/m2 per day for 2 days, Cyclophosphamide 440 mg/m2 per day for 2 days<br>• 1 patients out of 27 had Etoposide 150 mg/m2 per day for 1 days, Citarabin 300 mg/m2 per day for 1 days<br>• 13 patients out of 27 had Fludarabin 30 mg/m2 per day for 4 days, Cyclophosphamide 500 mg/m2 per day for 2 days<br>• 2 patients out of 27 had Fludarabin 30 mg/m2 per day for 3 days, Cyclophosphamide 300 mg/m2 per day for 3 days<br>• 1 patient out of 27 had Cyclophosphamide 1,000 mg/m2 per day for 1 days<br>• 1 patient out of 27 had Clofarabine 30 mg/m2 per day for 5 days<br>• 1 patient out of 27 had Methotrexate 1,000 mg/m^2 on day 1, Cytarabine 1,000 mg/m^2 every 12 hours on days 2 and 3<br>• 1 patient out of 27 had Cyclophosphamide 300 mg/m^2 every 12 hours on days 1-3, Vincristine 2 mg on day 3, Adriamycin 50 mg/m^2 on day 3<br>• 2 patient out of 27 had Cyclophosphamide 300 mg/m^2 every 12 hours for 3 days					
**(Davila et al. 2014) (registration number: NCT01044069)**	**CD19**<br>• ALL (acute lymphoblastic leukaemia)<br>• Preparatory chemotherapy: Cyclophosphamide (1.5-3 g/m2)	I	scFvCD19.CD28-CD3ξ retrovirus	16 adult patients	3x106 T-lymphocytes with chimeric antigenic receptors per 1 kg of body mass, 1/3 of dose on Day 1, 2/3 of dose on Day 2	Control follow-u[illegible] during the perio[illegible] from 2 to 24 mo[illegible]
**Lee et al. 2014) (registration number: NCT01593696)**	**CD19**<br>• 20 patients out of 21 suffered from acute lymphatic leukaemia<br>• 1 patient out of 21 had diffuse large cell B-cell lymphoma<br>• study with increase in dose<br>• Preparatory regimen: Fludarabin 25 mg/m2 per day on Days 2, 3 and 4, Cyclophosphamide 900 mg/m^2 on Day 2<br>• 8 patients out of 21 had previously received allogenic hematopoietic stem cell transplantation	I	scFvCD19.CD28-CD3ξ retrovirus	21 paediatric/young adult patients	$1x10^6$ T-lymphocytes with chimeric antigenic receptors per 1 kg of body mass (dose 1), $3x10^6$ T-lymphocytes with chimeric antigenic receptors per 1 kg of body mass (dose 2) or all content of the vial (during one infusion)	Median duration control follow-u[illegible] was 10 months
**(Kochenderfer et al. 2015) (registration number: NCT00924326)**	**CD19**<br>• 4 patients with chronic lymphatic leukaemia<br>• 5 patients with diffuse large cell B-cell lymphoma<br>• 1 patient with highly differentiated non-Hodgkin's lymphoma<br>• 1 patient with splenic marginal zone lymphoma<br>• 4 patients with primary mediastinal cell B-cell lymphoma<br>• Preparatory therapy with single-time infusion of T-lymphocytes with chimeric antigenic receptors every second day<br>• Preparatory therapy: Cyclophosphamide in a combined dose of 120 or 60 mg/kg and five daily administrations of Fludarabine 25 mg/m^2 per day	I	scFvCD19.CD28-CD3ξ retrovirus	15	1–$5x10^6$/kg<br>1-$4x10^6$ T-lymphocytes with chimeric antigenic receptors per 1 kg of body mass	22 months

**(Porter et al. 2015) (registration number: NCT01029366)**	**CD19**<br>• Recurrent or refractory chronic lymphocytic leukaemia<br>• single course of standard antilymphocyte chemotherapy, completed 4 days before the infusion<br>• Patients' preparation:<br>• 3 patients out of 14 received Fludarabine/Cyclophosphamide<br>• 5 patients out of 14 received Pentostatin/Cyclophosphamide<br>• 6 patients out of 14 received Bendamustin (data about concentration is not available)<br>8 patients out of 14 had 17p chromosomal deletion (Tr53)<br>1 patient out of 14 had previously undergone allogenic hematopoietic stem cell transplantation<br>Disease progression in 1 patients out of 14 against the background of Ibrutinib therapy	I	scFvCD19.4-1BB-CD3ξ lentivirus	23 patients included/14 received infusion of at least one dose of CTL019<br>Median age of patients is 66 years (ranging from 51 to 78 years)	$0.14 \times 10^8 – 11 \times 10^8$ T-lymphocytes with chimeric antigen receptors (median 1.6×10^8 cells) (1 to 3 infusions)	53 months
**(Dai et al. 2015) (registration number: NCT01864889)**	**CD19**<br>• ALL (acute lymphoblastic leukaemia)<br>• Three patients had previously received allogenic hematopoietic stem cell transplantation<br>• Patients' preparation:<br>• 7 patients out of 9 did not have<br>• 2 patients out of 9 received C-MOAD therapy: Cyclophosphamide 600 mg/day on Day 1 and 4, Mitoxantrone 5 mg on Day 1, Vindesine 4 mg/day on Day 1, Cytarabine 100 mg/day on Day 1, Dexamethasone 10 mg/day on Day 1-3.	no data	scFvCD19.4-1BB-CD3ξ lentivirus	9	$0.33 \times 10^7 – 1.26 \times 10^7$ T-lymphocytes with chimeric antigenic receptors per 1 kg of body mass (3-5 infusions)	54 weeks
**(Garfall et al. 2015) (registration number: NCT02135406)**	**CD19**<br>• Multiple myeloma<br>• Infusion of cells with chimeric antigenic receptors after chemotherapy suppressing the growth of myelocytes (Melphalan 140 mg/m^2) and autologous stem cell transplantation	I	scFvCD19.4-1BB-CD3ξ lentivirus	1	5×10^7 T-lymphocytes with chimeric antigenic receptors (1 infusion)	12 months
**(Schuster et al. 2015) (registration number: not provided)**<br>***Abstract for ASH***	**CD19**<br>• Recurrent or refractory chronic lymphocytic leukaemia<br>• DLBCL in 21 patients out of 38<br>• FL in 14 patients out of 38<br>• MCL in 3 patients out of 38<br>• Median value of previously received lines of therapy was 4 (from 1 to 10)<br>• 12 patients out of 38 (32%) underwent transplantation (11 patients out of 12 underwent autologous stem cell transplantation and 1 patient had allogenous transplantation)<br>• Chemotherapy aimed at suppressing lymphoid lineage: Bendamustine in 6 patients out of 24, Cyclophosphamide in 11 patients out of 24, Cyclophosphamide with Fludarabine in 1 patient out of 24, modified EPOCH in 3 patients out of 24, radiotherapy with Cyclophosphamide in 3 patients out of 24 (data about concentrations are not provided)	IIa	scFvCD19.4-1BB-CD3ξ<br>lentivirus	38 patients were included/<br>24 patients received infusion of T-lymphocytes with chimeric antigenic receptors (13 patients with DLBCL, 9 patients with FL and 2 patients with MCL)<br>Median age of patients is 56 years (ranging from 25 to 77 years)	Median value of cell dose is 5.84×10^6/kg (from 3.08×10^6/kg to 8.87×10^6/kg)	Median duration control follow-u was 11.7 month
**(Turtle et al. 2016) (registration number: NCT01865617)**	**CD19**<br>• Acute B-cell lymphoblastic leukaemia<br>•<br>• Infusion of T-lymphocytes with chimeric antigenic receptors 48 or 96 hours after chemotherapy aimed at suppressing lymphocyte lineage<br>• Patients' preparation:<br>• 2 patients out of 30 had Cyclophosphamide 2-4 mg/m^2 and Etoposide 100 mg/m2 x 3<br>• 11 patients out of 30 had Cyclophosphamide 2-4 mg/m2<br>• 5 patients out of 30 had Cyclophosphamide 30-60 mg/kg and Fludarabin 25 mg/m^2 x5<br>12 patients out of 30 had Cyclophosphamide 30-60 mg/kg x 1 and Fludarabin 25 mg/m^2 x3	I/IIa	scFvCD19.4-1BB-CD3ξ<br>Transgenic cells with CAR19 bound to self-detaching T2A sequence with clipped surface cell marker EGFR (EGFRt)	32<br>median age 42 years (from 20 to 73)	2×10^5/kg, 2×10^6/kg and 2×10^7/kg<br>T-lymphocyte populations enriched with CD4$^+$ and CD8$^+$ by positive immunomagnetic selection using CliniMACS CD4 Reagent system and 2-step selection procedure in CliniMACS device respectively<br>established ratio 1:1 for CD4$^+$/CD8$^+$ T-Tlymphocytes with	Median duration control follow-u was 300 days

					chimeric antigenic receptors	
**(Turtle et al. 2016) (registration number: <u>NCT01865617</u>)**	**CD19**<br>• Recurrent and/or refractory chronic non-Hodgkin lymphoma<br>• Large-cell B-cell lymphoma was first diagnosed in 11 patients out of 32<br>• Large-cell B-cell lymphoma developed from smoldering disease (TFLBCL) in 11 patients out of 32<br>• Mantle cell lymphoma in 4 patients out of 32<br>• Follicular lymphoma in 6 patients out of 32<br><br>• 16 patients out of 32 had relapse after autologous (in 14 out of 16) or allogenous (in 4 out of 16) hematopoietic stem cell transplantation<br>• Bone marrow damage in 9 patients out of 32<br>• Lymphoma in 1 patient out of 32 was diagnosed with cerebrospinal fluid (CSF) flow cytometry<br><br>• Therapy aimed at suppressing lymphocyte lineage, based on Cyclophosphamide (cy) with or without Fludarabine (Flu)<br><u>Group 1:</u><br>12 patients out of 32 received chemotherapy with Cyclophosphamide 2-4 g/m^2 intravenously on Day 1 (cy) or Cyclophosphamide 2-4 g/m2 intravenously on Day 1 and Etoposide 100-200 mg/m^2/day intravenously on Day 1-3 (Cy/E)<br><u>Group 2:</u><br>20 patients out of 32 received Cyclophosphamide 60 mg/kg intravenously on Day 1 or Fludarabine 25 mg/m^2/day intravenously on Day 2-4 or on Day 2-6 (Cy/Flu)	I	scFvCD19.4-1BB-CD3ξ lentivirus<br><br>Superficial cell marker EGFR (EGFRt)	37 included/32 received therapy<br><br>Median age of patients is 57 years (ranging from 22 to 70 years)	2x10^5 EGFRt$^+$ cells per 1 kg of body mass, 2x10^6 EGFRt$^+$ cells per 1 kg of body mass or 2x10^7 EGFRt$^+$ cells per 1 kg of body mass<br><br>established ratio in case of immunomagnetic separation (CliniMACS) 1:1 for CD4$^+$/CD8$^+$ T-lymphocytes with chimeric antigenic receptors	median control follow-up with C[illegible] or Cy/E therapy [illegible] months, with Cy[illegible] therapy 6.3 mont[illegible]
**(Brudno et al. 2016) (registration number: not provided)**	**CD19**<br>• 5 patients out of 20 with acute lymphatic leukaemia<br>• 5 patients out of 20 with chronic lymphatic leukaemia<br>• 5 patients out 20 with diffuse large cell B-cell lymphoma<br>• 5 patients out of 20 with mantle cell lymphoma<br><br>• Patients had previously received HLA-compatible hematopoietic stem cell allotransplantation from a sibling/unrelated donor<br><br>Chemotherapy or other therapies were not applied	I	scFvCD19.CD28-CD3ξ retrovirus	20	Increase in dose:<br>From 0.4×10^6/kg to 8.2x10^6/kg<br>(single infusion)	36 months

**(Kebriaei et al. 2016) (registration number: NCT00968760, registration number: NCT01497184, registration number: NCT01492036)**	**CD19**<br>• Non-Hodgkin lymphoma in 9 patients out of 26<br>• Acute lymphocytic leukaemia in 17 patients out of 26<br>• Autologous (in 7 patients out of 26) or allogenous (in 19 patients out of 26) transplantation of hematopoietic stem cells with infusion of T-lymphocytes with chimeric antigenic receptors as adjuvant therapy<br>• Patients' preparation:<br>◦ Part of the study with autologous transplantation: BEAM: Beginning of the therapy on Day -6 with intravenous administration of Carmustine (300 mg/m2) with subsequent administration of Etoposide (200 mg/m2) and Cytarabine (mg/m2) every 12 hours, a total of 8 doses on Days from -5 to -2, with subsequent administration of Melphalan (dose 140 mg/m2) on Day -1. Infusion of peripheral blood autologous stem cells was carried out on Day 0 with premedication using diphenhydramine only<br>◦ *Part of the study with allogenous* transplantation:<br>◦ after transplantation of HLA-compatible hematopoietic stem cells: prevention of "graft versus host" reaction included Tacrolimus (on days -2 – 180 after transplantation) and mini-dose of Methotrexate (5 mg/m^2 on Days 1, 3, 6 and 11 after infusion of peripheral blood stem cells)<br>◦ after haplo-identical transplantation of hematopoietic stem cells: Tacrolimus, Mycophenolate Mofetil, and after hemopoiesis stem cell transplantation - Cyclophosphamide (infusion of 50 mg/kg on Day 3 and 4 after peripheral blood stem cell infusion) Prevention of the reaction ("graft versus host") gradually decreased and was cancelled 6 months after hematopoietic stem cell transplantation, to the extent possible	I	scFvCD19.CD28-CD3ξ electroporation	26	Autologous T-lymphocytes, dose from $1x10^7/m^2$ to $5x10^7/m^2$<br><br>Allogenous T-lymphocytes, dose from $1x10^6/m^2$ to $1x10^8/m^2$<br><br>(scheme for increasing doses in different patients: number of CD3+ T-lymphocytes expressed in terms of the estimated surface area of the body)	Autologous HSC<br>median control follow-up 25.5 months (from 6.4<br>32.7 months)<br><br>Allogenous HSC<br>median control follow-up 7.5 months<br>(from 2.7 to 17.9<br>months)
**(Wang et al. 2016) (registration number: NCT01815749)**	**CD19**<br>• 4 patients out of 8 with mantle cell lymphoma<br>• 4 patient out of 8 had diffuse large cell B-cell lymphoma<br>• Cytoreductive chemotherapy (in the absence of reserve therapy) and granulocyte colony-stimulating factors and/or Plerixafor were administered<br>• Hematopoietic stem cell transplantation 2 days before infusion of T-lymphocytes with chimeric antigenic receptors<br>• All patients received bis-chloroethylnitrosourea, Etoposide, Ara-C and Melphalan (data about concentrations were not provided)	I	scFvCD19.CD28-CD3ξ lentivirus	8	$50×10^6$ and $200×10^6$ T-lymphocytes with chimeric antigenic receptors during one infusion	14 months
**(Teachey et al. 2016) (registration number: NCT01626495, registration number: NCT02030847 and registration number: NCT01029366)**	**CD19**<br>• ALL (acute lymphoblastic leukaemia)<br>• Complete remission after previous allogenous hematopoietic stem cell transplantation in 31 patient out of 51 (27 paediatric; 4 adults; 61%)<br>• 4 patients out of 51 (all paediatric) had previously received Blinatumomab, CD19 BITE antibody<br>• Before receiving CTL019 drug none of the patients received therapy with other drugs targeted to CD19	I/IIa	scFvCD19.4-1BB-CD3ξ lentivirus	51<br><br>39 patients out of 51 are paediatric<br><br>12 patients out of 51 are adults	$1\text{-}10x10^7$ T-lymphocyte/kg ($5\text{-}50x10^8$ T-lymphocytes for patients with body mass over 50 kg) within 1-3 days	
**(Cai et al. 2016) (registration number: NCT02799550)**	**CD19**<br>• Recurrent and refractory acute lymphatic leukaemia<br>• chemotherapy was used for repeated induction (Days -6 to -2) with Vindesine, Idarubicin, Pegasaprase and Dexamethasone (data about concentration were not provided)<br>• Concomitant infusion of T-lymphocytes with chimeric antigenic receptors obtained from a haplo-identical donor (Days 0-3) and mobilised peripheral blood stem cells (Day 2) after induction chemotherapy	I	scFvCD19.4-1BB-CD3ξ lentivirus	1 (woman aged 71 years)	• $0.5×10^8$ Day 0), $3×10^8$ (Day 1), $5×10^8$ (Day 2) and $9×10^8$ (Day 3) T-lymphocytes with chimeric antigenic receptors<br>• mononuclear cell infusion, dose $1.82×10^8$/kg, $CD34^+$ cells, dose $1.93×10^6$/kg and $CD3^+$ cells in	Until Day 31 (di<br>due to severe infection)

					granulocyte stem cells of peripheral blood, dose 0.46×10^8/kg (Day 2)	
**(Grupp et al. 2016) (registration number: not provided)** ***Abstract for ASH***	**CD19**<br>• Recurrent/refractory acute lymphatic leukaemia<br>• Chemotherapy to suppress lymphocyte lineage cells (see Maude et al. 2014)	I	scFvCD19.4-1BB-CD3ξ lentivirus	57 patients included/34 patients received infusion of T-lymphocytes with chimeric antigenic receptors<br><br>Median age of patients is 11 years (ranging from 3 to 23 years)	Median cell dose was $2.9\text{x}10^6$/kg (from 0.2×10^6/kg to $4\text{x}10^6$/kg)	Median duration control follow-up was 11.7 months
**(Maude et al. 2016) (registration number: not provided)** ***Abstract for ASH***	**CD19**<br>• Recurrent/refractory acute lymphatic leukaemia<br>• Chemotherapy to suppress lymphocyte lineage cells (see Maude et al. 2014)<br>• 18 patients out of 30 had previously received allogenous stem cell transplantation<br>• 11 patients out of 30 had not previously received T-lymphocytes with chimeric antigenic receptors to CD19 extracted from mice (CTL019 preparation, 7 patients out of 11; other preparations 4 patients out of 11)<br>• 6 patients out of 30 had damage of the central nervous system	I	humanised scFvCD19.4-1BB-CD3ξ lentivirus	30<br><br>29 months – 4 years	Median cell dose was $2.9\text{x}10^6$/kg (from 0.22×10^6/kg (from 0.2×10^6/kg to 4×10^6/kg)	median duration control follow-up was 4.2 months (1.0-14.1 months
**(Neelapu et al. 2016) (registration number: NCT02348216)** ***Abstract for ASH***	**CD19**<br>• Diffuse large cell B-cell lymphoma<br>• Cyclophosphamide (500 mg/m^2) and Fludarabine (30 mg/m^2) daily for 3 days<br>• patients had previously received antibodies neutralising CD20, and therapy regimens containing Anthracycline	II	scFvCD19.CD28-CD3ξ lentivirus	51<br><br>Median age of patients is 58 years (ranging from 25 to -76 years)	2 $\times10^6$/kg (from 0.2×10^6/kg to 4×10^6/kg)	minimal duration control follow-up was 3 months

**(Locke et al. 2017) (registration number: NCT02348216)**	**CD19**<br>• Diffuse large cell B-cell lymphoma<br><br>Preparatory chemotherapy with low doses of Cyclophosphamide (500 mg/m^2) and Fludarabine (30 mg/m^2) for 3 days	I	scFvCD19.CD28-CD3ξ	7	$2x10^6$ T-lymphocytes with chimeric antigenic receptors per 1 kg of body mass<br>(single infusion)	Median duration control follow-u[illegible] was 9 months/ long-term contro[illegible] follow-up for 15 years
**(Kochenderfer et al. 2017) (registration number: NCT00924326)**	**CD19**<br>• 19 patients out 22 with diffuse large cell B-cell lymphoma<br>• 2 patients out of 22 with follicular lymphoma<br>• 1 patient out of 22 with mantle cell lymphoma<br><br>Patients received T-lymphocytes with chimeric antigenic receptors once after preparatory chemotherapy in low doses, which included Cyclophosphamide in combination with Fludarabine (no data about concentrations were reported)	I	scFvCD19.CD28-CD3ξ retrovirus	22	$1×10^6$, $2×10^6$, $6×10^6$, T-lymphocytes with chimeric antigenic receptors per 1 kg of body mass	24 months
**(Qasim et al. 2017) (prior to the schedule clinical study of phase I NCT02808442)**	**CD19**<br>• recurrent/refractory acute lymphatic leukaemia, due to which patients had previously received chemotherapy and stem cell allotransplantation<br><br>• Residual T lymphocytes with α and β T cell receptors were completely removed by binding to magnetic particles in CliniMACS device to minimise the risk of graft versus host reaction<br>• Decrease in the volume of lymphocyte cells for 7 days before infusion of T-lymphocytes with chimeric antigenic receptors by using Fludarabine (total dose 90 mg/m^2 (S1) or 150 mg/m^2 (S2), Cyclophosphamide (total dose 1.5 g/m^2 (S1) or 120 mg/kg (S2)), and Atezolizumab (total dose 1 mg/kg)<br><br>Prior to the second stem cell allotransplantation:<br>Patient 1:<br>• preparatory therapy using anti-thymus globulin (ATG) (total dose 4.5 mg/kg), Fludarabine (total dose 120 mg/kg), Cyclophosphamide (total dose	*prior to study of phase I*	scFvCD19.4-1BB-CD3ξ<br><br>The third generation of self-inactivating lentiviral vector: a transgene encoding CAR19, bound to a self-detaching peptide to the sorting/suicide gene RQR8, incorporated into CD34 epitope to increase the number of cells affected by antibodies to CD34 (Qbend10) and to the targeted epitope CD20 for antibodies to CD20 (Rituximab) for in vivo use to reduce levels of such cells<br><br>electroporation of TALEN mRNA into lentivirus-transduced T lymphocytes with chimeric antigenic receptors to CD19:	2 (babies)<br><br>Patient 1: 11-month old child of mixed race<br><br>Patient 2: 16-month old child of Caucasian parents	Bank of HLA-incompatible cells with universal chimeric antigenic receptors to CD19 (UCART19) T-lymphocytes were obtained from a healthy female donor<br><br>Patient 1:<br>$4.6x10^6$ T-lymphocytes with chimeric antigenic receptors to CD19 per 1 kg of body mass<br><br>Patient 2:<br>$4.0x10^6$ T-lymphocytes with chimeric antigenic receptors per 1 kg of body mass on a single occasion	18 months

	60 mg/kg), Thiotepa (total dose 5 mg/kg), and whole body radiation therapy (4 Gy)<br><br><u>Patient 2:</u><br>• Rituximab (375 mg/m^2), immediately followed by preparatory therapy using anti-thymus globulin (total dose 10 mg/kg), Fludarabine (total dose 120 mg/kg), Cyclophosphamide (total dose 120 mg/kg), and whole body radiation therapy (2 Gy)		1) TALEN pair with targeted effect on the constant part of the T-cell receptor of α chain (with impaired expression of T-cell receptors α and β (TCR αβ) on the cell surface)<br>2) TALEN pair aimed at breaking the CD52 gene (designed to improve survival in the presence of anti-CD52 antibodies inhibiting the lymphocytic lineage (Alemtuzumab))			
**(Gardner et al. 2017) (registration number: <u>NCT02028455</u>)**	**CD19**<br>• recurrent/refractory acute lymphatic leukaemia<br><br>• 28 patients out of 45 had had at least one allogenous cell transplantation before<br>• 6 patients out of 45 had received Blinatumomab before<br>• 1 patients out of 45 had previously received CD19-specific T-lymphocytes with chimeric antigenic receptors of the second generation (CD28zeta)<br><br>The first cohorts received antilymphocyte therapy at the discretion of the attending physician: The recommended therapy for inhibiting the lymphocyte lineage consisted of Cyclophosphamide monotherapy; Cyclophosphamide in high dose (from 2 to 4 mg/m^2) in combination with Mesna drug; Fludarabine with or without Cytarabine; and Cyclophosphamide/Etoposide. After amendment to the protocol, patients included in the antilymphocyte therapy group had to receive Fludarabine 30 mg/m^2 once a day for 4 days (Days 1-4) in combination with Cyclophosphamide 500 mg/m^2 once a day on Days 3 and 4 (a total of 2 doses).	I/II	scFvCD19.4-1BB-CD3ξ lentivirus<br><br>Transgenic cells with CAR19 bound to self-detaching T2A sequence with clipped surface cell marker EGFR (EGFRt)	45<br>(from ≥1 to 26 years)<br><br>median age 12.3 years (ranging from 1.3 to 25.4 years), including 4 patients below 3 years	0.5×10^6-5.0×10^6 T-lymphocytes with chimeric antigenic receptors per 1 kg of body mass<br><br>established ratio in case of immunomagnetic separation (CliniMACS) 1:1 for $CD4^+$/$CD8^+$ T-lymphocytes with chimeric antigenic receptors<br><br>EGFRt immunomagnetic selection to increase T-lymphocyte count with transgene expression (CliniMACS)	28 months
**(Fitzgerald et al. 2017) (registration number: <u>NCT01626495</u>) (retrospective cohort study)**	**CD19**<br>• recurrent/refractory acute lymphatic leukaemia<br><br>23 patients out of 39 had a relapse after allogenic hematopoietic stem cell transplantation	I/IIa	scFvCD19.4-1BB-CD3ξ lentivirus	39<br>Median age of patients is 11 years (ranging from 5 to 22 years)	From $1x10^7$ to $10x10^7$ autologous T-lymphocytes per 1 kg of body mass within 1-3 days	28 days

**(Pan et al. 2017) (registration number: <u>ChiCTR-IIh-16008711)</u>**	**CD19**<br>• recurrent/refractory acute lymphatic leukaemia<br><br>Acute lymphocytic leukaemia with primary refractoriness or hematologic relapse in 42 patients out of 51<br>Minimal residual disease was found in flow cytometry in 9 patient out of 51 (FCM-MRD^{+})<br>• Therapy aimed at suppressing lymphocyte lineage included Fludarabine (30 mg/m^{2}/day) and Cyclophosphamide (250 mg/m^{2}/day) on Days -5, -4 and -3, except for female patients with persistent cytopenia	I	scFvCD19.4-1BB-CD3ξ lentivirus	51<br>(from 3 to 60 years)	The doses initially ranged from 0.05×10^{5}/kg to 14×10^{5}/kg, then they were set at the level of 1×10^{5}/kg for most patients out of the last 20	Median 206 days<br>(from 45 to 427)
**(Turtle et al. 2017)**	**CD19**	I/II	scFvCD19.4-1BB-CD3ξ lentivirus	24	2×10^{5}, 2×10^{6} or 2×10^{7} T-lymphocytes with	28 months

**(registration number: NCT01865617)**	• Chronic lymphocytic leukaemia refractory to Ibrutinib<br><br>• All patients received therapy earlier, median number of treatment lines is 5 (ranging from 3 to 9)<br>• All patients received Ibrutinib (median duration of therapy was 13 months, ranging from 0.75 to 39 months)<br>• Disease progression in 19 patients out of 24 against the background of Ibrutinib therapy (Bruton tyrosine kinase inhibitor)<br>• 3 patients out of 24 had intolerance to Ibrutinib<br>• No disease progression in 2 patients out of 24 against the background of Ibrutinib therapy<br>• 23 patients out of 24 were refractory to therapy with Fludarabine and Rituximab or had a relapse against the background of this therapy<br>• 6 patients out of 24 were refractory to Venetoclase (BCL2 inhibitor)<br>• 23 patients out of 24 had a complicated karyotype and/or 17p deletion<br>• 23 out of 24 patients had lymph node involvement<br>• 2 patients out of 24 had previously had central nervous system damage<br><br>• Prior to therapy aimed at suppressing the lymphocyte lineage, the use of Ibrutinib was discontinued<br>• Antilymphocyte therapy included Cyclophosphamide (Cy) or Fludarabine (Flu), or a combination of both<br>• 1 patients out of 24 received Cyclophosphamide 2 g/m2<br>• 2 patients out of 24 received Fludarabine 25 mg/m2 (a total of 3 doses)<br>• 3 patients out of 24 had Cyclophosphamide 30 mg/m2 and Fludarabin three times 25 mg/m2<br>• 14 patients out of 24 had Cyclophosphamide 60 mg/m2 + Fludarabin three times 25 mg/m2<br>• 1 patient out of 24 had Cyclophosphamide 60 mg/m2 + Fludarabin 5 times 25 mg/m^2<br>• 2 patients out of 24 had Cyclophosphamide three times 500 mg/m2 + Fludarabin three times 25 mg/m2<br>• 1 patient out of 24 had Cyclophosphamide 60 1 g/m2 + Fludarabin three times 25 mg/m2		Superficial cell marker EGFR (EGFRt)	Patients' median age 61 years (ranging from 40 to 73)	chimeric antigenic receptors per 1 kg of body mass<br><br>established ratio 1:1 for $CD4^+$/$CD8^+$ T-Tlymphocytes with chimeric antigenic receptors<br><br>6 patients out of 24 who had a persistent or recurrent disease after the initial assessment of the disease stage received the second cycle of antilymphocyte therapy and infusion of T lymphocytes with chimeric antigenic receptors	
**(Hu et al. 2017)**<br>**(registration number: ChiCTR-OCC-15007008)**	**CD19**<br>recurrent/refractory acute lymphatic leukaemia<br><br>• 4 patients out of 15 had acute lymphatic leukaemia with the Philadelphia chromosome<br>• ABLT3151 mutation in 2 patients out of 15<br>• 5 patients out of 15 had previously received allogenic hematopoietic stem cell transplantation<br>• 4 patients out of 15 had previously had central nervous system damage<br>• 2 patients out of 15 had extramedullar relapse in testes<br><br>• Antilymphatic therapy included Cyclophosphamide and Fludarabine<br>• 6 patients out of 15 received Fludarabin 30 mg/m^2 on Days from -6 to -4 and Cyclophosphamide 1 g/m^2 on Days from -3 to -2		scFvCD19.4-1BB-CD3ξ lentivirus	15<br>Patients' median age 32 years (ranging from 7 to 57)	1.1×10^6/kg – 9.8×10^6/kg	Median duration control follow-u was 142 days

	• 5 patients out of 15 received Fludarabin 30 mg/m^2 on Days from -4 to -2 and Cyclophosphamide 750 g/m^2 on Day -2<br>• 4 patients out of 15 received Fludarabin 25 mg/m^2 on Days from -8 to -4 and Cyclophosphamide 750 g/m^2 on Days from -3 to -2					
