# Supplementary material for: Multiple site place-of-care manufactured anti-CD19 CAR-T cells induce high remission rates in B-cell malignancy patients
Source: Nat Commun. 2021 Dec 10;12:7200. doi: 10.1038/s41467-021-27312-6 (PMC8664838; doi:10.1038/s41467-021-27312-6)
Supplement: Supplementary file 2 — Reporting Summary [file 41467_2021_27312_MOESM2_ESM.pdf]

nature portfolio

Corresponding author(s): Marcos de Lima

Last updated by author(s): Oct 23, 2021

Reporting Summary

Nature Portfolio wishes to improve the reproducibility of the work that we publish. This form provides structure for consistency and transparency in reporting. For further information on Nature Portfolio policies, see our Editorial Policies and the Editorial Policy Checklist.

Statistics

For all statistical analyses, confirm that the following items are present in the figure legend, table legend, main text, or Methods section.

n/a	Confirmed	
[ ]	[x]	The exact sample size (n) for each experimental group/condition, given as a discrete number and unit of measurement
[x]	[ ]	A statement on whether measurements were taken from distinct samples or whether the same sample was measured repeatedly
[ ]	[x]	The statistical test(s) used AND whether they are one- or two-sided<br>*Only common tests should be described solely by name; describe more complex techniques in the Methods section.*
[ ]	[x]	A description of all covariates tested
[x]	[ ]	A description of any assumptions or corrections, such as tests of normality and adjustment for multiple comparisons
[ ]	[x]	A full description of the statistical parameters including central tendency (e.g. means) or other basic estimates (e.g. regression coefficient) AND variation (e.g. standard deviation) or associated estimates of uncertainty (e.g. confidence intervals)
[ ]	[x]	For null hypothesis testing, the test statistic (e.g. F, t, r) with confidence intervals, effect sizes, degrees of freedom and P value noted<br>*Give P values as exact values whenever suitable.*
[x]	[ ]	For Bayesian analysis, information on the choice of priors and Markov chain Monte Carlo settings
[x]	[ ]	For hierarchical and complex designs, identification of the appropriate level for tests and full reporting of outcomes
[x]	[ ]	Estimates of effect sizes (e.g. Cohen's d, Pearson's r), indicating how they were calculated

Our web collection on statistics for biologists contains articles on many of the points above.

Software and code

Policy information about availability of computer code

Data collection	No software was used for data collection
Data analysis	*Provide a description of all commercial, open source and custom code used to analyse the data in this study, specifying the version used OR state that no software was used.*

For manuscripts utilizing custom algorithms or software that are central to the research but not yet described in published literature, software must be made available to editors and reviewers. We strongly encourage code deposition in a community repository (e.g. GitHub). See the Nature Portfolio guidelines for submitting code & software for further information.

Data

Policy information about availability of data

All manuscripts must include a data availability statement. This statement should provide the following information, where applicable:

- Accession codes, unique identifiers, or web links for publicly available datasets
- A description of any restrictions on data availability
- For clinical datasets or third party data, please ensure that the statement adheres to our policy

Outcome data will be available by direct request to corresponding author, on a case by case basis, respecting privacy laws (no identifiers) and upon establishment of all legal requirements for data transfer agreements

Field-specific reporting

Please select the one below that is the best fit for your research. If you are not sure, read the appropriate sections before making your selection.

[x] Life sciences [ ] Behavioural & social sciences [ ] Ecological, evolutionary & environmental sciences

For a reference copy of the document with all sections, see nature.com/documents/nr-reporting-summary-flat.pdf

Life sciences study design

All studies must disclose on these points even when the disclosure is negative.

Sample size	Sample size in phase I trials is variable and done according to dose escalation rules. There is no sample size calculation
Data exclusions	no data was excluded from analyis
Replication	preclinical experiments were reproducible and performed independently; the clinical trials and this report are a demonstration of clinical reproducibility in separate sites of manufacture of CAR-T cells with clinical activity.
Randomization	These trials were phase I trials. Randomization is not part of the methodology in dose - escalation phase I studies
Blinding	These trials were phase I trials. Blinding is not part of the methodology in dose - escalation studies; blinding can affect safety of trial participant and impedes dose escalation desitions in trial conduct

Reporting for specific materials, systems and methods

We require information from authors about some types of materials, experimental systems and methods used in many studies. Here, indicate whether each material, system or method listed is relevant to your study. If you are not sure if a list item applies to your research, read the appropriate section before selecting a response.

Materials & experimental systems

n/a	Involved in the study	
[ ]	[ ]	Antibodies
[ ]	[x]	Eukaryotic cell lines
[ ]	[ ]	Palaeontology and archaeology
[ ]	[x]	Animals and other organisms
[ ]	[x]	Human research participants
[ ]	[x]	Clinical data
[ ]	[ ]	Dual use research of concern

Methods

n/a	Involved in the study	
[x]	[ ]	ChIP-seq
[x]	[ ]	Flow cytometry
[x]	[ ]	MRI-based neuroimaging

Antibodies

Antibodies used	*Describe all antibodies used in the study; as applicable, provide supplier name, catalog number, clone name, and lot number.*
Validation	*Describe the validation of each primary antibody for the species and application, noting any validation statements on the manufacturer's website, relevant citations, antibody profiles in online databases, or data provided in the manuscript.*

Eukaryotic cell lines

Policy information about cell lines

Cell line source(s)	ATCC, Manassas, Virginia, USA
Authentication	None of the cell lines used were authenticated
Mycoplasma contamination	*Confirm that all cell lines tested negative for mycoplasma contamination OR describe the results of the testing for mycoplasma contamination OR declare that the cell lines were not tested for mycoplasma contamination.*
Commonly misidentified lines (See ICLAC register)	*Name any commonly misidentified cell lines used in the study and provide a rationale for their use.*

Palaeontology and Archaeology

Specimen provenance	*Provide provenance information for specimens and describe permits that were obtained for the work (including the name of the issuing authority, the date of issue, and any identifying information). Permits should encompass collection and, where applicable, export.*
Specimen deposition	*Indicate where the specimens have been deposited to permit free access by other researchers.*
Dating methods	*If new dates are provided, describe how they were obtained (e.g. collection, storage, sample pretreatment and measurement), where they were obtained (i.e. lab name), the calibration program and the protocol for quality assurance OR state that no new dates are provided.*

☐ Tick this box to confirm that the raw and calibrated dates are available in the paper or in Supplementary Information.

Ethics oversight	*Identify the organization(s) that approved or provided guidance on the study protocol, OR state that no ethical approval or guidance was required and explain why not.*

Note that full information on the approval of the study protocol must also be provided in the manuscript.

Animals and other organisms

Policy information about studies involving animals; ARRIVE guidelines recommended for reporting animal research

Laboratory animals	Mice, NSG, all female, 6 - 8 weeks old
Wild animals	*Provide details on animals observed in or captured in the field; report species, sex and age where possible. Describe how animals were caught and transported and what happened to captive animals after the study (if killed, explain why and describe method; if released, say where and when) OR state that the study did not involve wild animals.*
Field-collected samples	*For laboratory work with field-collected samples, describe all relevant parameters such as housing, maintenance, temperature, photoperiod and end-of-experiment protocol OR state that the study did not involve samples collected from the field.*
Ethics oversight	Studies conduced under approval by Institutional Animal Care and Use Committee of Case Western Reserve University

Note that full information on the approval of the study protocol must also be provided in the manuscript.

Human research participants

Policy information about studies involving human research participants

Population characteristics	Two separate trials included patients diagnosed with acute lymphoblastic leukemia, ages 1 - 20, 29% female, with relapsed or refractory disease to to at least 2 lines of therapy.The non Hodgkin lymphoma cohort included adult patients ages 33 - 76 years of age, 35% female, previously treated with at least 2 lines of therapy
Recruitment	Subject swere enrolled in the clinical trials through IRB approved protocols and consenting processes. Participation in the clinical trial was offered by their clinical care team and was voluntary.
Ethics oversight	Institutional review board of Dmitriy Rogachev Center - ALL trialInstitutional review board of University Hospitals Cleveland Medical Center - NHL trial

Note that full information on the approval of the study protocol must also be provided in the manuscript.

Clinical data

Policy information about clinical studies

All manuscripts should comply with the ICMJEguidelines for publication of clinical research and a completedCONSORT checklist must be included with all submissions.

Clinical trial registration	clinicaltrials.gov NCT03467256 and NCT03434769
Study protocol	Study protocols provided during first submission of this manuscript
Data collection	Pediatric acute leukemia patients were treated at Dmitriy Rogachev Center in Moscow, Russia. Enrollment occurred between 2017 and 2020. Non Hodgkin lymphoma patients were treated at University Hospitals Cleveland Medical Center, in Cleveland, United States. Enrollment occurredbetween 2018 and 2020.
Outcomes	ALL trial: Primary objectives included to investigate the safety of CD19 CAR-T cells, the efficacy of CAR-T cells in ALL and the long term efficacy of CAR-T cells in ALL. The outcome measures include the incidence of adverse events on the first month of treatment, including severe cytokine release syndrome and the proportion of patients in remission without evidence of minimal residual disease. Secondary outcome measures include duration of minimal residual disease free remission, persistence of CAR-T cells, duration of B cell aplasia and overall survival.NHL trial: Primary objectives is to determine the safety of antiCD19 CAR-T cells in treatment of NHL. Secondary objectives include the description of the safety profile of infusion of CAR-T cells, toxicities related to infusion and the overall response rate and complete response rates. The primary outcomes measure include number of patients with lymphoma response. Duration of response (time from achievement of complete or partial response until the time of relapse or censoring),

disease free survival (time from study entry to death or disease recurrence), progression free survival (time from study entry to death or progression from any cause), time to progression (time from study entry to lymphoma progression or death), and time to treatment failure (time from study entry to any treatment failure)

Dual use research of concern

Policy information about dual use research of concern

Hazards

Could the accidental, deliberate or reckless misuse of agents or technologies generated in the work, or the application of information presented in the manuscript, pose a threat to:

No	Yes	
☒	☐	Public health
☒	☐	National security
☒	☐	Crops and/or livestock
☒	☐	Ecosystems
☒	☐	Any other significant area

Experiments of concern

Does the work involve any of these experiments of concern:

No	Yes	
☒	☐	Demonstrate how to render a vaccine ineffective
☒	☐	Confer resistance to therapeutically useful antibiotics or antiviral agents
☒	☐	Enhance the virulence of a pathogen or render a nonpathogen virulent
☒	☐	Increase transmissibility of a pathogen
☒	☐	Alter the host range of a pathogen
☒	☐	Enable evasion of diagnostic/detection modalities
☒	☐	Enable the weaponization of a biological agent or toxin
☒	☐	Any other potentially harmful combination of experiments and agents
